# Supplementary material for: Biotransformation of polyunsaturated fatty acids to bioactive hepoxilins and trioxilins by microbial enzymes
Source: Nat Commun. 2018 Jan 9;9:128. doi: 10.1038/s41467-017-02543-8 (PMC5760719; doi:10.1038/s41467-017-02543-8)
Supplement: Supplementary file 1 — Supplementary Information [file 41467_2017_2543_MOESM1_ESM.pdf]

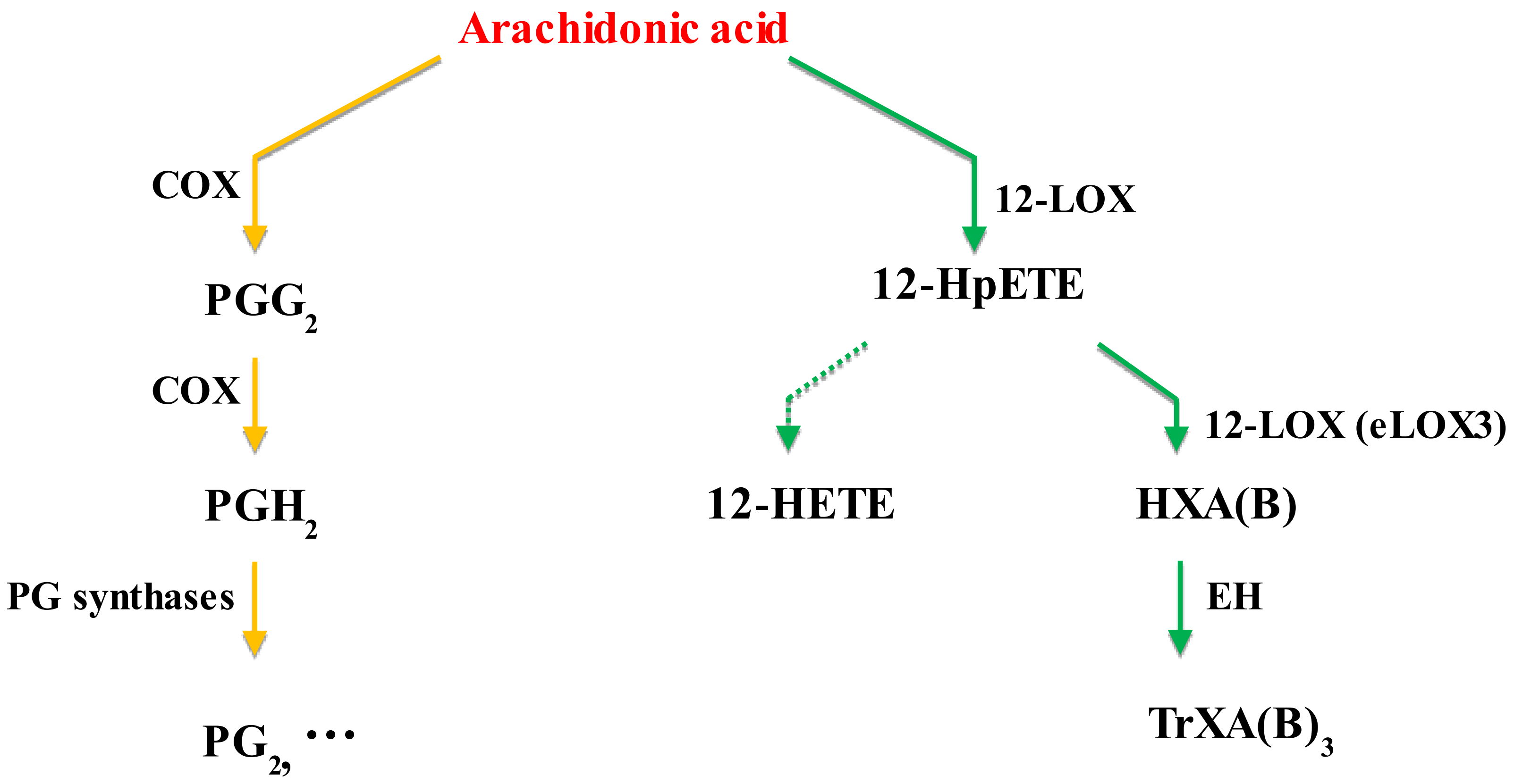

Supplementary Figure 1. Pathways of trioxilins and prostaglandins biosynthesis in human. TrXs: trioxilins; PGs: prostaglandins.

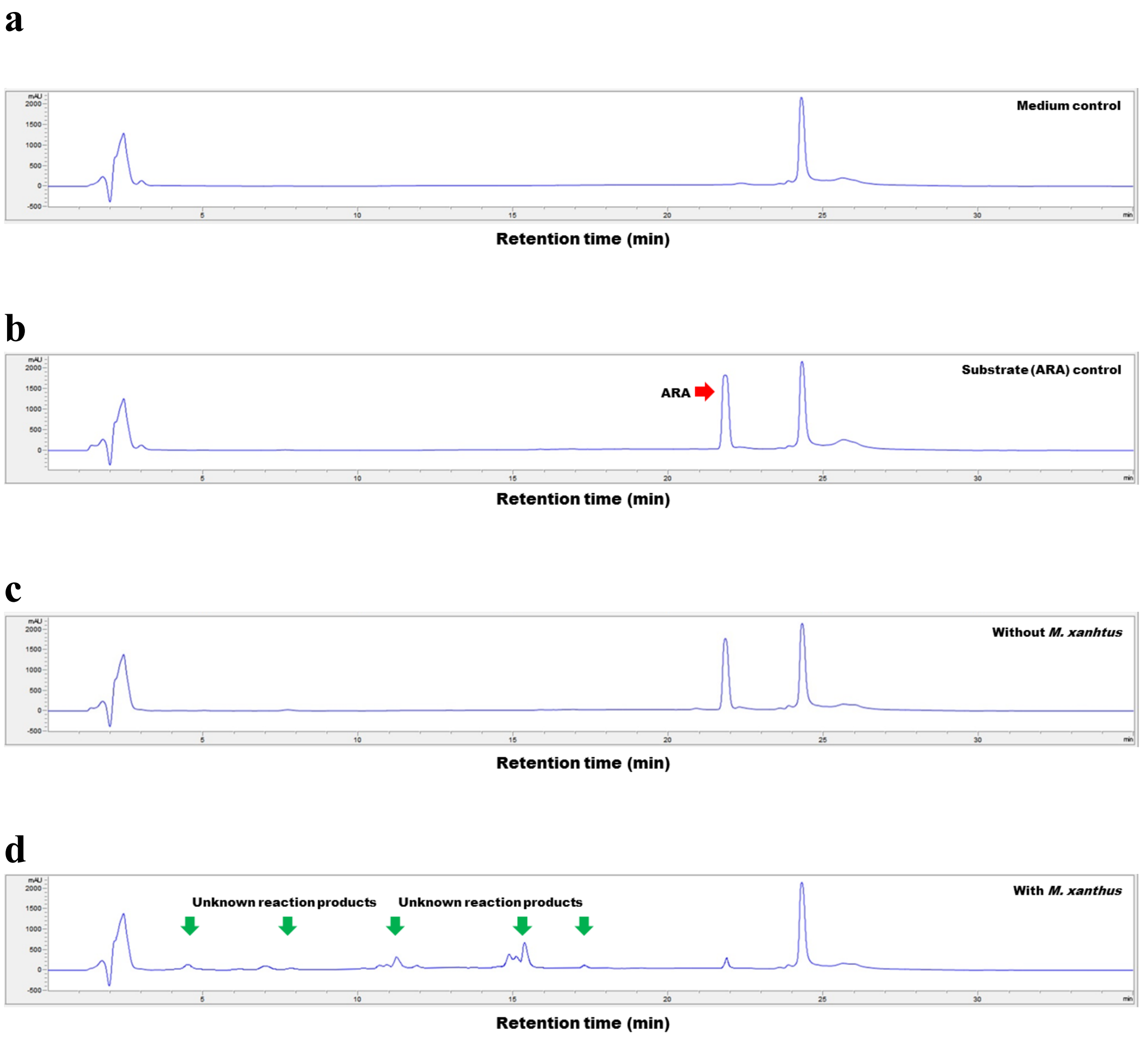

Supplementary Figure 2. HPLC analysis of *Myxococcus xanthus* cultivation with arachidonic acid. **(a)** HPLC analysis of only culture medium. **(b)** HPLC analysis of culture medium containing 1 mM arachidonic acid (ARA). **(c)** HPLC analysis of culture medium containing 1 mM ARA without wild-type *M. xanthus* after 24 h incubation. **(d)** HPLC analysis of fermentation broth containing 1 mM ARA with wild-type *M. xanthus* after 24 h incubation. The fermentations were performed 30 °C in Casitone broth at 200 rpm.

a

```
HU 12LOX    1  ----------------------MGRYRIRVATGAWLF----SG----S-----YNRVQLW   25
MX   1744   1  ---------------------MTVEYKLTIRTGTKLG----AG----T-----DADISIV   26
MX   1745   1  MSASVTRRGGADDRRWDGRARGMGTGMMFAGLRRWMGALGGKGRESGSNEVLDAEELSRW   60
                                          :       :      *    :        :.

HU 12LOX   26  LVGTRGEAELELQLRPARG-E----EEEFDHDVAEDLGLLQFVRLRKHHW-----LVDDA   75
MX   1744  27  LVGTRGESAPRVLDKHFHN-DFEAGAEDVYALSSEDLGDLVLLRFSNAGG------VAAD   79
MX   1745  61  YSGLALEERLAISRELAPRVRAVRPAREPSTLPAVAVGRLVFEQDGPQGPIPMHHIKVEL  120
                 *   *    :  .           .:     :  :* * : :

HU 12LOX   76  WFCDRITVQ--------GPGACAEVAFPCYRWVQGEDILSLPEGTARLPGDNALDMFQKH  127
MX   1744  80  WLLDWAIVT----------AGEKQWHFPFYRWVLSGATVDVLEGTAKLARQASSERESTA  129
MX   1745 121  WDRDFGTPDDFLGEGFTDSDGCFSIRYD-----PAD------AGVNDLPDLEV--RFFEP  167
               *  *                .  .  :       .        *.  *

HU 12LOX  128  REKELKDRQQIYCWAT------------------------W--KEGLPLTIAADRKDDLP  161
MX   1744 130  RRELLEARQRMYPWRA------------------------PEMTEGLPGALDLREGRPLP  165
MX   1745 168  QHSFRPDGRVVEAWCRIGSEKGPDDHGGLHYDFGTLRLPYWEYDPTTPLARLLVTEEGTP  227
               :..     : :  *                                 * :         *

HU 12LOX  162  PNMRFHEEKRLDFEWTLKAGALEMALKRVYTLLSSWNCLEDFDQIFWGQKSALAEKVRQC  221
MX   1744 166  KDELYRGLTEGSYEVVIAKT-LAAIKLNLPMLTRAWNGLVDIFDFFK---HLEVPQLAQR  221
MX   1745 228  PTAYAPG------------RALAMLKAVAPIEL------VK-------RRHLL-------  255
                                    *                  .

HU 12LOX  222  WQDDELFSYQFLNGANPMLLRRSTSLPSRLVLPS----GMEELQAQLEKELQNGSLFEAD  277
MX   1744 222  WKDDLEFARQAVQGIAPLHITLVPSLPQGMPLTDDDVRGLLSPGTTLARALDAKRIFLID  281
MX   1745 256  ---------QGRLGQAPSLDRIQADYPEAMTVRM----ERESPGSTRTDAFFGERLLNGM  302
                        *   *  *       . *. : :         .  :     :    ::

HU 12LOX  278  FIL-LDGIPA----------NV---IRGE-----KQYLAAPLVMLKMEPNGKLQPMVIQI  318
MX   1744 282  FEI-LDDIRM----------YR---KVGEDGVEERRWAPAARCLLYLDDQRQLRPLAIQL  327
MX   1745 303  FSTLMDGDPEAPGDPEAFRLYFPWNAYEQDGV---HCLPDVDVRLRL-VEGRLLPVRIIL  358
               *   :*.                     :      :        * :  : :* *: * :

HU 12LOX  319  QPPNPS-----SPTPTLFLPSDPPLAWLLAKSWVRNSDFQLHEIQYHLLNTHLVAEVIAV  373
MX   1744 328  GRDA--------QKDPVFTPNDDAYDWLAAKIYLRCSEGNSHQMVSHALRTHFVAEPFVM  379
MX   1745 359  GMREPGATAPGSPVTRRSYTPADGEAWEAAKRMARVSATLETELGNHLGQQHFNVEQYAI  418
                                         *  **   * *     ::  *  . *: .*  .:

HU 12LOX  374  ATMRCLPGLHPIFKFLIPHIRYTMEINTRARTQLISDGGIFDKAVSTGGG--GHVQLLRR  431
MX   1744 380  ATMRNLPDPHPVYKLLRRHFRYTLAINEGARKGLLDAGGVFDDFIATGGPDKGHLQLGKK  439
MX   1745 419  AAHRNLRR-SPLRWLLMPHLREVVLINHSANGFLVGPTGYITPASALTERSVETRLLHLM  477
               *: * *    *:  :*  *:* .: **  *.  *:.  * :    :          *

HU 12LOX  432  AAAQLTYCSLCPPDDLADRGLLG---LPGALYAHDALRLWEIIARYVEGIVHLFYQRDD-  487
MX   1744 440  GFQRWTLADNKPRADLERRGVLDPAVLPNYPYRDDALPLWDAFEEYVGGVLRHFYRTDA-  498
MX   1745 478  GSYDWKGFAPAP------------PICESHRYARAAGLFWRLVGEHVD----AFFAEHGA  521
               .    .     *                .  *   *  :*  . .:*      *:  .

HU 12LOX  488  IVKGD-PELQAWCREITEVGLC--------------------------------------  508
MX   1744 499  DLEAD-TEMQQWWKDLTEHGLP--------------------------------------  519
MX   1745 522  ALEAQWSEVRRFSDDLVGHSAPAFVCRYLRATVPGRAAPWFVRSERMDLDAKVAATHAKA  581
                ::.:  *:: :  ::.  .

HU 12LOX  509  ----QAQDRGFPVSFQSQSQLCHFLTMCVFTCTAQHAAINQGQLDWYAWVPNAPCTMRMP  564
MX   1744 520  ----VDKLP--CRELRRVDDLVDILTTVLFTVSVQHAAVNYLQYEHYAFVPNAPLSMRRE  573
MX   1745 582  VSAVTRTDAPQPGEMEALKQLCRY---VIYFATFFHAWANNLQWDDAGEVLYACLGLRWG  638
                            .:.  .:*       ::  : :**  *  * :  . *  *   :*

HU 12LOX  565  PPTTKEDVTMATVMGSLPDVRQACLQMAISWHLSRR---QPDMVPLGHHKEKYFSGPKPK  621
MX   1744 574  PPRQKGTLRAEDIPEMIPTKSQMLWQVAIGRALSSFGDDEEYLLHEGGWREEYFHEPELV  633
MX   1745 639  KAGA---LSTEADHDVAPPPEEATEMLWISWMLSKTSYG-------------FLLANEEA  682
                      :         *   :    : *.  **                  ::   :

HU 12LOX  622  AVLNQFRTDLEKLEKEITARNEQLDWPYEYLKPSCIENSVTI                    663
MX   1744 634  AIRQRFQERLRAQREAVEARNAGAEVPYTILRPDRIPCGITV                    675
MX   1745 683  DVHPRFVECLRAHAAEFSALGMDIR---------TVSSRINI                    715
                :  :*   *.     . * .              :   :.:
```

b

```
HU EPHX2   1  MTLRAAVFDLDGVLALPAVFGVLGRTEEALALPRGLLNDAFQKGGPEGATTRLMKGEITL   60
MX 1644    1  ------------------------------------------------------------    0

HU EPHX2  61  SQWIPLMEENCRKCSETAKVCLPKNFSIKEIFDKAISARKINRPMLQAALMLRKKGFTTA  120
MX 1644    1  ------------------------------------------------------------    0

HU EPHX2 121  ILTNTWLDDRAERDGLAQLMCELKMHFDFLIESCQVGMVKPEPQIYKFLLDTLKASPSEV  180
MX 1644    1  ------------------------------------------------------------    0

HU EPHX2 181  VFLDDIGANLKPARDLGMVTILVQDTDTALKELEKVTGIQLLNTPAPLPTSCNPSDMSHG  240
MX 1644    1  -----------------------------------------------------MADITHR    7
                                                                     :*::*

HU EPHX2 241  YVTVKPRVRLHFVELGSGPAVCLCHGFPESWYSWRYQIPALAQAGYRVLAMDMKGYGESS  300
MX 1644    8  T-VKTNGINLHLAEAGSGPLVLLLHGWPESWYSWRHQLPALAAAGYHAVAPDVRGYGQSD   66
                . .  :.**:.* **** * * **:********:*:**** ***:.:* *::***:*.

HU EPHX2 301  APPEIEEYCMEVLCKEMVTFLDKLGLSQAVFIGHDWGGMLVWYMALFYPERVRAVASLNT  360
MX 1644   67  KPEAIEAYSMKQLVGDAVGLLDALGERTAIVIGHDWGSAIAWNCAALHPDRFRAVVGMSV  126
               *  ** *.*: *  : * :** **   *:.******. :.*  * ::*:*.***..:..

HU EPHX2 361  PFIPANPNMSPLES--IKANPVFDYQLYFQEPGVAEAELEQNLSRTFKSLFRASDESVLS  418
MX 1644  127  PHLGRAPM-PPMQLFQRMFGEKWFYILYFQEPGVAEAEFEADVPRTVRAILTGTPGFDVT  185
              *.:   *   *::      .  : * ************:* :: **.:::: .:    ::

HU EPHX2 419  MH---KVCEAGGLFVNSPEEPSLSRMVTEEEIQFYVQQFKKSGFRGPLNWYRNMERNWKW  475
MX 1644  186  NPAVLAKKKGEGFLARLDVPETLPGWLTEADVAYFAKELAGSGFRGGLNFYRNMDRDWHE  245
                      :. *::..     :*   :** :: ::.:::  ***** ** ****:*:*:

HU EPHX2 476  ACKSLGRKILIPALMVTAEKDFVLVPQMSQHMEDWIPHLKRG-HIEDCGHWTQMDKPTEV  534
MX 1644  246  LPELATAVISQPALYIVGEKDPVRAFSPVDPMKALVPNLADIHVIPGAGHWVQQEHAAEV  305
                :     *  *** :..*** * . .  : *:  :*:*     * ..***.* :: :**

HU EPHX2 535  NQILIKWLDSDARNPPVVSKM                                        555
MX 1644  306  NAALLAFLKKLPA--------                                        318
              *  *: :*..
```

c

```
HU LTA4H   1  MPEIVDTCSLASPASVCRTKHLHLRCSVDFTRRTLTGTAALTVQSQEDNLRSLVLDTKDL    60
MX  5137   1  MARL-DPHSYN-DSTQPETETLDWRARVDFKTQRLHAEVTH--TLKEASAGPLDLDTRDL    56
              * .: *  *    ::  .*: *. *. ***. : * . .:     :* .   * ***:**

HU LTA4H  61  TIEKVV-INGQEVKYALGERQSYKGSPMEISLPIALSKNQEIVIEISFETSPKSSALQWL   119
MX  5137  57  EIRDVIDAAGRPLPYILSPSEPILGSRLRIELPVGLRQ-----FTVRYRTAPHASALQWL   111
               *..*:    *: : * *.  :    ** :.*.**:.* :     : : :.*:*::******

HU LTA4H 120  TPEQTSGKEHPYLFSQCQAIHCRAILPCQDTPSVKLTYTAEVSVPKELVALMSAIRDGET   179
MX  5137 112  TPSQTAGGKHPFLYSQCQAIHARSVVPLQDTPRIRIRYTASLRIPKALKAVMAASFLRRE   171
              **.**:* :**:*:*******.*:::* **** ::: ***.: :** * *:*:*     .

HU LTA4H 180  PDPEDPSRKIYKFIQKVPIPCYLIALVVGALESRQIGPRTLVWSEKEQVEKSAYEFSETE   239
MX  5137 172  E---HGVEAEEHYEMPQPVPPYLLAFAVGSLAPKELGPRSRVWAEPELLEDAAEEFSGVD   228
                  .  .   ::    *:* **:*:.**:*  :::***: **:* * :*.:* *** .:

HU LTA4H 240  SMLKIAEDLGGPYVWGQYDLLVLPPSFPYGGMENPCLTFVTPTLLAGDKSLSNVIAHEIS   299
MX  5137 229  DMLRAAESLFGPYDWERFDLLTMPPSFPYGGMENPRLTFLTPTLITGDKSLVNVVAHELA   288
              .**: **.* *** * ::***.:************ ***:****::***** **:***::

HU LTA4H 300  HSWTGNLVTNKTWDHFWLNEGHTVYLERHICGRLFGEKFRHFNALGGWGELQNSVKTFGE   359
MX  5137 289  HSWTGNLVTNASAEHFWLNEGFTVFAERRILEVLEGPEVSALHGALGRRALDSALQHFRA   348
              ********** : :********.**: **:*    * * :.  ::.  *   *:.::: *

HU LTA4H 360  THPFTKLVVDLTDIDPDVAYSSVPYEKGFALLFYLEQLLGGPEIFLGFLKAYVEKFSYKS   419
MX  5137 349  HPQLTSLRTHLAGVDPDEAFSQIPYEKGYLLLRAMEDAAGRP-AFDEFLRRYLATYRFRA   407
                 :*.* ..*:.:*** *:*.:******: **  :*:  * *  *  **: *: .: :::

HU LTA4H 420  ITTDDWKDFLYSYFKDKVDVLNQVDWNAWLYSPGLPPIKPNYDMTLTNACIALSQRWITA   479
MX  5137 408  LTTEEFVAFAE---KELPGVLTKVDAEAYLHRPGVPPGAPSPRSLRLEAMDALRGKVPTP   464
              :**:::  *     *:  .**.:** :*:*: **:** *.       :*  **  :  *

HU LTA4H 480  KEDDLNSFNATDLKDLSSHQLNEFLAQTLQRAPLPLGHIKRMQEVYNFNAINNSEIRFRW   539
MX  5137 465  ----------EQAKDWTPAEWQLYLES--LPWDIPRDVIQQLDARFSLTESRNSEVLVAW   512
                         : ** :  : : :* .      :* . *::::  :.:.  .***: . *

HU LTA4H 540  LRLCIQSKWEDAIPLALKMATEQGRMKFTRPLFKDLAAFDKSHDQAVRTYQEHKASMHPV   599
MX  5137 513  LVVALRADWEPAVARTETFLGEVGRMKYLKPLYGVLSASHAHRSLARALFKKHGERYHPI   572
              * :.:::.** *:  : .:  * ****: :**:  *:* .  :. *   :::*    **:

HU LTA4H 600  TAMLVGKDLKVD                                                  611
MX  5137 573  ARQGVELILSRA                                                  584
              :   *   *.
```

d

```
HU PTGS2 1      MLARALLLCAVLALSHTANPCCSHPCQNRGVCMSVGFDQYKCDCTRTGFYGENCSTPEFL   60
MX 5217  1      ----------MDEMEGTVQPVA-------GV-------------------STGVPRPAAA   24
                          :  :. *.:* .       **                   . .   *

HU PTGS2 61     TRIKLFL----KPTPNTVH--YIL------------THFKGFWNVVNNIPFLRNAIMSYV   102
MX 5217  25     RRIKPVAKSTLRPFRRTVASRALSGVFSGLNRVIAWHRLPKFLGLLNLIPIRD-ELRAKN   83
                 *** .     :*  .**    :              ::  * .::* **:    : :

HU PTGS2 103    LTSR----SHLIDSPPTYNADYGYKSWE--AFSNLS---------YYTRALPPVPDDCPT   147
MX 5217  84     LYDTTHLPSTQAPEPPTWDPELATRRASDGTYNDLSNPRMGAAGTRFGRNV-PLENAWPE   142
                * .     *    .***:: : . :  .  ::.:**          : * : *: :  *

HU PTGS2 148    PLGVKGKKQLPDSNEIVEKLLLRRKFIPDPQGSNMMFAFFAQHFTHQFFKTDH-KRGPAF   206
MX 5217  143    PEP---ALLEPSPRVISNRLLARQSFVPATS-LNLLAAAWIQFMTHDWFDHGSPKRGGEF   198
                *         *. . * ::** *:.*:*  .  *:: * : *.:**::*. .  ***  *

HU PTGS2 207    TNGL----------------------------------------GHGVDLNHIYGETLARQ   227
MX 5217  199    KVPLDKERGDSWSEDPMRIRRTPEDPTRIPGAKDGPPTYINQHSQWWDASQIYGSNEEET   258
                .  *                                       .:  * .:***..  .

HU PTGS2 228    RKLRLFKDGKMK----YQIIDGEMYPPTVKDTQAEMIYPPQVPEHLRFAVGQEVFGLVPG   283
MX 5217  259    RALRCTDDEDGGQRRGKLILTGEGL---------EAQLPVDPNTHL--QKSGVTHNWWIG   307
                * **  .* .        *: **           *   * :   **    .  ...   *

HU PTGS2 284    LMMYATIWLREHNRVCDVLKQEHPEWGDEQLFQTSRLILIGETIKIVIEDYVQHLS----   339
MX 5217  308    LSLLHTTFAKEHNAIVDRLRLEFPDWNGDRLFHTARLINTALMAKIHTIEWTPAILAHPT   367
                * :  * : :*** : * *: *.*:*..::**:*:***  .   **   ::.  :

HU PTGS2 340    -------------GYHFKLKF----DPELLFN------KQFQYQNRIAAEFNTIYHWHPL   376
MX 5217  368    TETALNTNWWGLVGQRVTRLFGRMSRSELISGIPGSEVNHHGVPFALTEEFVAVYRMHSL   427
                             * :..  *      **: .      ::.     :: ** ::*: * *

HU PTGS2 377    LPDTFQIHDQKYNYQQFIYN--------NSILLEHGI----------------------T   406
MX 5217  428    IPDTMRLHRMRDGVQVREVAMVDLAGPNTLKALEDGLTMVDLCYSFGISHPGALVLHNYP   487
                :***:::*  : . *             .   **.*:

HU PTGS2 407    QFVESFTRQIAGRVAGGRNVPPAVQKVSQASI--DQSRQMKYQSFNEYRKRFMLKPYESF   464
MX 5217  488    AFLRDLHRQD----PDG-----AESRVDLASIDVMRDRERGVPRYNAFRKLMHLQPARSF   538
                 *:..: **      .*     * .:*. ***   :.*:     :* :** : *:* .**

HU PTGS2 465    EELTGEKEMSAELEALYGDIDAVELYPALLVEKPRPDAIFGETMVEVGAPFSLKGLMGNV   524
MX 5217  539    KDITRNEQWARELREVYGHVDRVDLMVGMLAEDPPRGFGFSDTAFRVFILMASRRLA---   595
                :::* ::: : **. :**.:* *:*  .:*.*.*  .  *.:* ..*   :: : *

HU PTGS2 525    ICSPAYWKPS---TFGGEVGFQIINTASIQSLICNNVKGCPFTSFSVPDPELIKTVTINA   581
MX 5217  596    --SDRFFTNDQNVNLYTQPGMAWLNENTMASVLLRHYPGL--------APALRQT-----   640
                  *  ::. .   .:  : *:  :*  :: *:: .:  *          * * :*

HU PTGS2 582    SSSRSGLDDINPTVLLKERS--TEL--                                  604
MX 5217  641    ---RNAFAPWEPVSMVATEPPAGSLLH                                  664
                   *..:   :*. ::  .    .*
```

e

```
HU TBXAS1   1   MEALGFLKLEVNGPMVTVALSVALLALLKWYSTSAFSRLEKLGLRHPKPSPFIGNLTFFR   60
MX   0683   1   --------------MVRSTCASGSSPL-----QDPFMSQVRL-PPGPQGHFIAGNLVEFS   40
MX   2304   1   ----------------MSIH------------PAGASTAAPL-PPLSPGAPLIGHLRALR   31
                                                         *         : *:*  :

HU TBXAS1  61   QGFWESQMELRKLYGPLCGYYLGRRMFIVISEPDMIKQVLVENFSNFTNRMASGL--E-F   117
MX   0683  41   EDPLGFLTRCAREYGDVVRLGKR---NFLLNHPDLIERVLVNGDGNFVKLAGVGQGKRHK   97
MX   2304  32   KDPLHFLQAQAREYGDVVRLPMGPADLVLVAHPDGVRHVLQDHARNYSKQSRGF--RVLQ   89
                :.         : ** :          .:: .** :.:** :   *: :

HU TBXAS1 118   KSVADSVLFLRDKRWEEVRGALMSAFSPEKLNEMVPLISQACDLLLAHLKRYAESGDAFD   177
MX   0683  98   GGFPEAMMNSEGEDWLRKRRLVQPAFHRKHVAACGDTVVALTE----TMLQTWRPGDARD   153
MX   2304  90   ELLGHGLLTSDGDHWLRQRRLAQPAFHRQRVAGFTRTMVDAAADLAATMEARADTGAAFN   149
                  . ..::   .. * . *     **  :::      :          :      * * :

HU TBXAS1 178   IQRCYCNYTTDVVASVAFGTPVDSWQAPEDPFVKHCKRFFEFCIPRPILVLLLSFPSIMV   237
MX   0683 154   VHADVSALALDIVSRFLFHTPIDDEARHVADAVDA----VMRHTDSPLR------PPIWV   203
MX   2304 150   VAEDFTRLTLRIASSTLFGADVSSATHDIATVMSRLQVFVYKRLTQPVP------LSLRL   203
                :       :  :.:   * : :..        :.     .      *:         : :

HU TBXAS1 238   PLARILPNKNRDELNGFFNKLIRNVIALRDQQAAEERRRDFLQMVLDARHSASPMGVQDF   297
MX   0683 204   PTPT---NLRLRRALGRLNTLLATLVRRYRE--QPESRTDLLALLLSA-----PVPLS--   251
MX   2304 204   PLPA---HRQFERDVGSLNRVVHGIIAKRRR--ESGEHHDLLQMMMEAHDDDTGERMS--   256
                *      : .  .  * :* ::  ::    .      : *:* :::.*        :.

HU TBXAS1 298   DIVRDVFSSTGCKPNPSRQHQPSPMARPLTVDEIVGQAFIFLIAGYEIITNTLSFATYLL   357
MX   0683 252   ------------------------------ENQLRDELATMIMSGHETTADALVWAWYLL   281
MX   2304 257   ------------------------------DSQLRDEVLTLLLAGHETTASALAWTIMLL   286
                                               .:: .:   ::::*:*  :.:* ::  **

HU TBXAS1 358   ATNPDCQEKLLREVDVFKEKHMAPEFCSLEEGLPYLDMVIAETLRMYPPAFRFTREAAQD   417
MX   0683 282   AQHPEAEARLVAELETVLGGRLP-G-AEDLPRLRYTEAVVKEAMRLYSPAWITSREALRD   339
MX   2304 287   SQHPGVRRDMESELARELGGRNP-T-HEDLPRLELTHRVVDESLRLYPPAWALSRIATKE   344
                : :*  .  :  *:      :      .    *   . *: *::*:* **:  :* * ::

HU TBXAS1 418   CEVLGQRIPAGAVLEMAVGALHHDPEHWPSPETFNPERFTAEARQQHRPFTYLPFGAGPR   477
MX   0683 340   CELGGFHVPAGTMLAVSQWVTHRDARYFDAPESFRPDRWLSEDAQRMHRYVYFPFGGGPR   399
MX   2304 345   DLVGGFRIPKGAHLLIAPWVTHRHPSIWDNPEGFDPDRFLPEREQARPRFAWFPFGGGPR   404
                  : * ::* *: * ::  . *:.   :  ** * *:*:  *  *    :.::***.***

HU TBXAS1 478   SCLGVRLGLLEVKLTLLHVLHKFRFQACPETQVPLQLESKSALGPKNGVYIKIVSR----   533
MX   0683 400   FCIGSALAMMETVLITACVARRFRLELAPGCVVR--PRPALALQPL-GVWLIPRHRSHTT   456
MX   2304 405   QCIGNQFALMELVLVLATLLQRVRLNLTPGQVIH--PTPAITLRPRPGVWVTASRP----   458
                 *:*  :.::*  *    : ::.*::  *   :        :* *  **::

HU TBXAS1 534   -----------                                                  533
MX   0683 457   QEGEVRHAAGA                                                  467
MX   2304 459   -----------                                                  458
```

f

```
HU PTGS2   1   ----MPNYKLTYFNMRGRAEIIRYIFAYLDIQYEDHRIEQADWPE-----------IKST   45
MX  5217   1   MNAQSSLPRLSYFTGRGVAEKIRLLLAESGTEYEDIDLGAYDVQAKVKTPAFEAIKAAGM   60
                       :*:**. ** ** ** ::*  . :***  :   *                .

HU PTGS2  46   LPFGKIPIL-EVDGLTLHQSLAIARYLTKNTDLAGNTEMEQCHVDAIVDTLDDFMSCFPW   104
MX  5217  61   LAFDKVPLWEEPDGFRVVQSLAILRHVARTRGLYGKDARETTACDMIIDGVEEVTARARS   120
               * *.*:*:  * **: : ***** *::::. .* *:   *    * *:* :::. :

HU PTGS2 105   A-EKKQDVKEQMFNELLTYNAP----HLMQDLDTYLGGREWLIGNSVTWADFYWEICSTT   159
MX  5217 121   LTSLTPDQLSEQLPIILGEELPQWLAHFERLLKSNGSGDGFFVGPSVTVADTS-------   173
                 . . *  .: :  :*  : *    *: : *.:  .*  :::* *** **

HU PTGS2 160   LLVFKPDLLDN---------HPRLVTLRKKVQAIPAVANW---IKRRPQTKL---   199
MX  5217 174   VFGFLELLVDNGLQDLLESTYPGLFGFFERMKQRPNLARHLASSKRHPAVQLLNG   228
               :: *   *:**         :* *. : ::::  *  :*.     **:*  .:*
```

Supplementary Figure 3. Amino acid sequences alignment of the enzymes in *Myxococcus xanthus*. **(a)** Candidates of lipoxygenases (LOXs). The GenBank accession numbers of human 12-LOX, *MX_1744*, and *MX_1745* are P18054, Q1DBH9, Q1DBH8, respectively. **(b)** Candidate of epoxide hydrolase (EH). The GenBank accession numbers of human bifunctional EH2 and *MX_1644* are P34913 and Q1DBS7, respectively. **(c)** Candidate of EH. The GenBank accession numbers of human leukotriene A_4 hydrolase and *MX_5137* are P09960 and Q1D232, respectively. **(d)** Candidate of cyclooxygenase (COX). The GenBank accession numbers of human COX and *MX_5217* are P35354 and Q1D1V4, respectively. **(e)** Candidate of TXA synthase. The GenBank accession numbers of human thromboxane A synthase, *MX_0683*, and *MX_2304* are P24557, Q1DEH2, and Q1D9Z9, respectively. **(f)** Candidate of PGD synthase. The GenBank accession numbers of PDG synthases as follows; Human PGD synthase *MX_3623* are O60760 and Q1D6B3, respectively. The boxes are shown conserved major residues in enzymes. Red color means metal binding residues, yellow color means positional residues, orange color means stereo residues, green color means substrate binding residues, and blue color means active site residues.

a

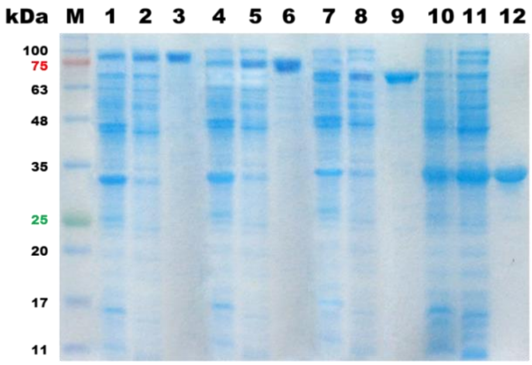

b

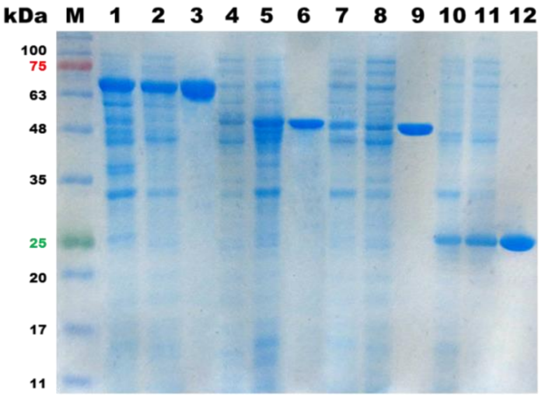

Supplementary Figure 4. SDS-PAGE analysis of enzymes in *Myxococcus xanthus*. **(a)** Expression of the candidate biosynthetic enzymes 2 LOXs (*MXAN_1745* and *MXAN_1744*), 1 COX (*MXAN_5217*), and 1 EH (*MXAN_1644*) in *M. xanthus*. M; marker, lane 1; *MXAN_1745* pellet, 2; *MXAN_1745* crude, 3; *MXAN_1745* purified emzyme, 4; *MXAN_1744* pellet, 5; *MXAN_1744* crude, 6; *MXAN_1744* purified enzyme, 7; *MXAN_5217* pellet, 8; *MXAN_5217* crude, 9; *MXAN_5217* purified enzyme, 10; *MXAN_1644* pellet, 11; *MXAN_1644* crude, 12; *MXAN_1644* purified enzyme. **(b)** Expression of the candidate biosynthetic enzymes 1 EH (*MXAN_5137*), 2 thromboxane synthase (*MXAN_0683*, *MXAN_2304*), and 1 PGD synthase (*MXAN_3623*) in *M. xanthus*. M; marker, lane 1; *MXAN_5137* pellet, 2; *MXAN_5137* crude, 3; *MXAN_5137* purified emzyme, 4; *MXAN_0683* pellet, 5; *MXAN_0683* crude, 6; *MXAN_0683* purified enzyme, 7; *MXAN_2304* pellet, 8; *MXAN_2304* crude, 9; *MXAN_2304* purified enzyme, 10; *MXAN_3623* pellet, 11; *MXAN_3623* crude, 12; *MXAN_3623* purified enzyme.

a

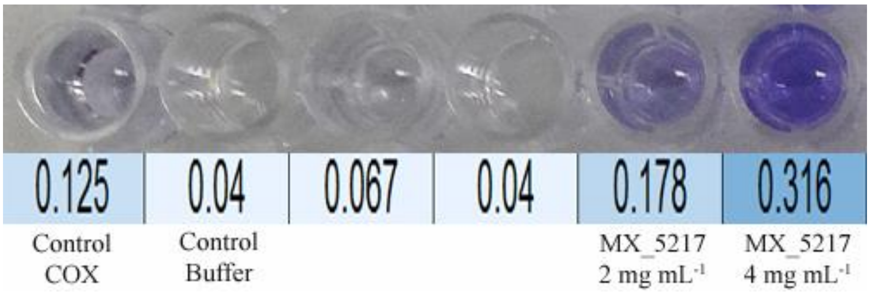
0.125
0.04
0.067
0.04
0.178
0.316
Control COX
Control Buffer
MX_5217 2 mg mL-1
MX_5217 4 mg mL-1

b

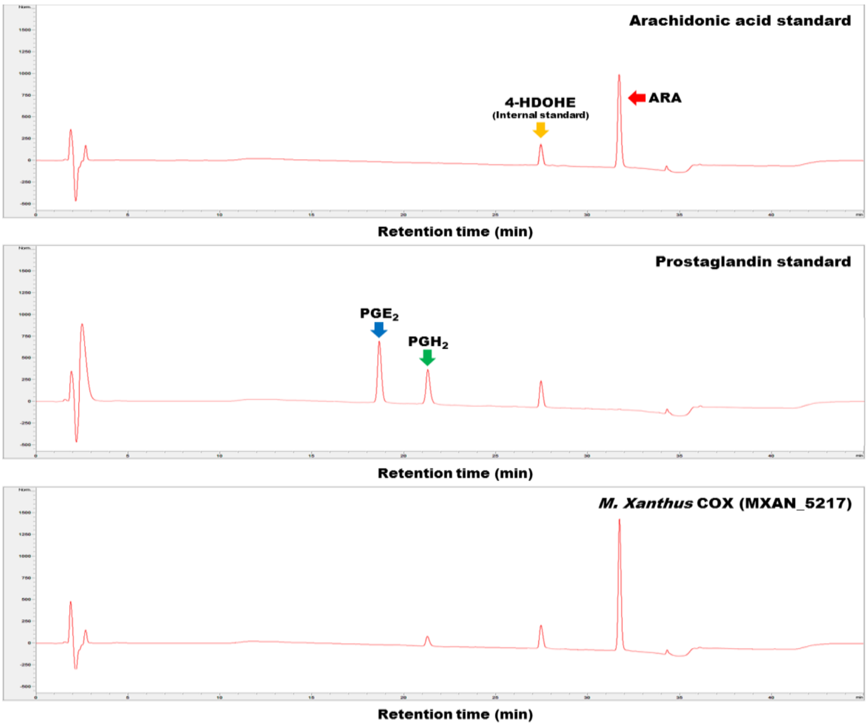
Arachidonic acid standard
4-HDOHE
(Internal standard)
ARA
Retention time (min)
Prostaglandin standard
PGE2
PGH2
Retention time (min)
M. Xanthus COX (MXAN_5217)
Retention time (min)

c

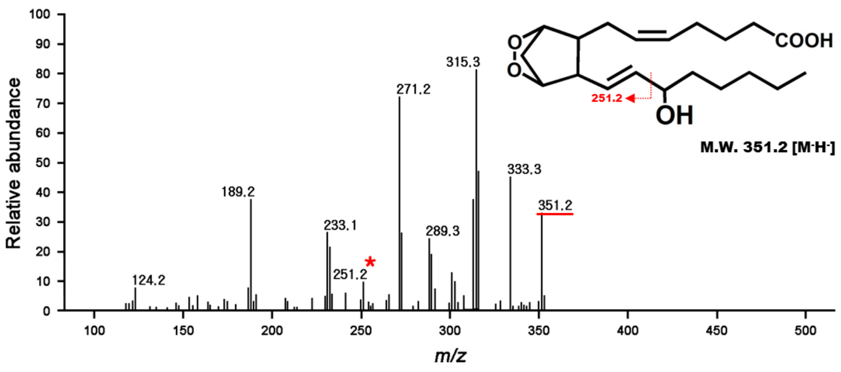
Relative abundance
m/z
124.2
189.2
233.1
251.2
271.2
289.3
315.3
333.3
351.2
COOH
OH
251.2
M.W. 351.2 [M-H-]

Supplementary Figure 5. Activity and identification of a metabolite produced from arachidonic acid by cyclooxygenase from *Myxococcus xanthus*. The determination using HPLC and MS/MS analyse. **(a)** Activity of COX from *M. xanthus*. COX (*MXAN_5217*) activity was measured using a COX activity assay kit. **(b)** HPLC analysis for a metabolite produced from ARA by COX from *M. xanthus* with the standards PGE_2 and PGH_2. **(c)** MS/MS analysis for PGH_2 produced from ARA by COX from *M. xanthus*. The metabolite was identified as PGH_2. The red asterisks and the number with read underline indicates molecular masses of key fragments and total molecular mass of the compound, respectively.

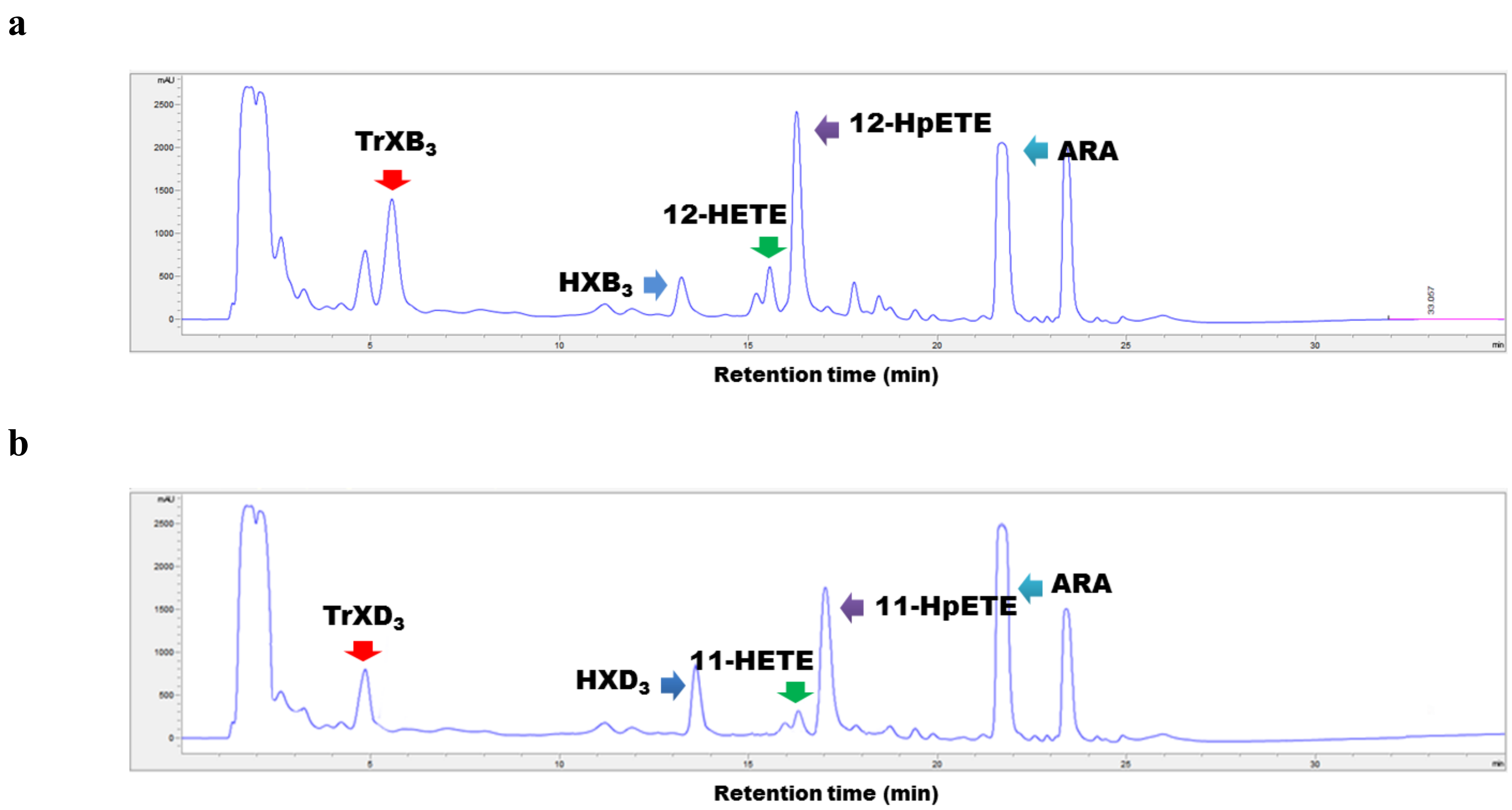

Supplementary Figure 6. HPLC analysis of metabolites from recombinant *Escherichia coli*. The metabolites produced from (ARA) by the cultivation of recombinant *E. coli* expressing 12-LOX or 11-LOX and epoxide hydrolase (EH) from *M. xanthus*. **(a)** Metabolites produced by cells expressing 12-LOX and EH. **(b)** Metabolites produced by cells expressing 11-LOX and EH. The reactions were performed at 30°C in 50 mM EPPS (pH 8.5) containing 1 mM ARA and 7.2 g L^{-1} recombinant cells for 120 min.

a

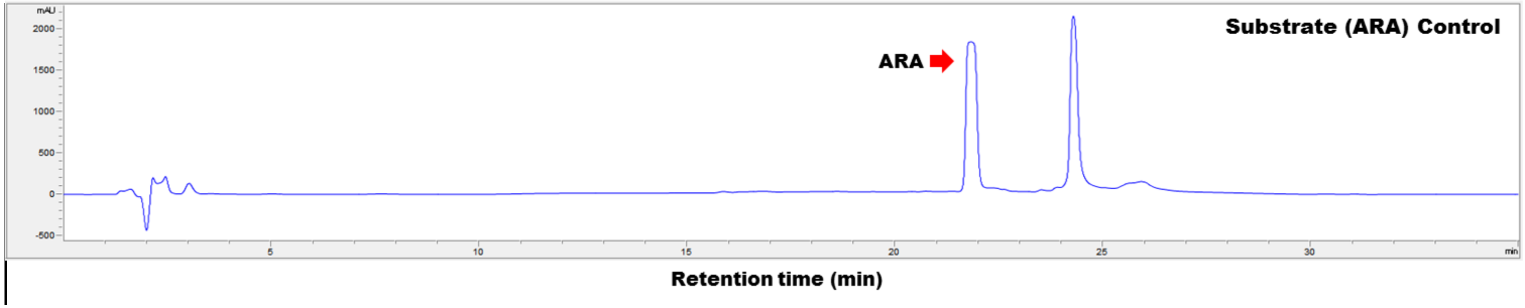

b

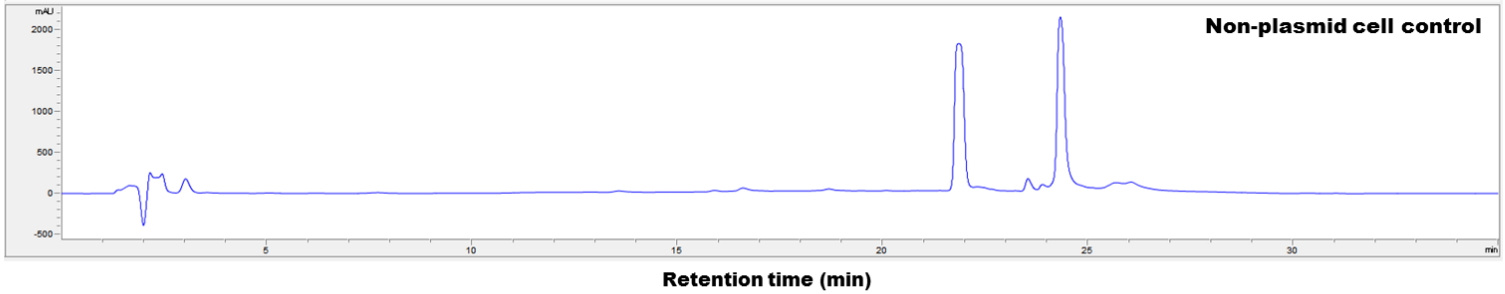

Supplementary Figure 7. Determination of non-enzymatic products by *Escherichia coli* without plasmid. (a) HPLC analysis after incubation of only ARA for 120 min. The reaction was performed at 30°C in 50 mM EPPS (pH 8.5) containing 1 mM ARA for 120 min. **(b)** HPLC analysis after incubation of *E. coli* without plasmid containing ARA for 120 min. The reaction was performed at 30°C in 50 mM EPPS (pH 8.5) containing 1 mM ARA and 7.2 g L^{-1} cells for 120 min.

a

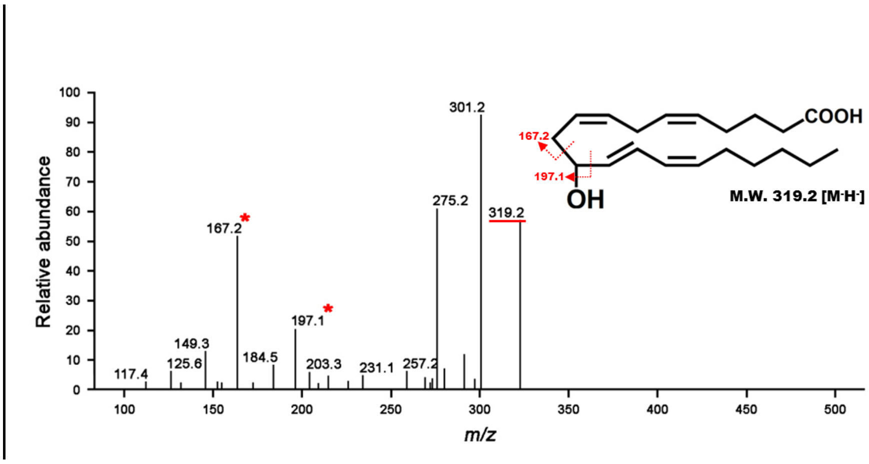

11-HETE

b

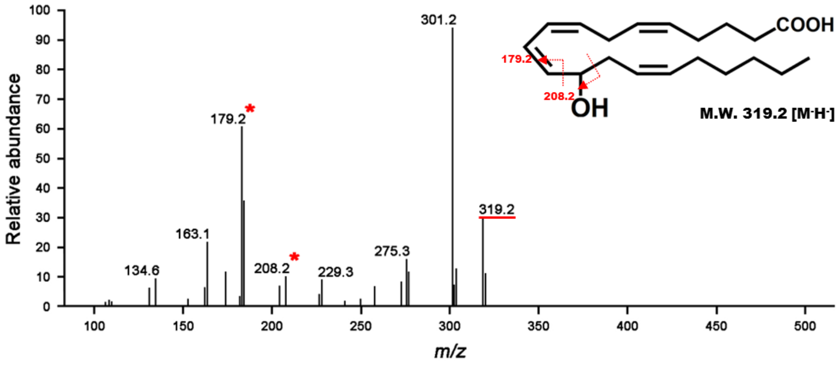

12-HETE

c

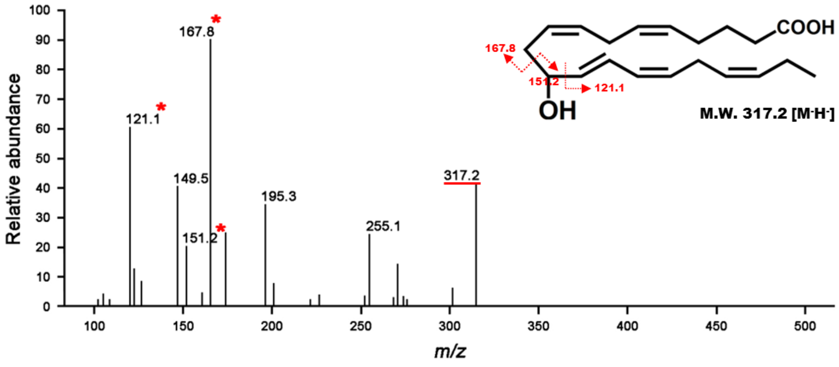

11-HEPE

d

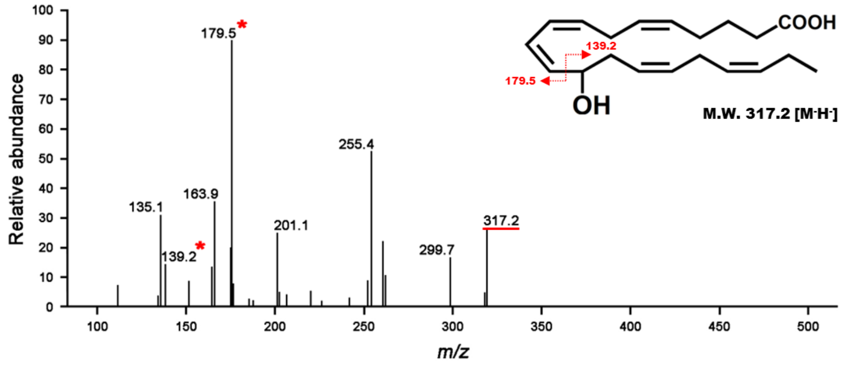

12-HEPE

e

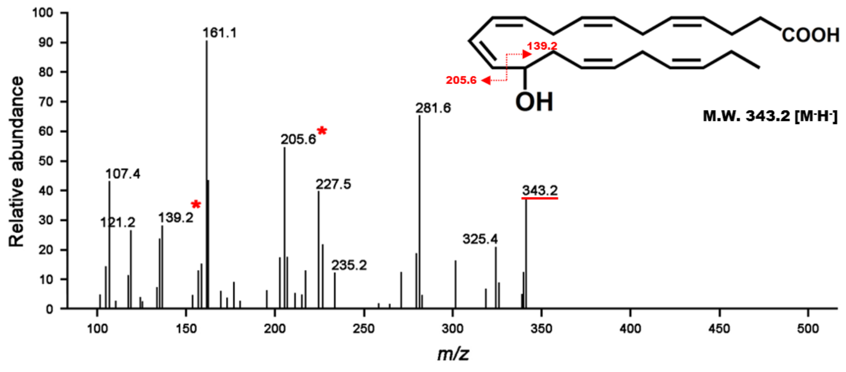

14-HDoHE

Supplementary Figure 8. LC-MS/MS analysis of hydroxy fatty acids. The hydroxy fatty acids (HFAs) were produced from polyunsaturated fatty acids (PUFAs) by 12-LOX or 11-LOX from *M. xanthus*. **(a)** 11-Hydroxyeicosatetraenoic acid (11-HETE). **(b)** 12-HETE. **(c)** 11-Hydroxyeicosapentaenoic acid (11-HEPE). **(d)** 12-HEPE. **(e)** 14-Hydroxydocosahexaenoic acid (14-HDoHE). The red asterisks and the number with read underline indicates molecular masses of key fragments and total molecular mass of the compound, respectively.

a

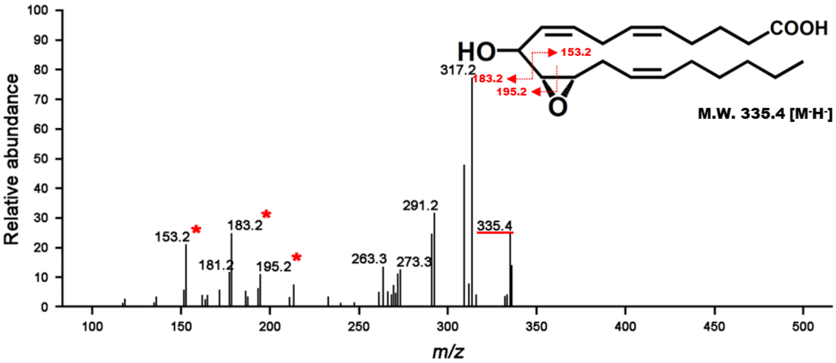

HXB$_3$

b

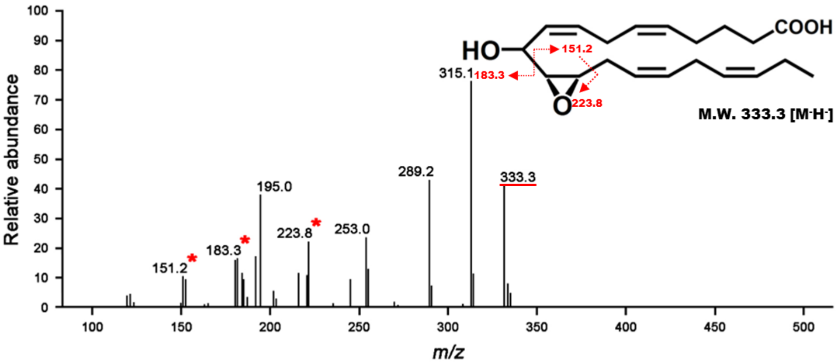

HXB$_4$

c

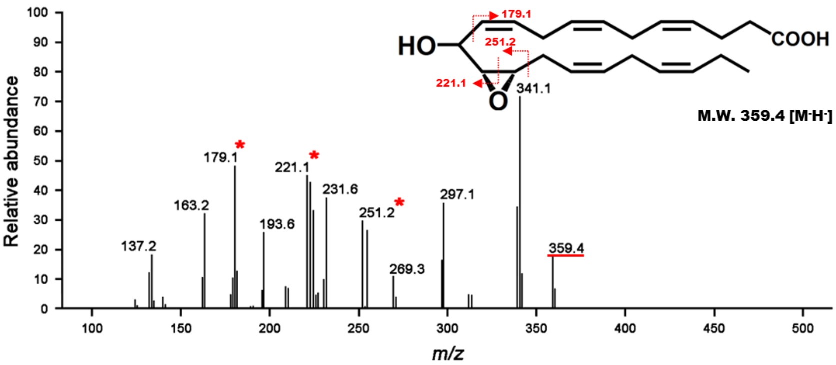

HXB$_5$

d

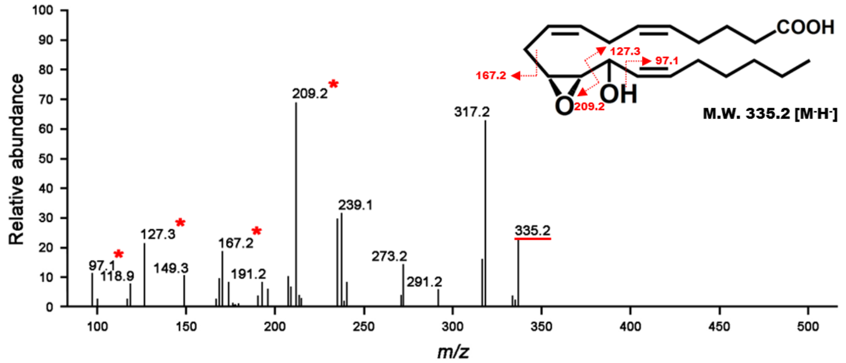

HXD$_3$

e

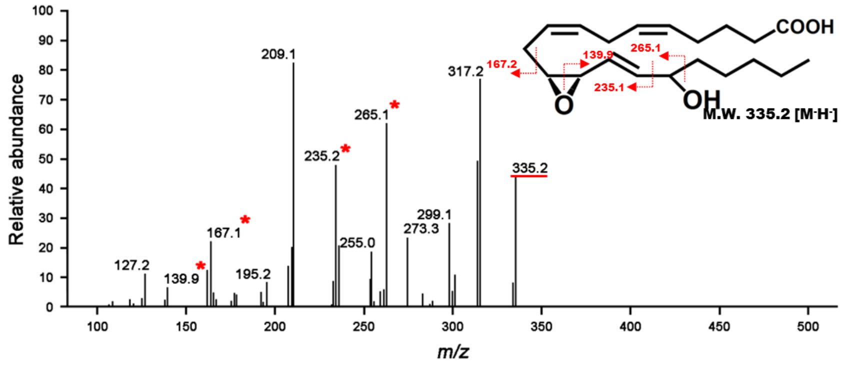

HXE$_3$

Supplementary Figure 9. LC-MS/MS analysis of hepoxilins. Hepoxilins (HXs) were produced from PUFAs by 12-LOX or 11-LOX from *M. xanthus*. **(a)** HXB$_3$. **(b)** HXB$_4$. **(c)** HXB$_5$. **(d)** HXD$_3$. **(e)** HXE$_3$. The red asterisks and the number with read underline indicates molecular masses of key fragments and total molecular mass of the compound, respectively.

a

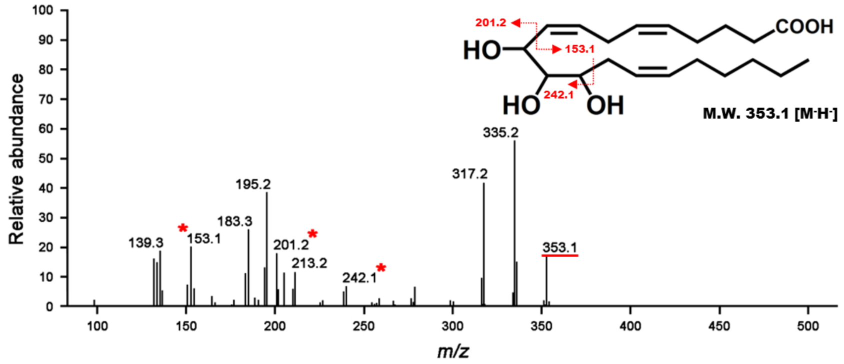

TrXB$_3$

b

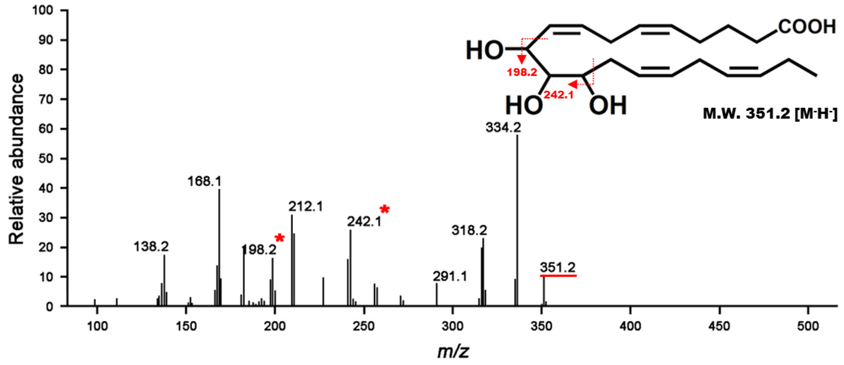

TrXB$_4$

c

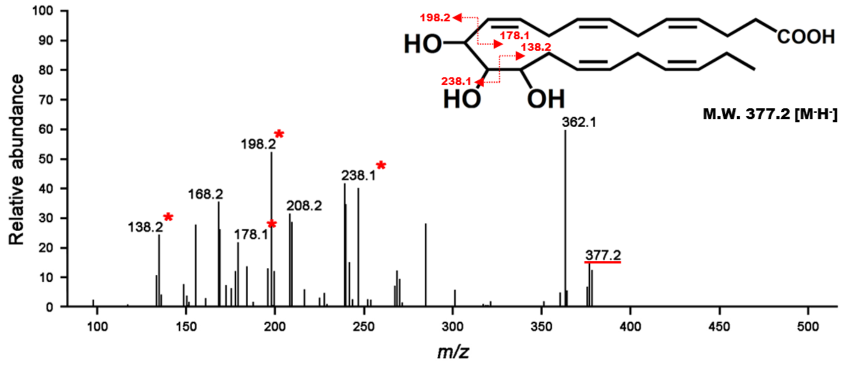

TrXB$_5$

d

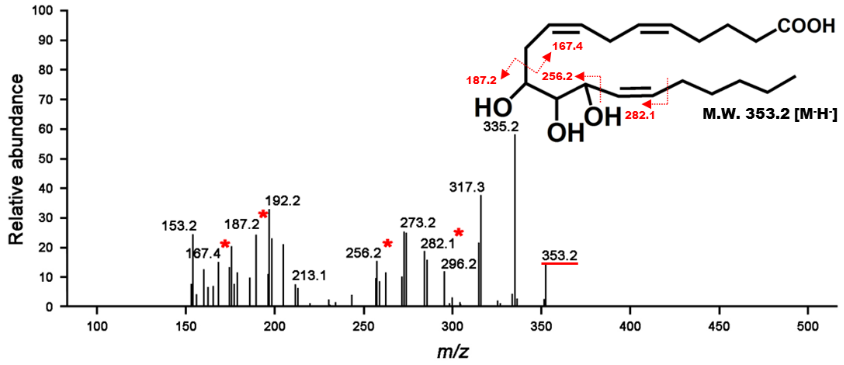

TrXD_3

e

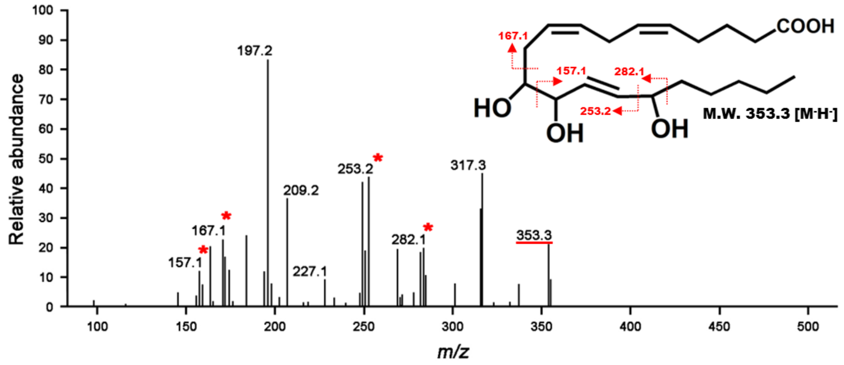

TrXE_3

Supplementary Figure 10. LC-MS/MS analysis of trioxilins. Trioxilins (TrXs) were produced from PUFAs by 12-LOX or 11-LOX and (EH) from *M. xanthus*. **(a)** TrXB_3. **(b)** TrXB_4. **(c)** TrXB_5. **(d)** TrXD_3. **(e)** TrXE_3. The red asterisks and the number with read underline indicates molecular masses of key fragments and total molecular mass of the compound, respectively.

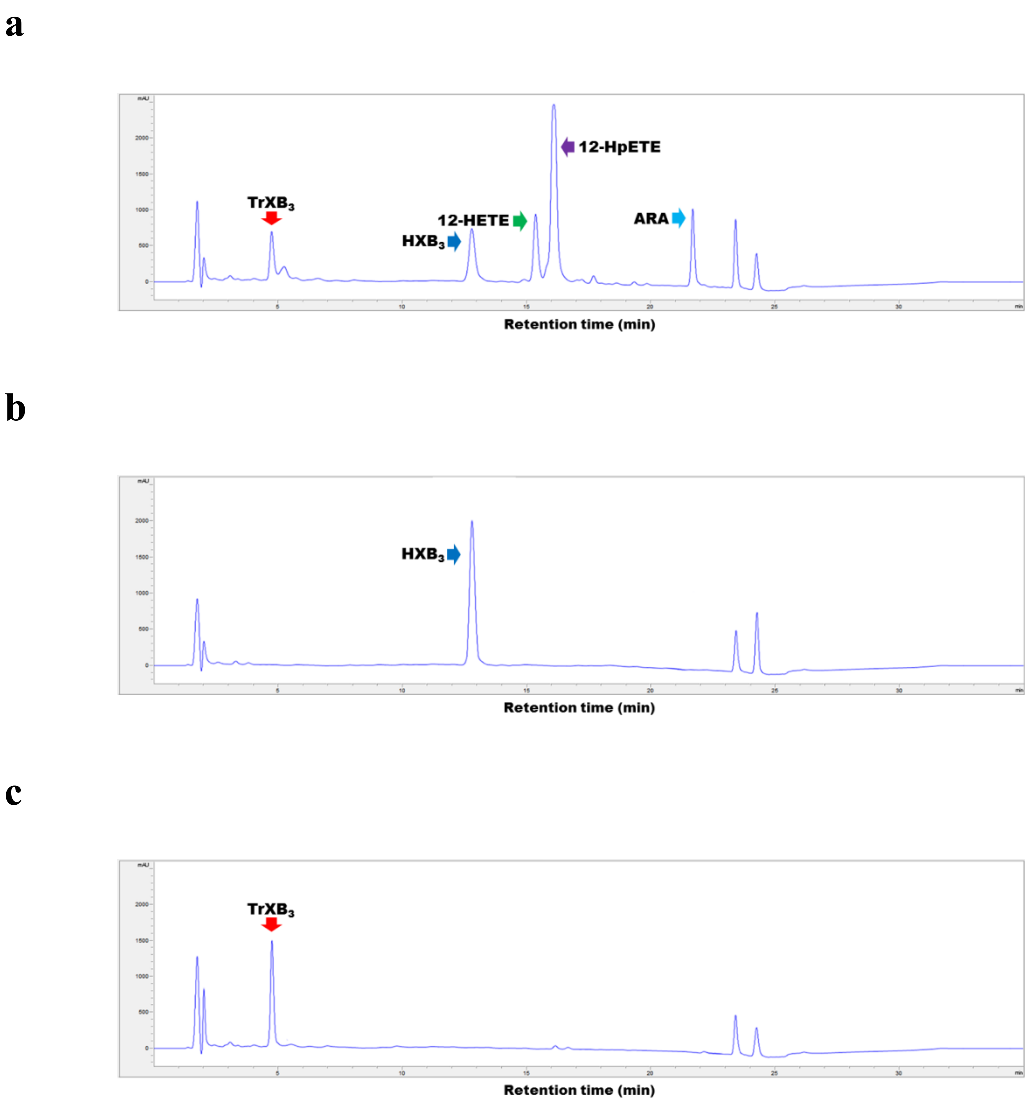

Supplementary Figure 11. Purification of hepoxilin B_3 and trioxilin B_3 using Prep-LC. (a) Reaction products from ARA by cells expressing 12-LOX and EH from *M. xanthus*. **(b)** Purified hepoxilin B3 (HXB_3). **(c)** Purified trioxilin B3 ($TrXB_3$).

a

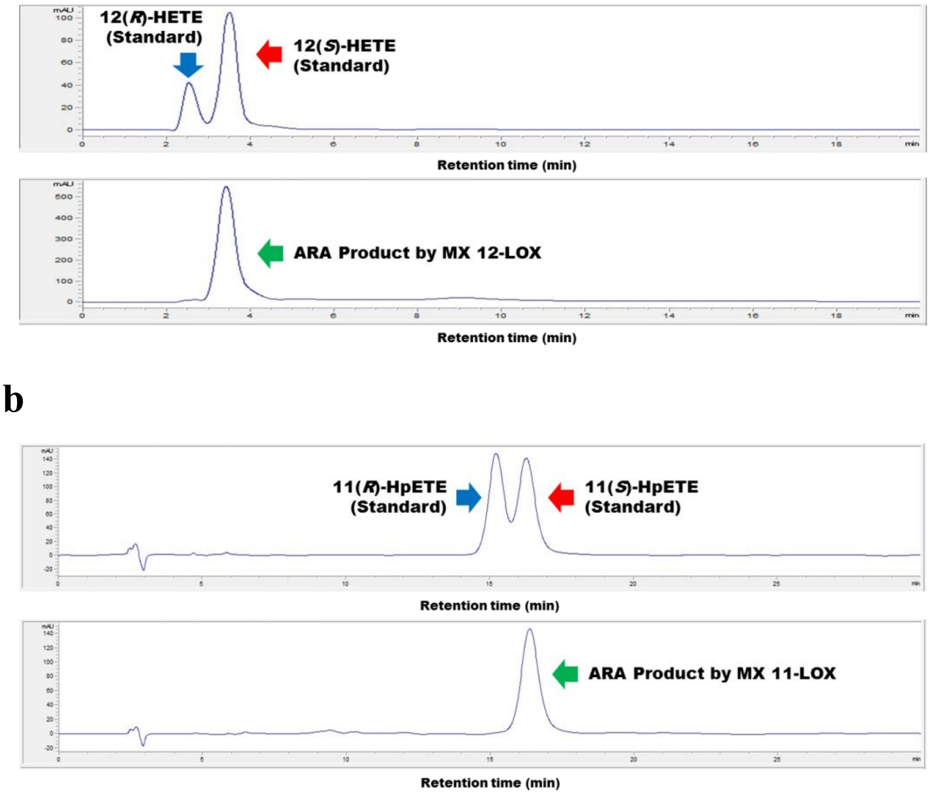

Supplementary Figure 12. Stereospecificity of metabolites of recombinant *Escherichia coli.* The reactions were performed with ARA. **(a)** The product of 12-LOX from *M. xanthus* was identified as 12*S*-HETE by comparing with the standards 12*S*-HETE and 12*R*-HETE. **(b)** The product of 11-LOX from *M. xanthus* was identified as 11*S*-HETE by comparing with the standards 11*S*-HETE and 11*R*-HETE.

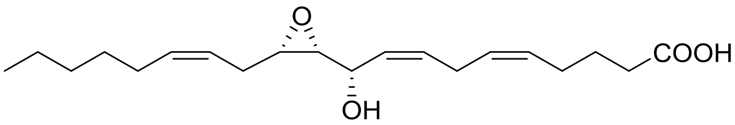

(*S*,5*Z*,8*Z*)-10-hydroxy-10-((2*R*,3*S*)-3-((*Z*)-oct-2-en-1-yl)oxiran-2-yl)deca-5,8-dienoic acid

Supplementary Figure 13. Structure of hepoxilin B_3.

a

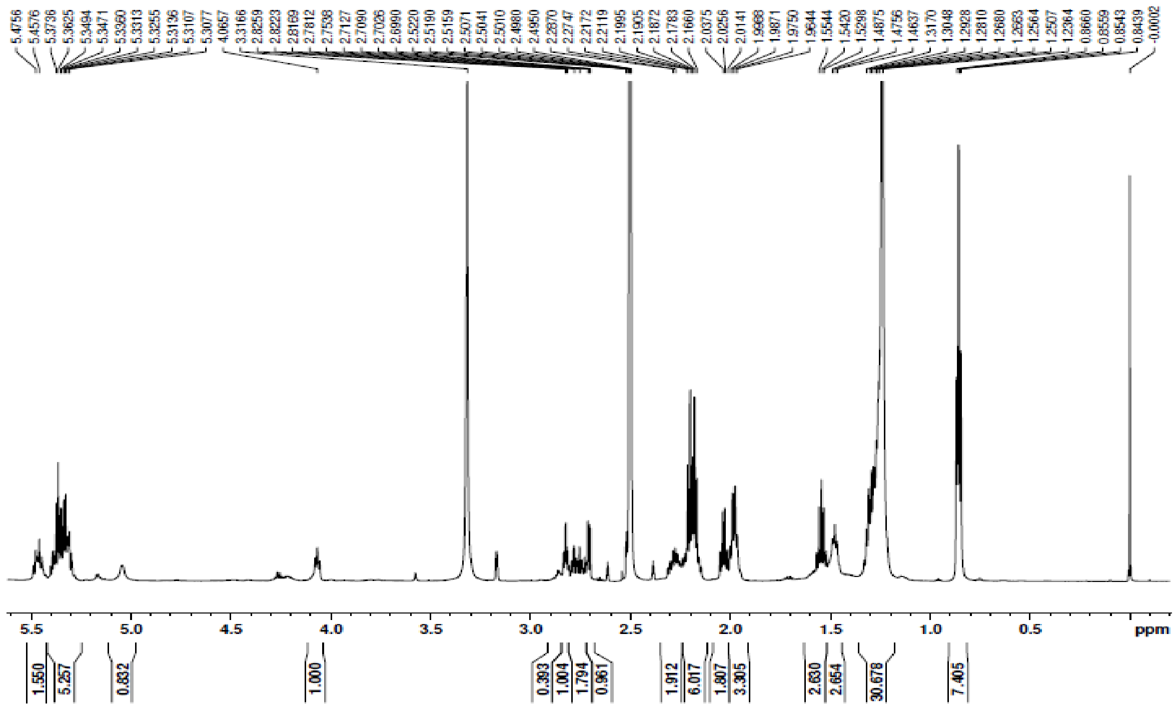

b

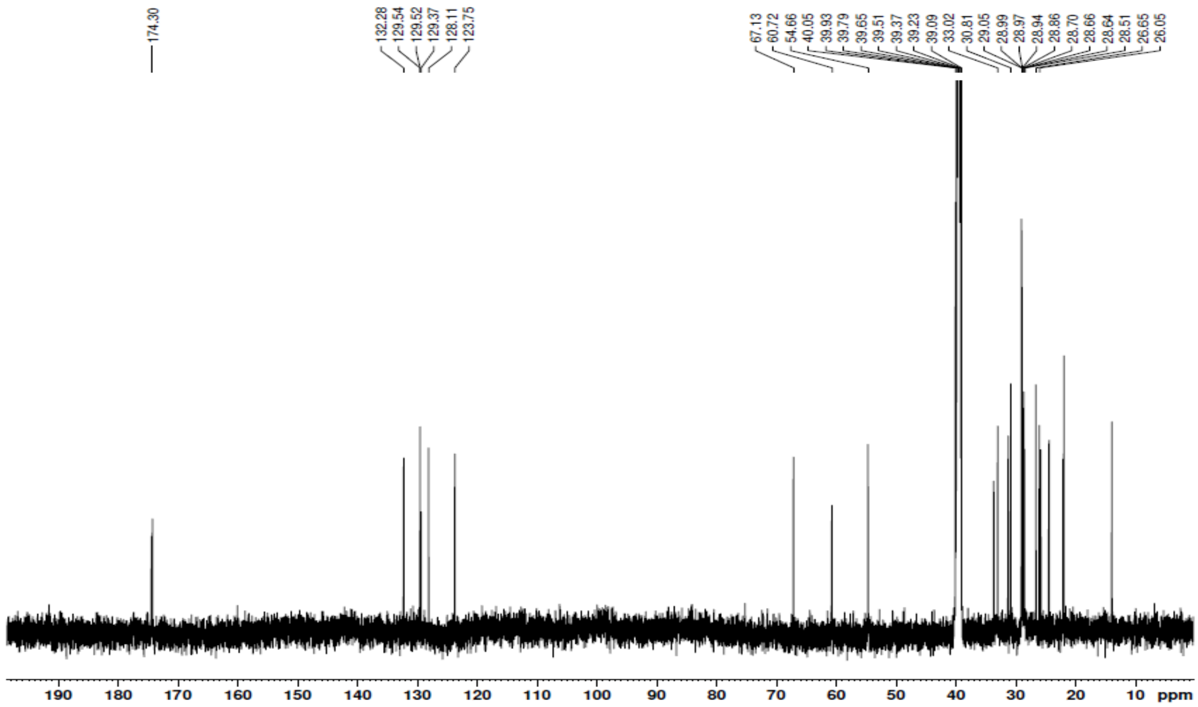

Supplementary Figure 14. 1D NMR data of hepoxilin B_3. (a) 1H NMR peak of hepoxilin B3 (HXB_3). **(b)** 13C NMR peak of HXB_3.

a

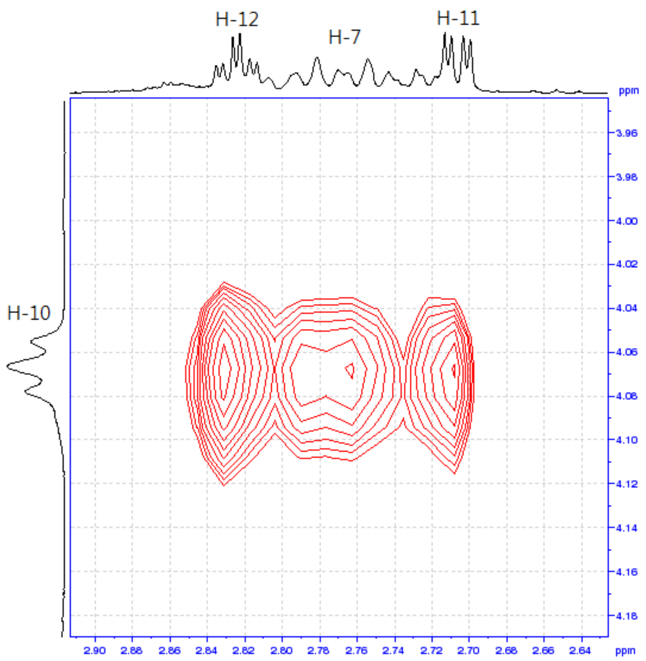

b

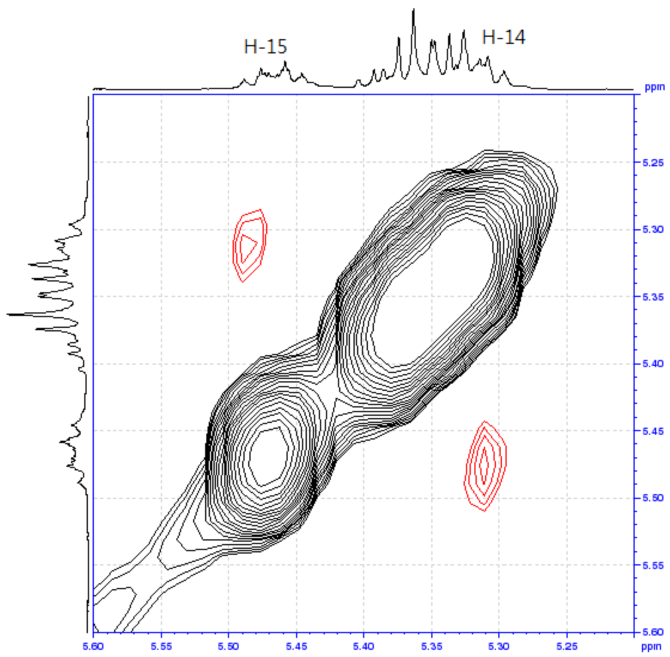

Supplementary Figure 15. ROESY NMR of hepoxilin B_3. (a) ROE correlation of H-10, H-11, and H-12 of HXB_3 at ROESY. **(b)** ROE correlation of H-14 and H-15 of HXB_3 at ROESY.

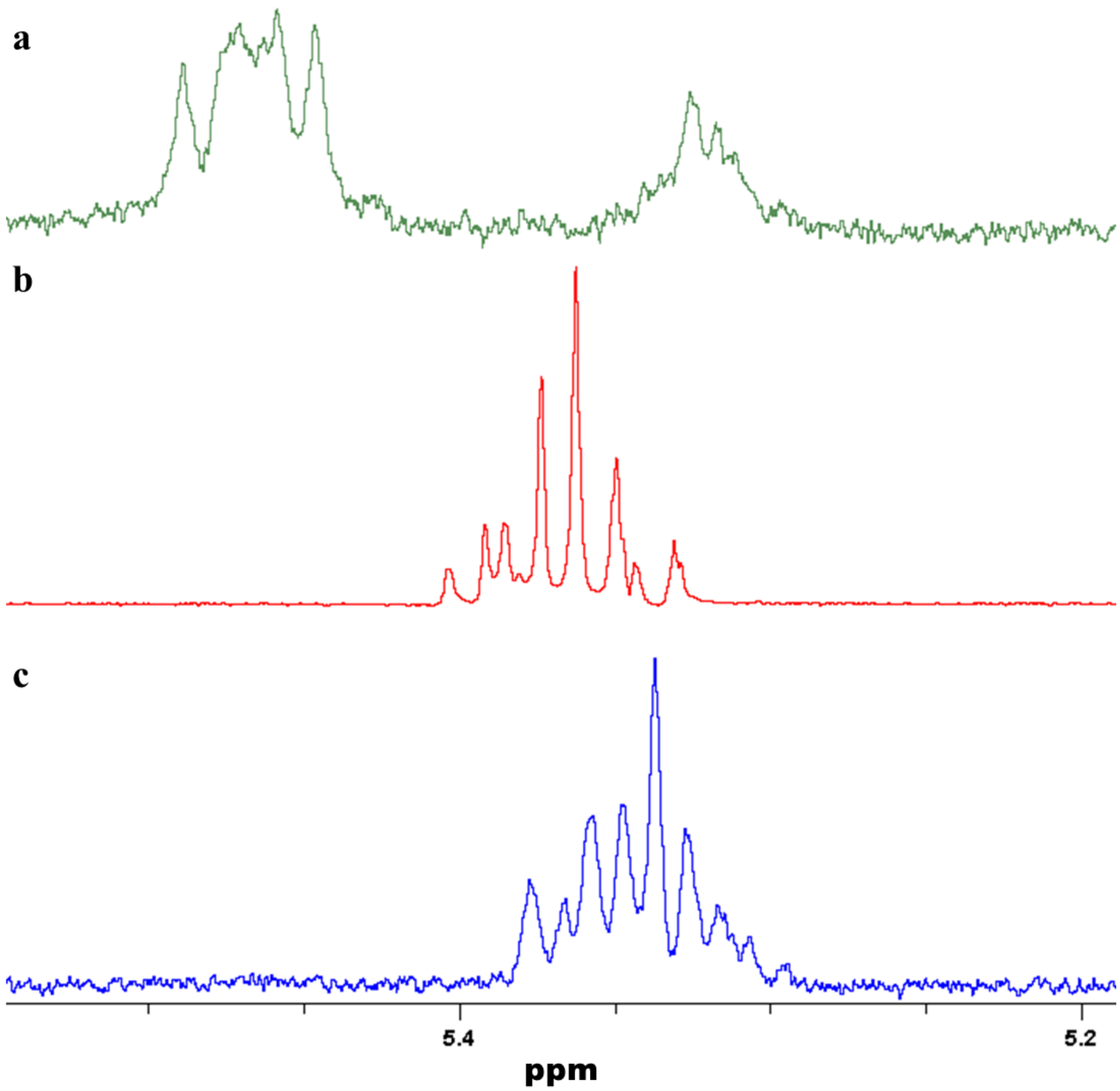

Supplementary Figure 16. H-5, H-6, H-8, H-9, H-14, and H-15 of hepoxilin B$_3$. The peaks were confirmed with the selective TOCSY (mixing time = 40 ms). **(a)** H-15,14 irradiation on H-17. **(b)** H-9,8 irradiation on H-10. **(c)** H-5,6 irradiation on H-3.

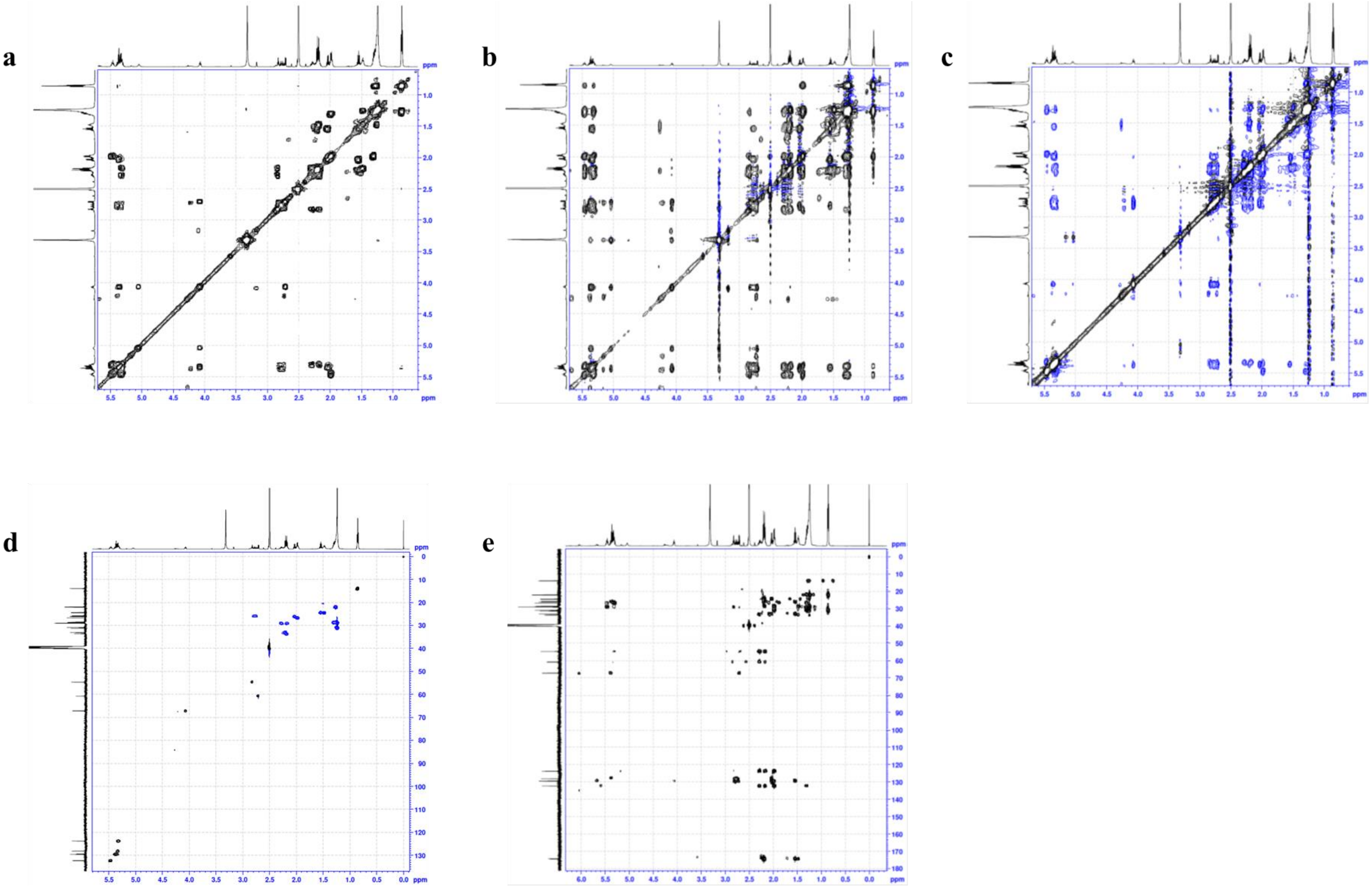

Supplementary Figure 17. 2D NMR of hepoxilin B$_3$. (a) COSY. **(b)** TOCSY. **(c)** ROESY. **(d)** HSQC. **(e)** HMBC.

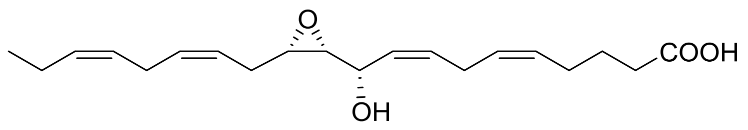

(*S*,5*Z*,8*Z*)-10-hydroxy-10-((2*R*,3*S*)-3-((2*Z*,5*Z*)-octa-2,5-dien-1-yl)oxiran-2-yl)deca-5,8-dienoic acid

Supplementary Figure 18. Structure of hepoxilin B_4.

a

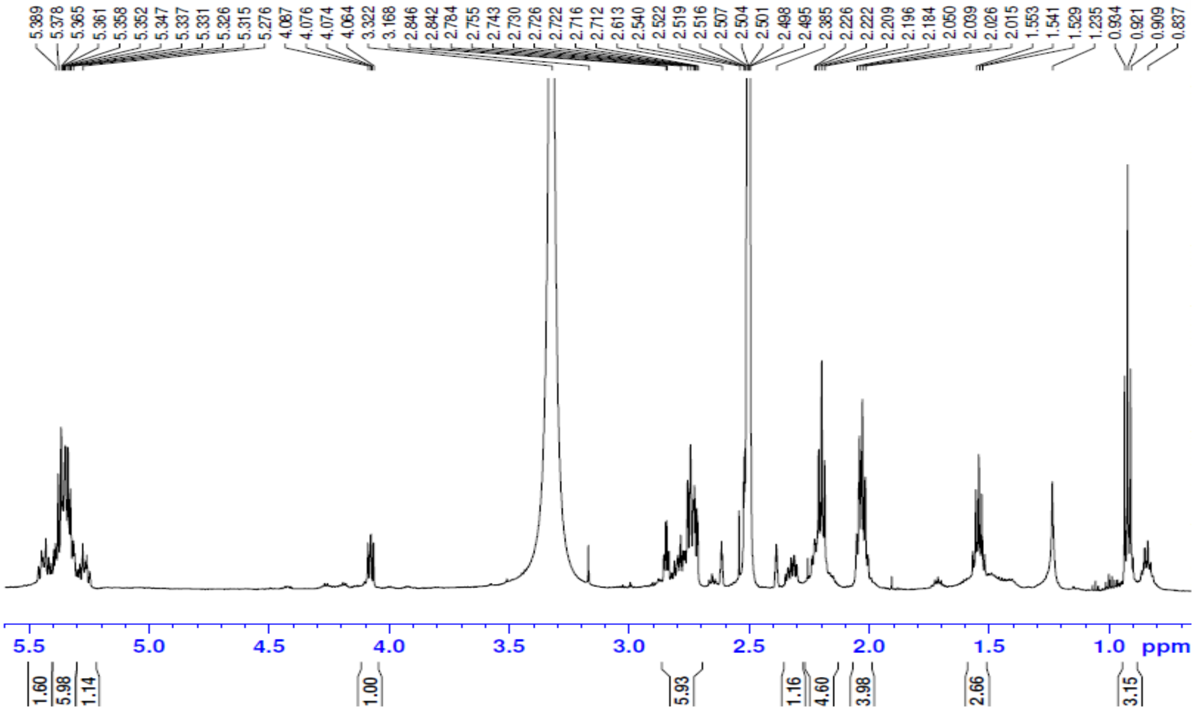

b

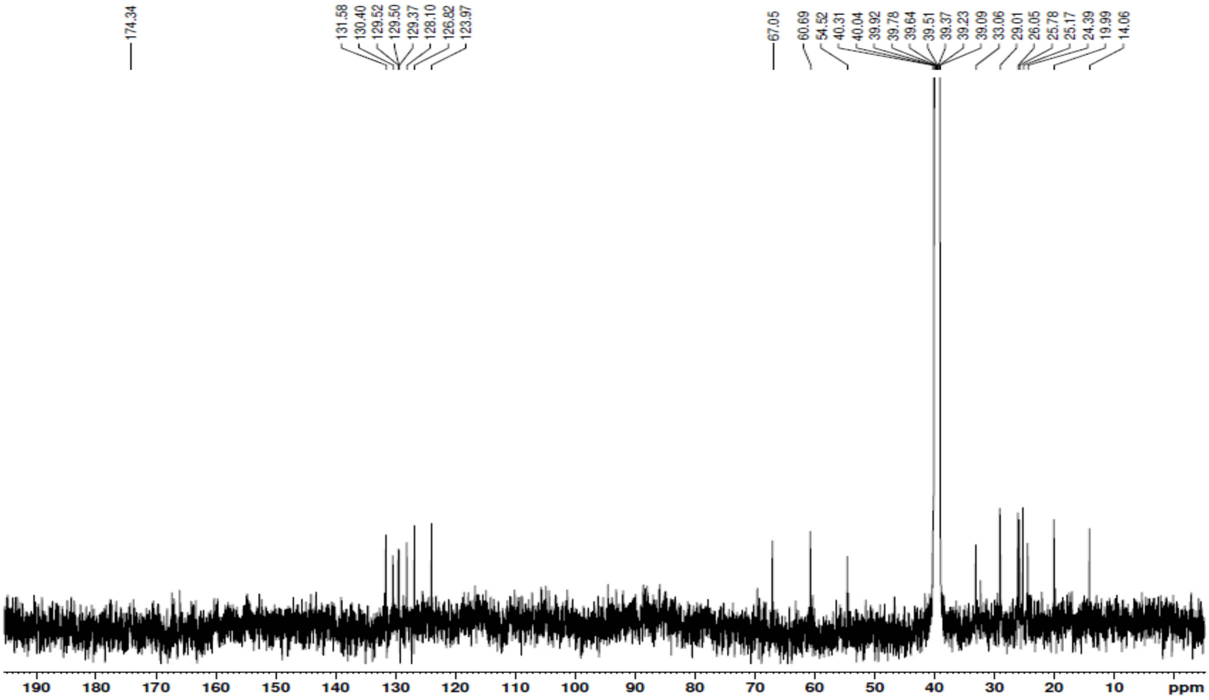

Supplementary Figure 19. 1D NMR data of hepoxilin B_4. (a) 1H NMR peak of HXB_4. **(b)** 13C NMR peak of HXB_4.

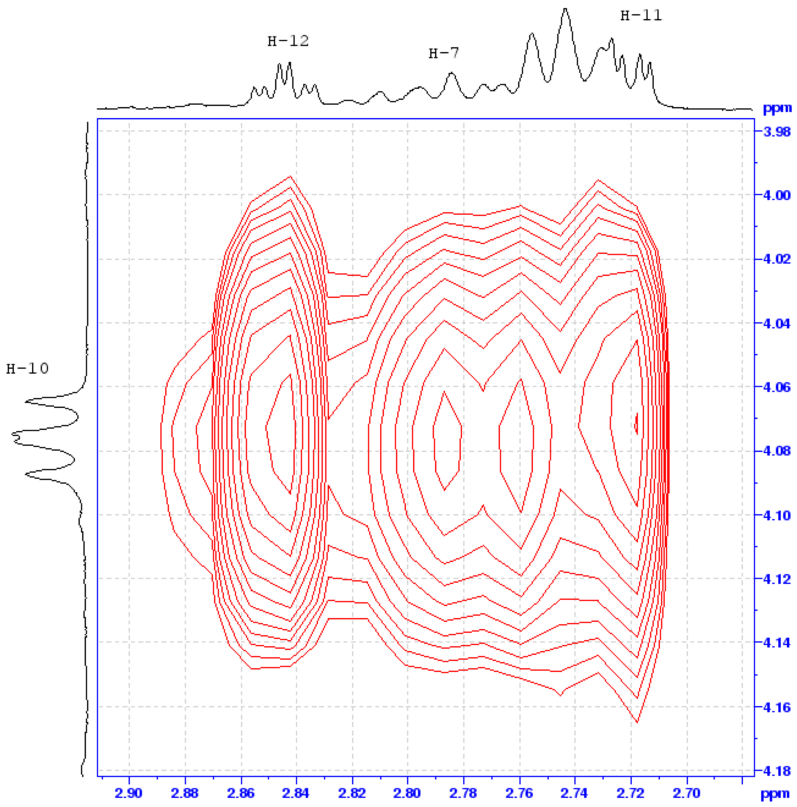

Supplementary Figure 20. ROESY NMR of hepoxilin B_4. ROE correlation of H-10, H-11, and H-12 of HXB_4 at ROESY.

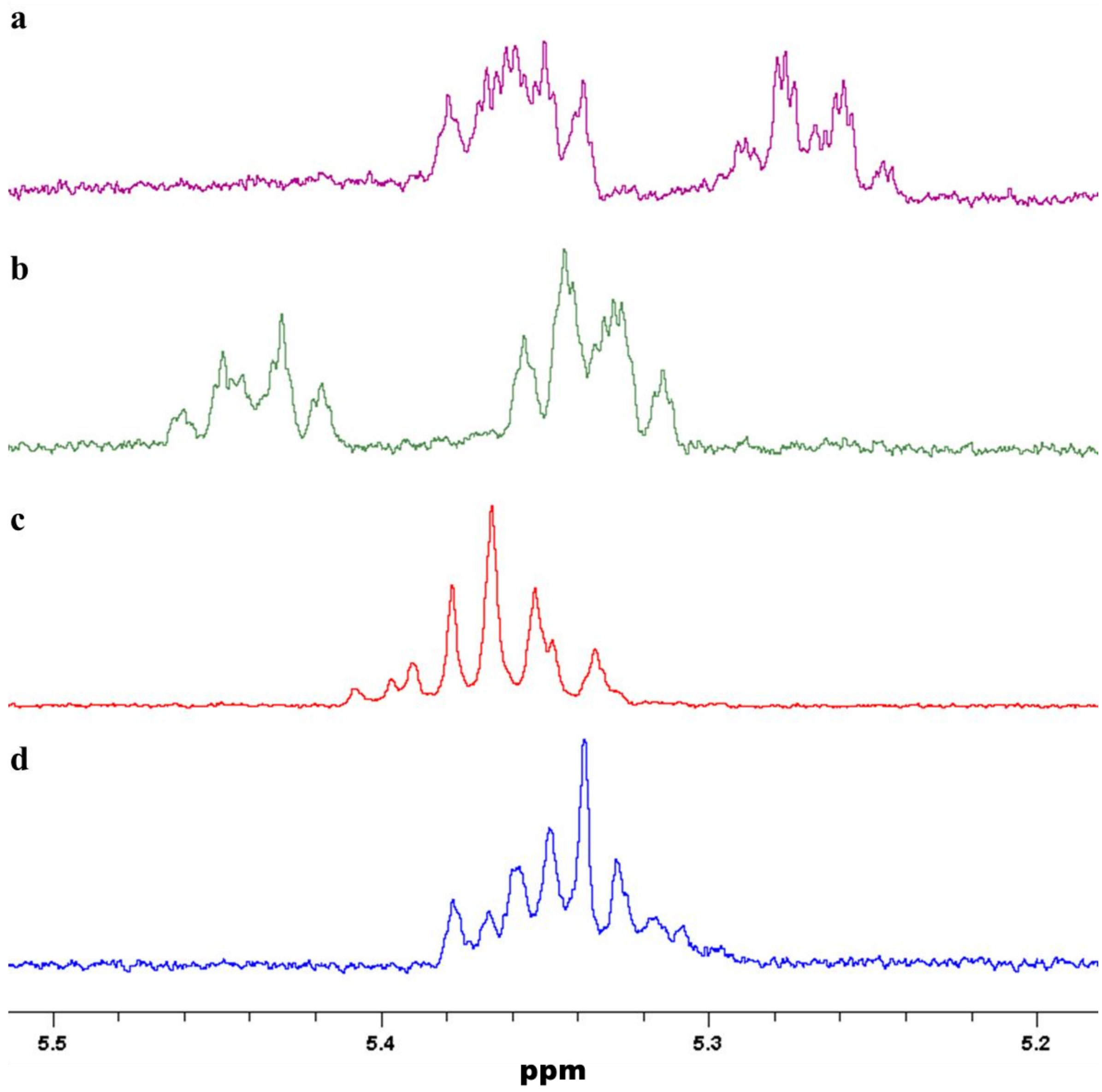

Supplementary Figure 21. H-5, H-6, H-8, H-9, H-14, H-15, H-17, and H-18 of hepoxilin B_4. The peaks were confirmed with the selective TOCSY (mixing time = 80 ms). **(a)** H-18,17 irradiation on H-20. **(b)** H-14,15 irradiation on H-12. **(c)** H-9,8 irradiation on H-10. **(d)** H-5,6 irradiation on H-3.

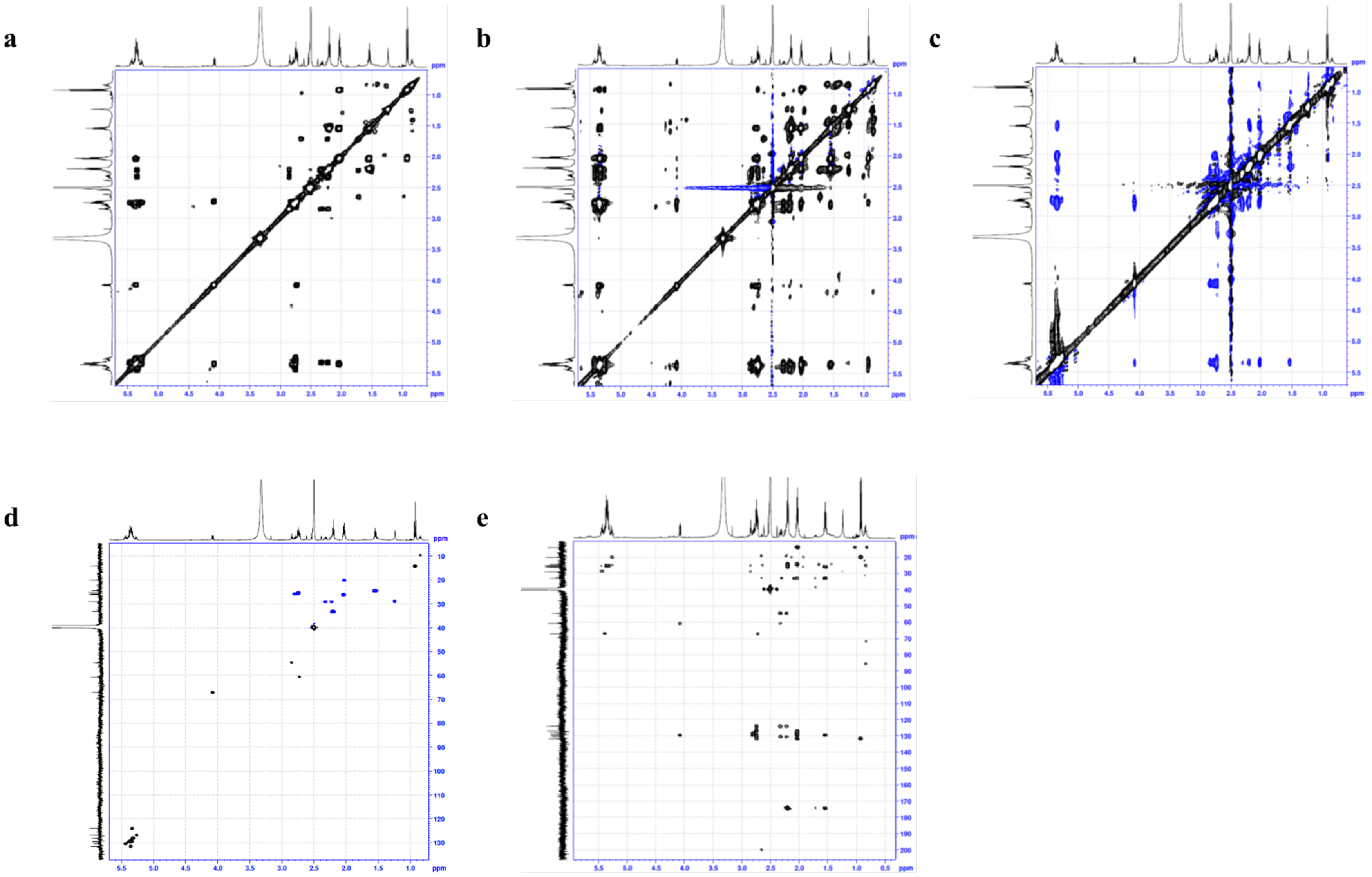

Supplementary Figure 22. 2D NMR of hepoxilin B$_4$. (a) COSY. **(b)** TOCSY. **(c)** ROESY. **(d)** HSQC. **(e)** HMBC.

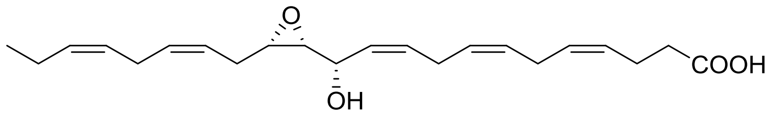

(*S*,4*Z*,7*Z*,10*Z*)-12-hydroxy-12-((2*R*,3*S*)-3-((2*Z*,5*Z*)-octa-2,5-dien-1-yl)oxiran-2-yl)dodeca-4,7,10-trienoic acid

Supplementary Figure 23. Structure of hepoxilin B_5.

a

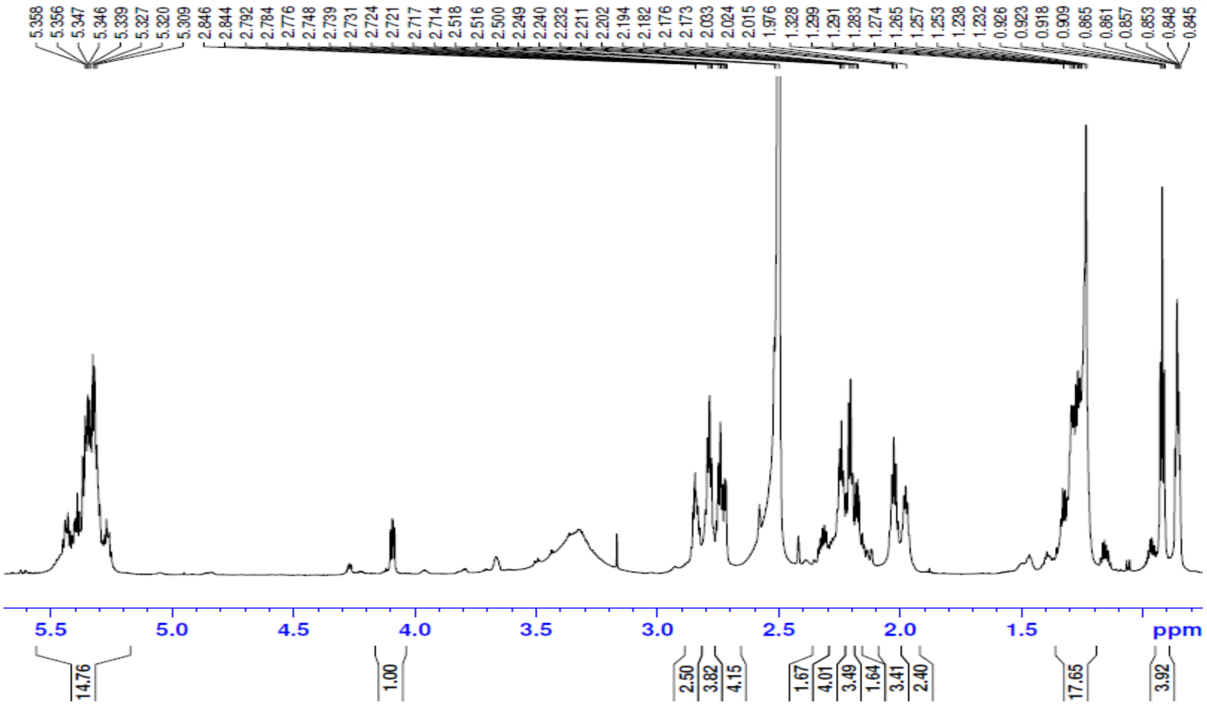

b

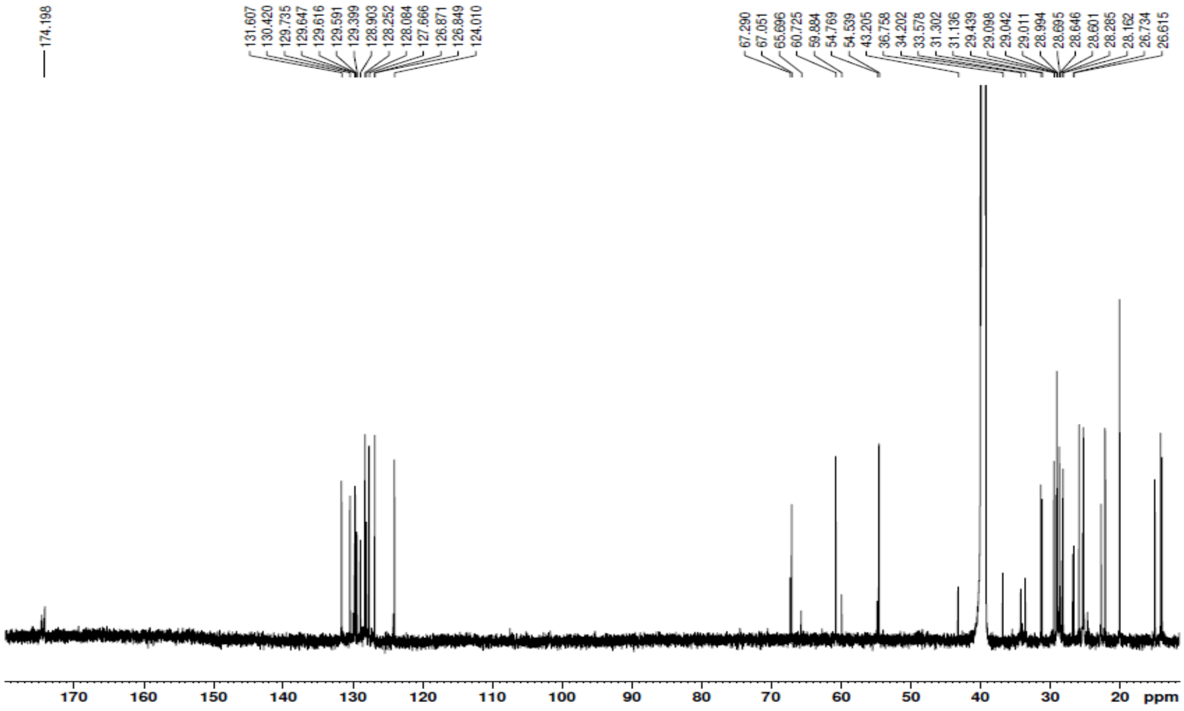

Supplementary Figure 24. 1D NMR data of hepoxilin B_5. (a) 1H NMR peak of HXB_5. **(b)** 13C NMR peak of HXB_5.

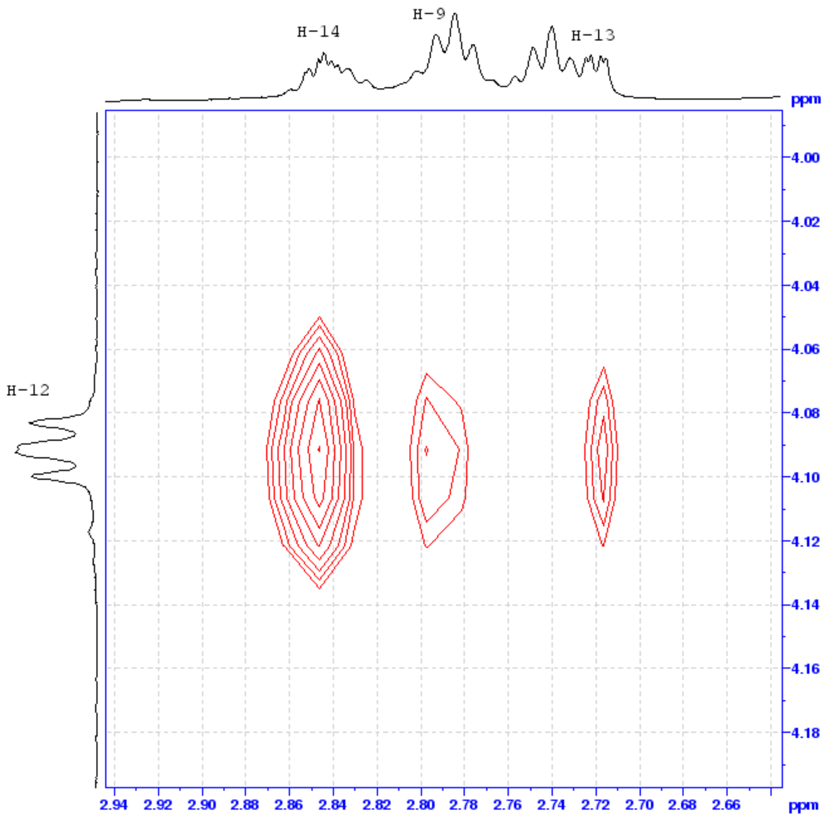

Supplementary Figure 25. ROESY NMR of hepoxilin B_5. ROE correlation of H-10, H-11, and H-12 of HxB_5 at ROESY.

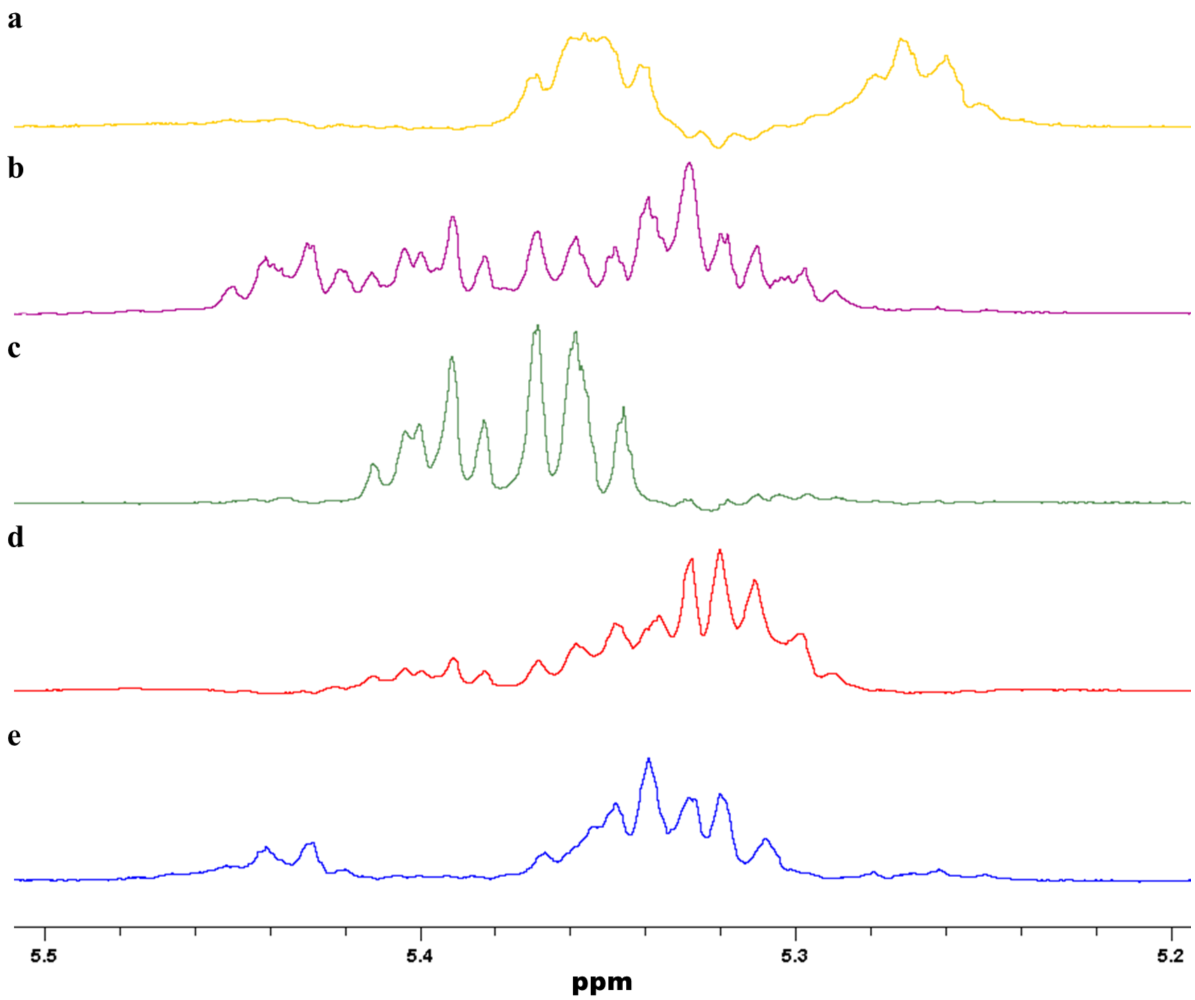

Supplementary Figure 26. H-4, H-5, H-7, H-8, H-10, H-11, H-17, H-19, and H-20 of hepoxilin B$_5$. The peaks were confirmed with the selective TOCSY (mixing time = 80 ms). **(a)** H-20,19 irradiation on H-22. **(b)** H-16,17 irradiation on H-14. **(c)** H-11,10 irradiation on H-12. **(d)** H-7,8 irradiation on H-6 and H-9. **(e)** H-4,5 irradiation on H-2.

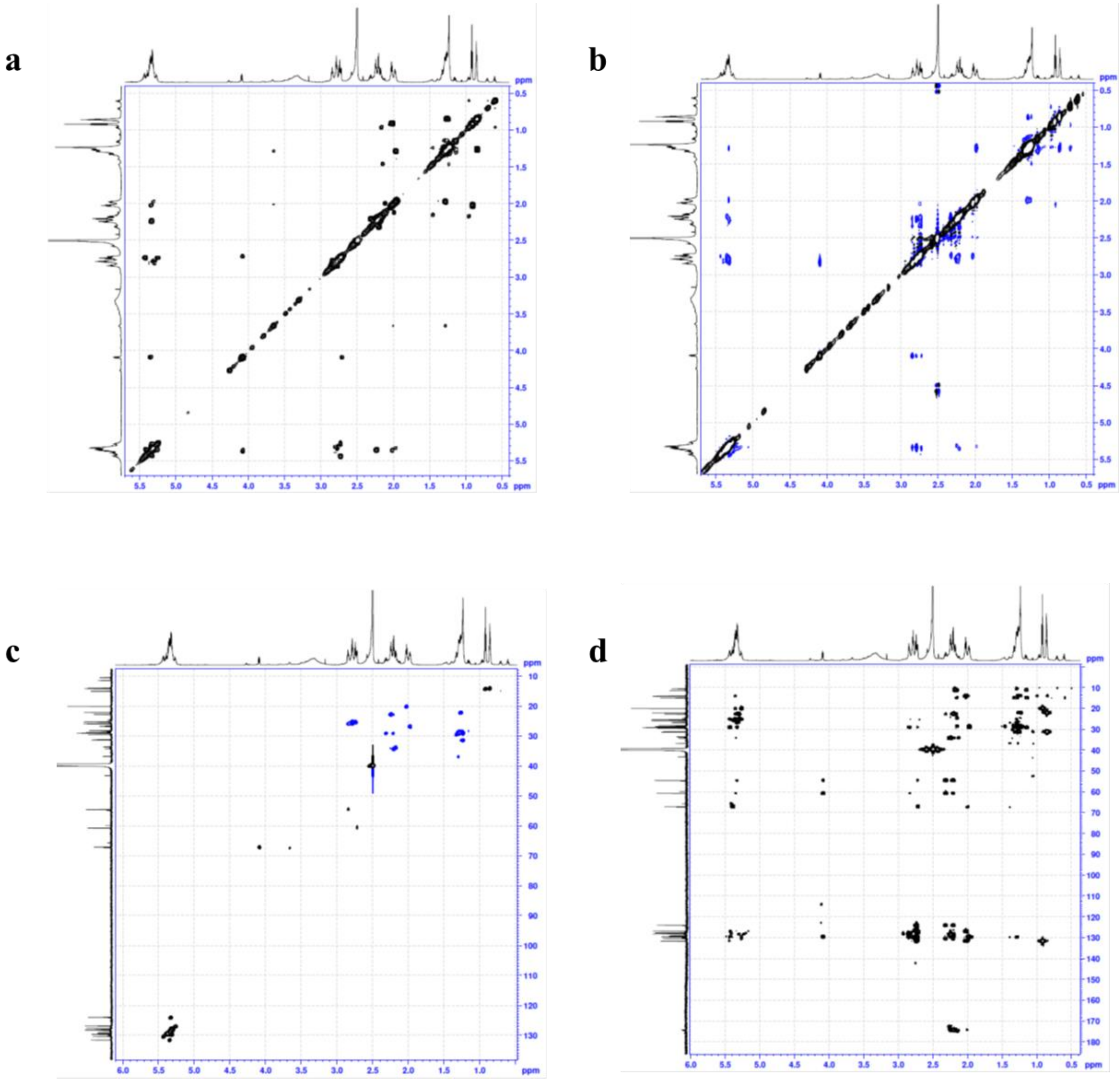

Supplementary Figure 27. 2D NMR of hepoxilin B_5. (a) COSY. **(b)** ROESY. **(c)** HSQC. **(d)** HMBC.

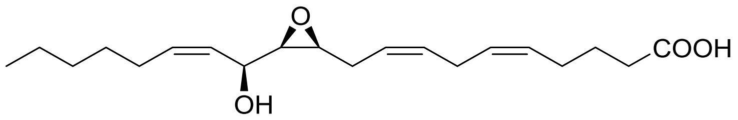

(5*Z*,8*Z*)-10-((2*S*,3*R*)-3-((*S*,*Z*)-1-hydroxyoct-2-en-1-yl)oxiran-2-yl)deca-5,8-dienoic acid

Supplementary Figure 28. Structure of hepoxilin D_3.

a

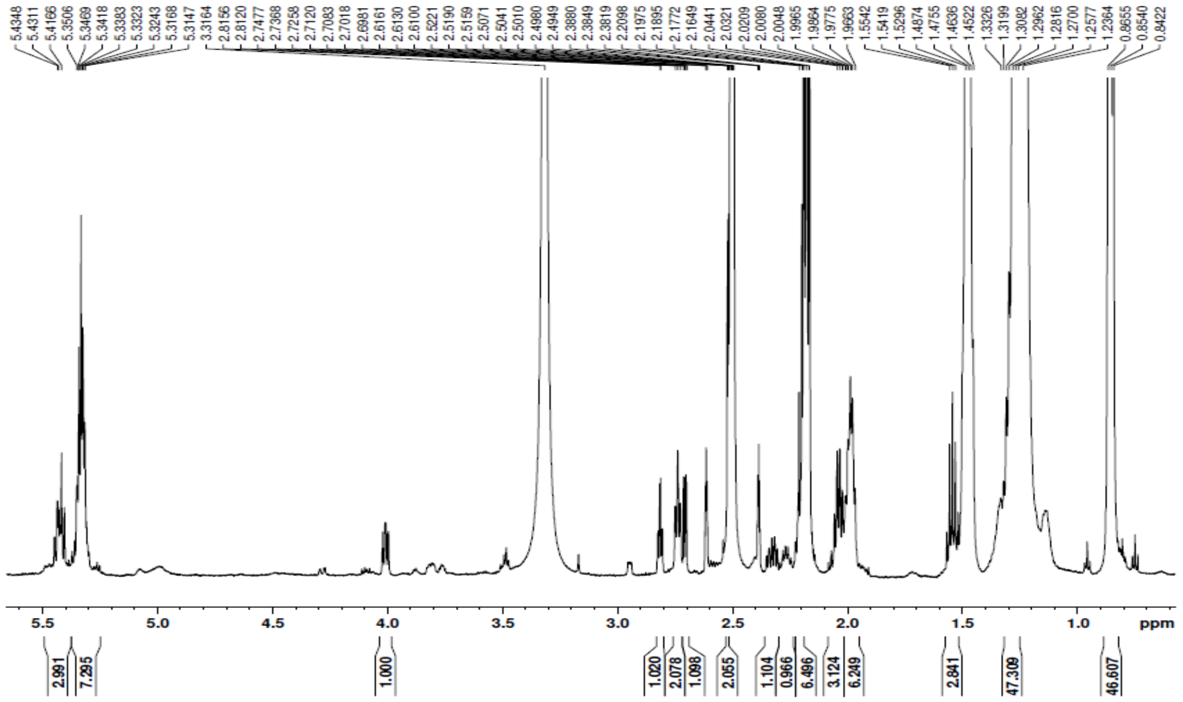

b

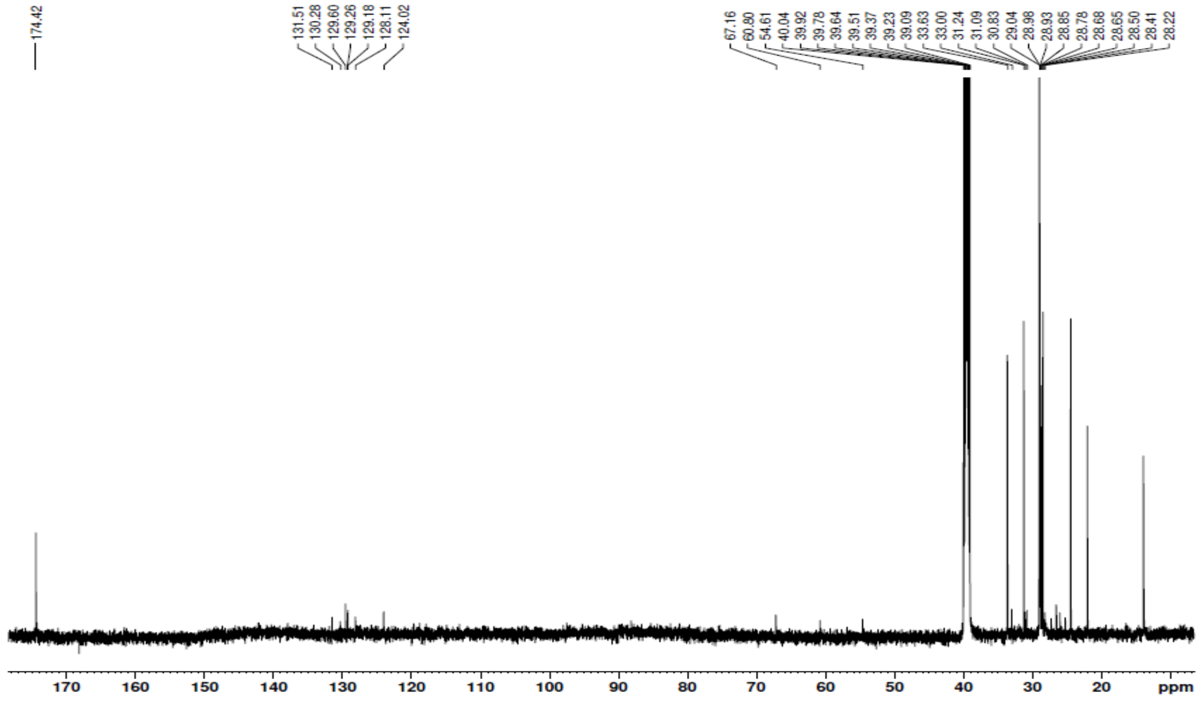

Supplementary Figure 29. 1D NMR data of hepoxilin D$_3$. (a) 1H NMR peak of HXD$_3$. **(b)** 13C NMR peak of HXD$_3$.

a

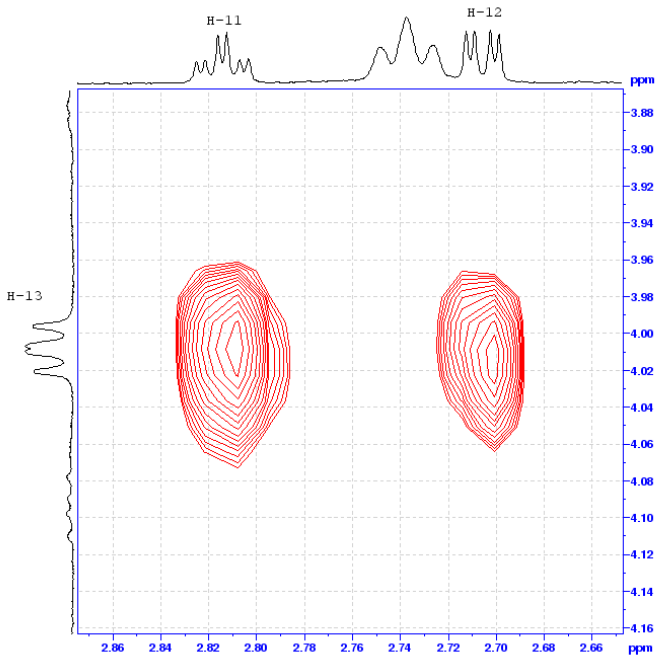

b

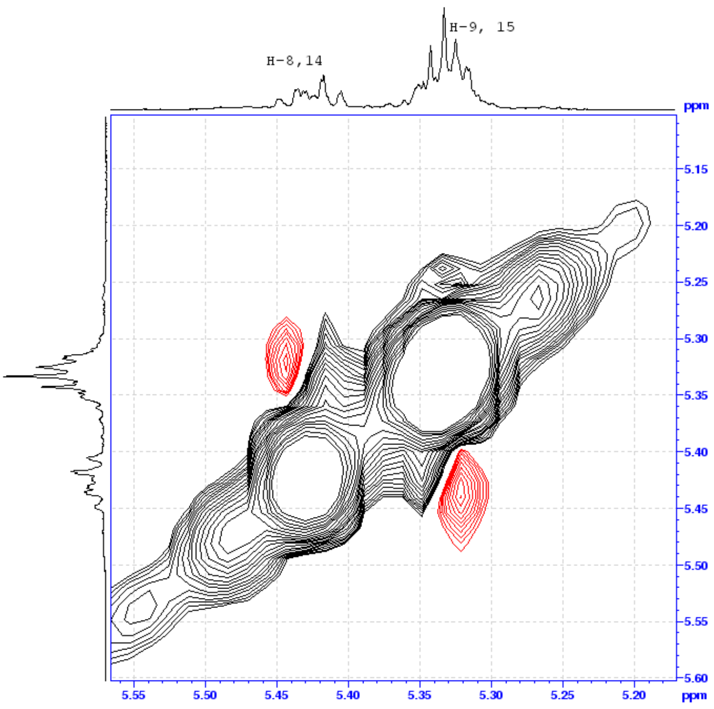

Supplementary Figure 30. ROESY NMR of hepoxilin D_3. (a) ROE correlation of H-11, H-12, and H-13 of HXD_3 at ROESY. **(b)** ROE correlation between H-14 and H-15 and between H-8 and H-9 of HXD_3 at ROESY.

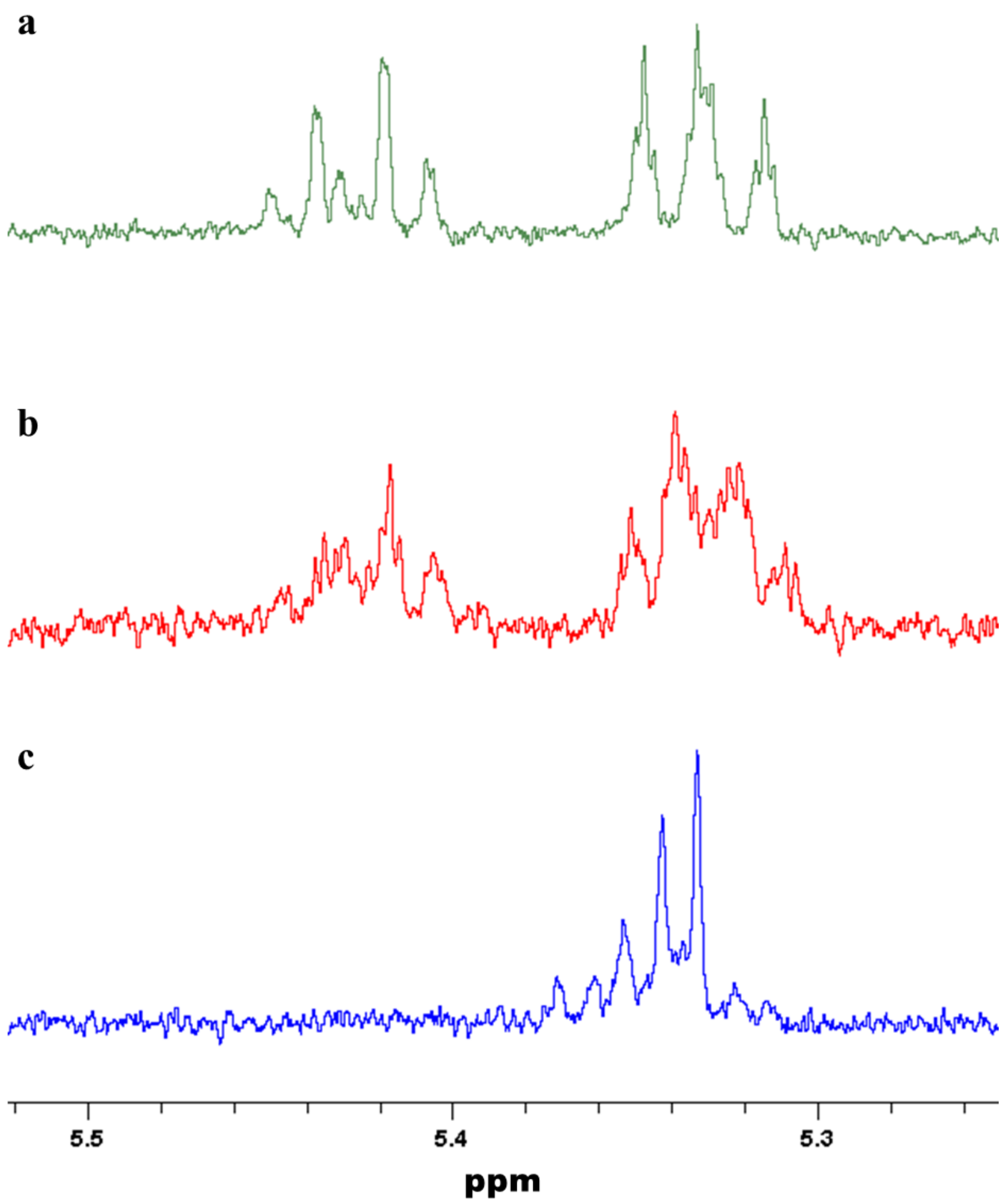

Supplementary Figure 31. H-5, H-6, H-8, H-9, H-14, and H-15 of hepoxilin D$_3$. The peaks were confirmed with the selective TOCSY (mixing time = 80 ms). **(a)** H-14,15 irradiation on H-13. **(b)** H-9,8 irradiation on H-11.**(c)** H-5,6 irradiation on H-3.

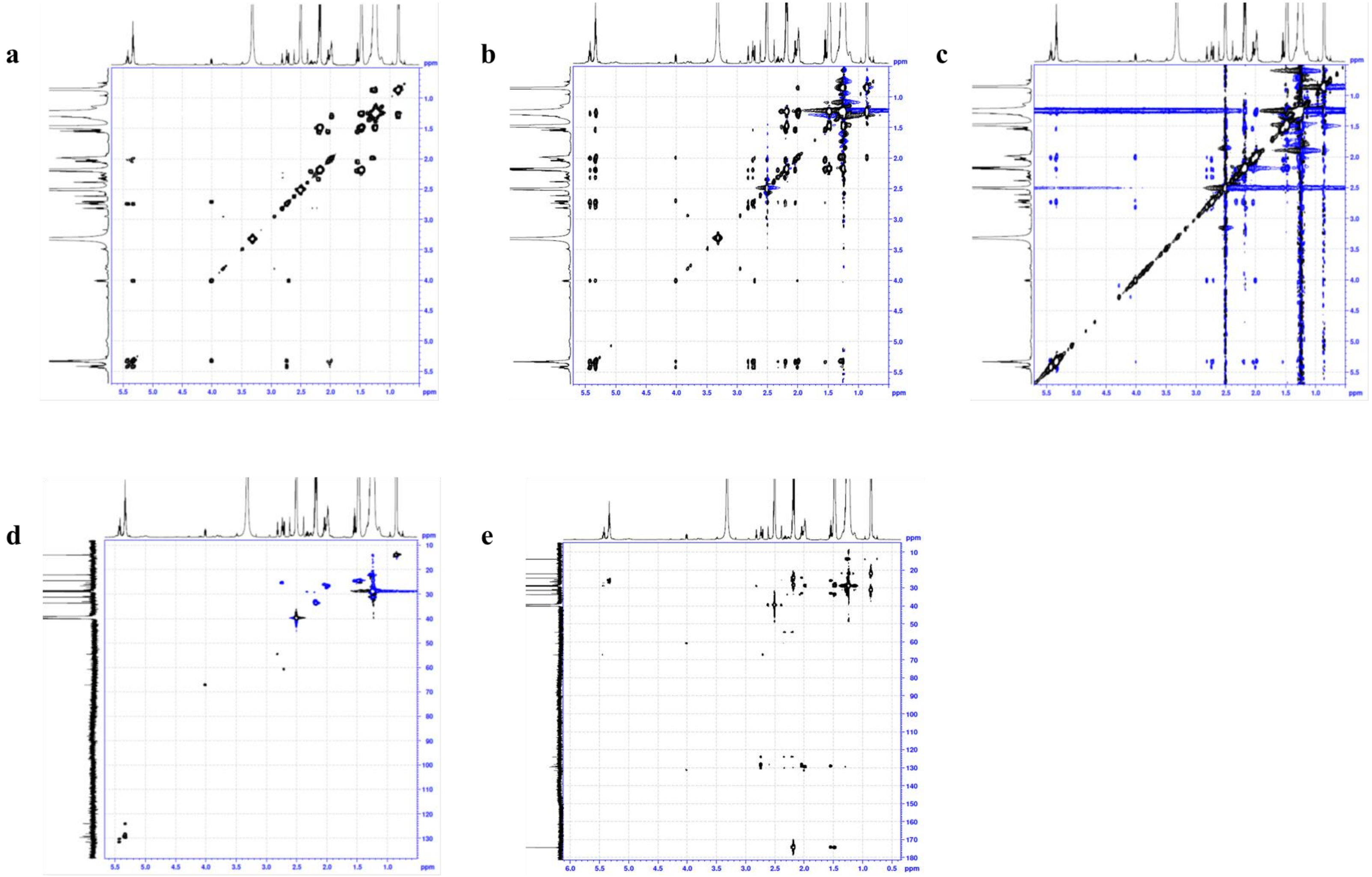

Supplementary Figure 32. 2D NMR of hepoxilin D_3. (a) COSY. **(b)** TOCSY. **(c)** ROESY. **(d)** HSQC. **(e)** HMBC.

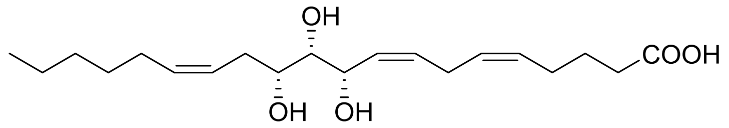

(5*Z*,8*Z*,10*S*,11*S*,12*R*,14*Z*)-10,11,12-trihydroxyicosa-5,8,14-trienoic acid

Supplementary Figure 33. Structure of trioxilin B_3.

a

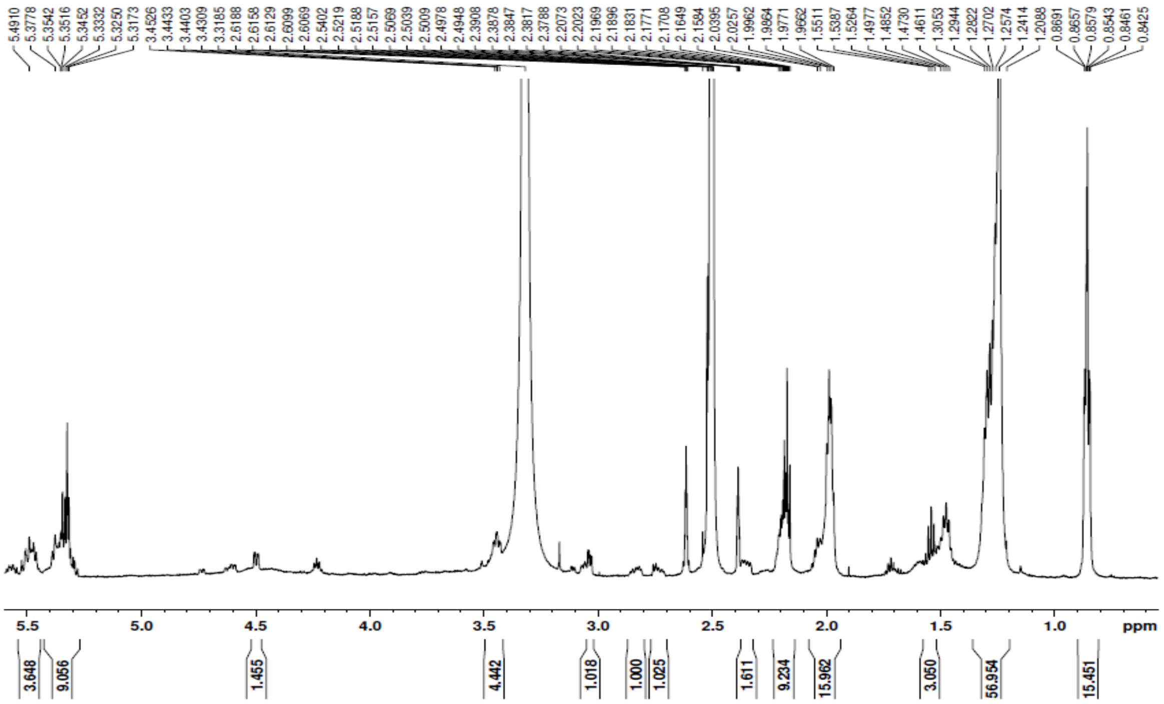

b

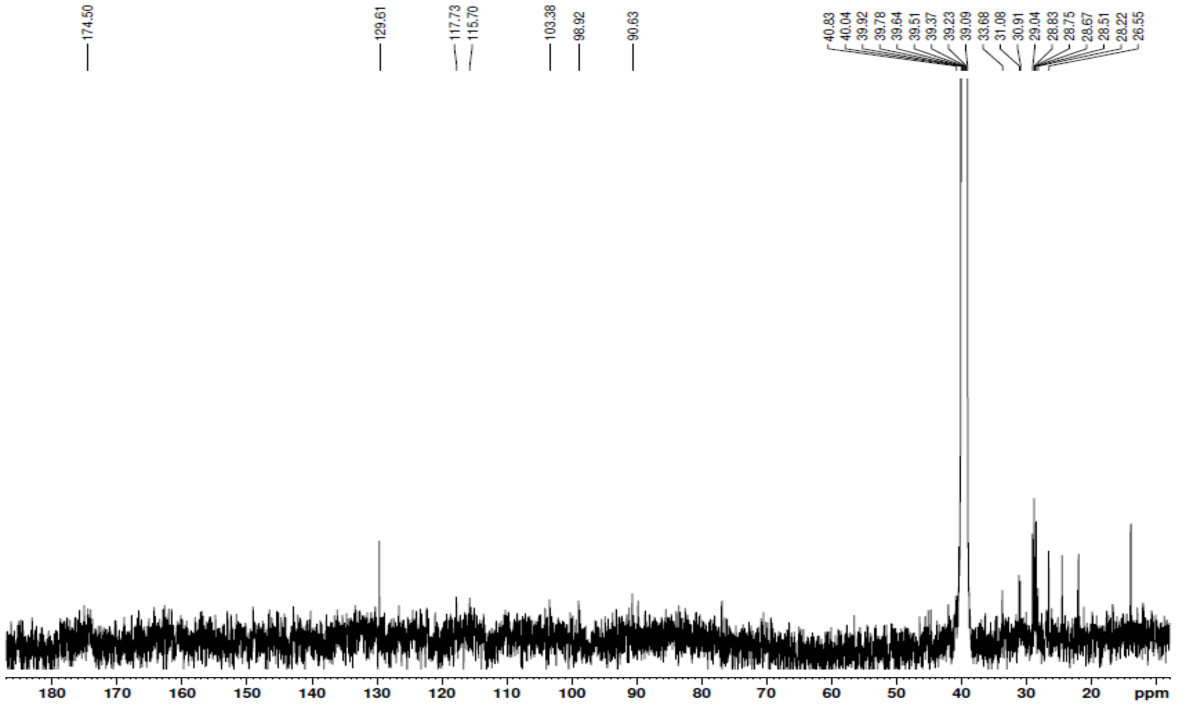

Supplementary Figure 34. 1D NMR data of trioxilin B_3. (a) 1H NMR peak of $TrXB_3$. **(b)** 13C NMR peak of $TrXB_3$.

a

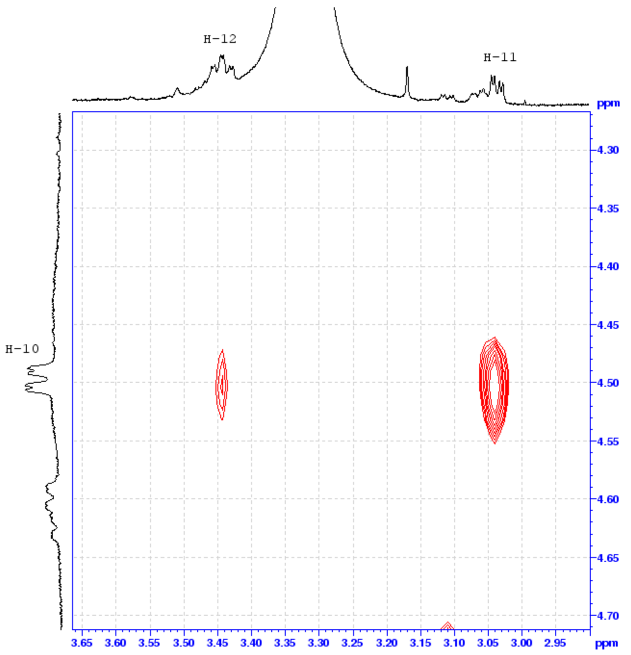

b

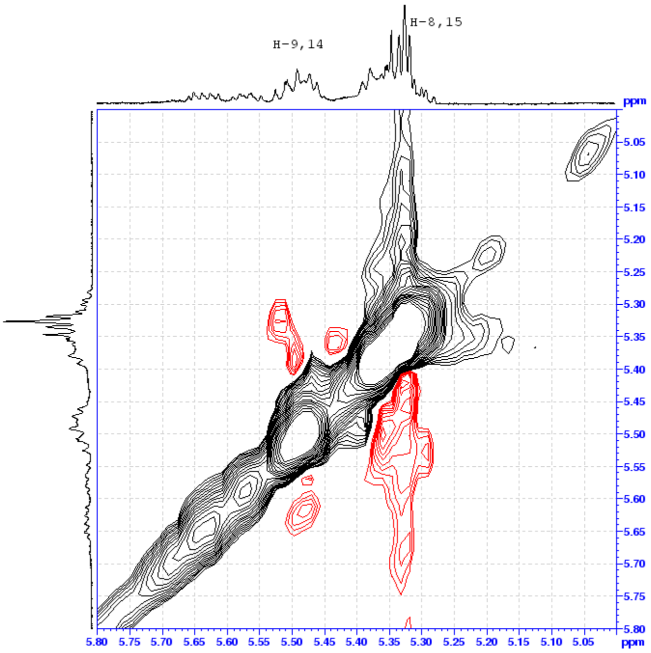

Supplementary Figure 35. ROESY NMR of trioxilin B$_3$. (a) ROE correlation of H-10, H-11, and H-12 of HxD3 at ROESY. **(b)** ROE correlation of between H-14 and H-15 and between H-8 and H-9 of TrXB3 at ROESY.

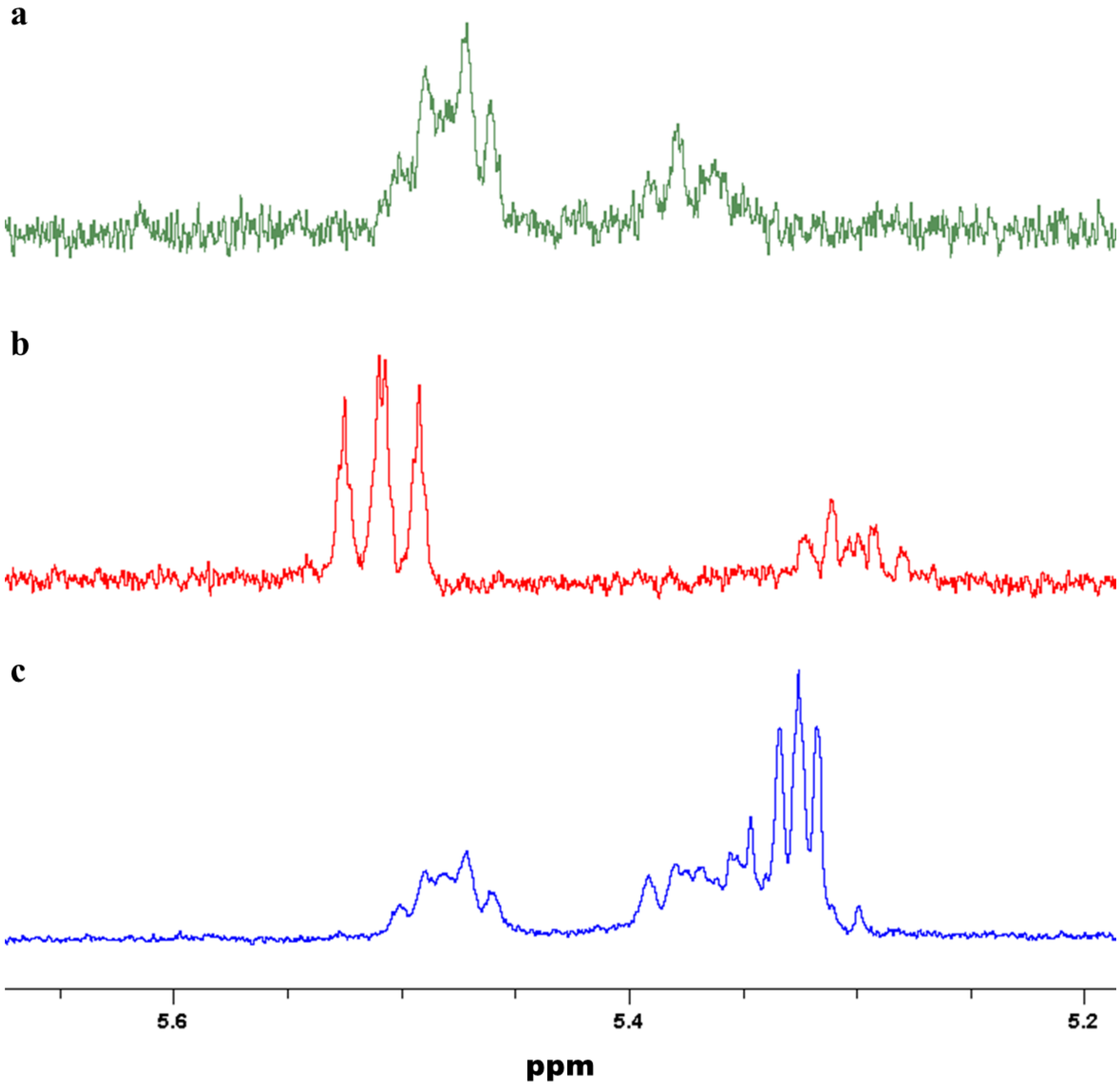

Supplementary Figure 36. H-5, H-6, H-8, H-9, H-14, and H-15 of trioxilin B$_3$. The peaks were confirmed with the selective TOCSY (mixing time = 40 ms). **(a)** H-14,15 irradiation on H-13. **(b)** H-9,8 irradiation on H-10. **(c)** H-5,6 irradiation on H-2.

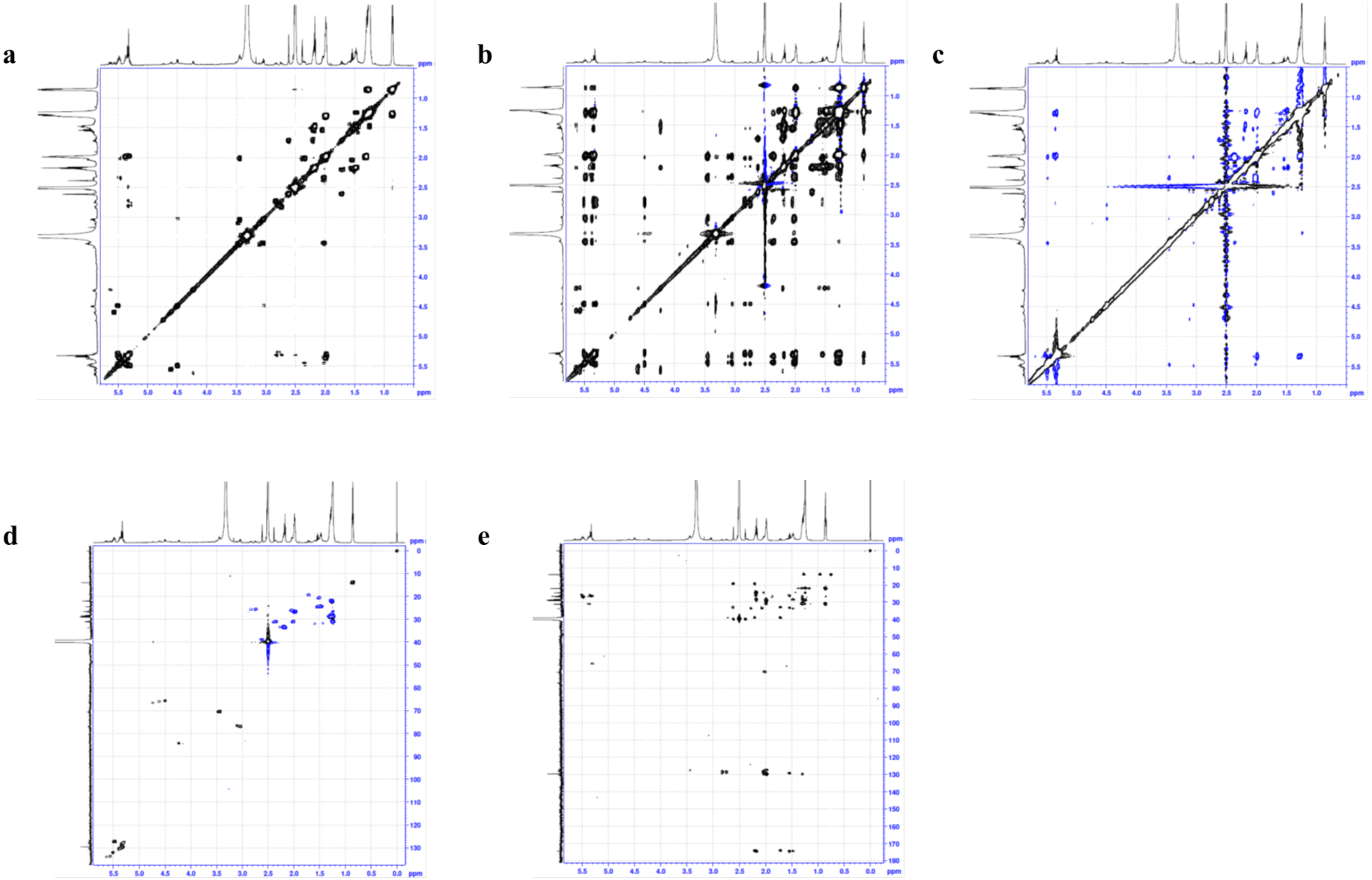

Supplementary Figure 37. 2D NMR of trioxilin B_3. (a) COSY. **(b)** TOCSY. **(c)** ROESY. **(d)** HSQC. **(e)** HMBC.

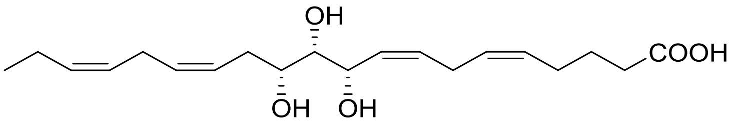

(5*Z*,8*Z*,10*S*,11*S*,12*R*,14*Z*,17*Z*)-10,11,12-trihydroxyicosa-5,8,14,17-tetraenoic acid

Supplementary Figure 38. Structure of trioxilin B_4.

a

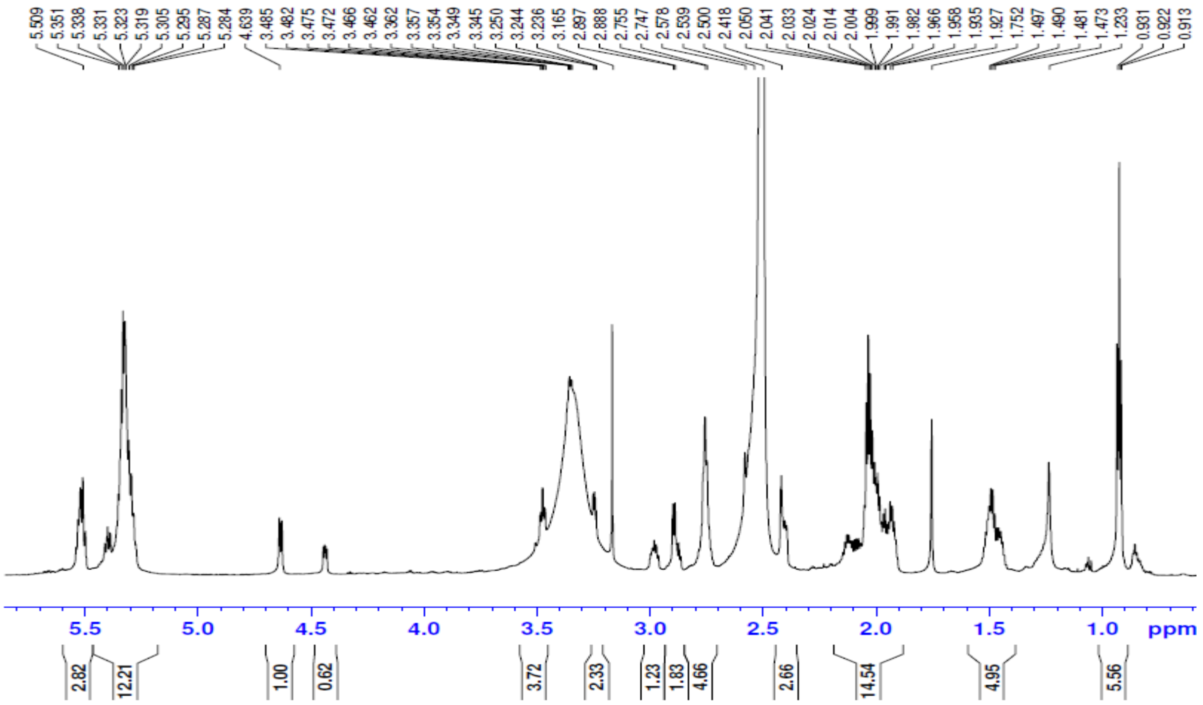

b

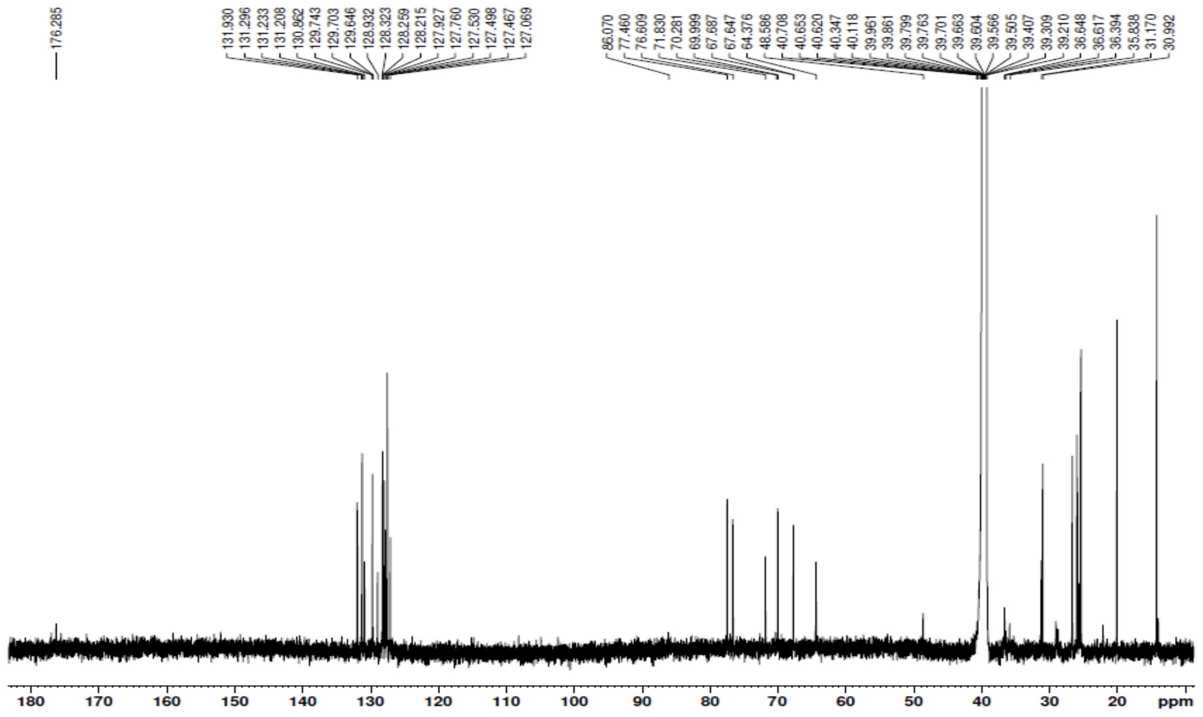

Supplementary Figure 39. 1D NMR data of trioxilin B_4. (a) 1H NMR peak of $TrXB_4$. **(b)** 13C NMR peak of $TrXB_4$.

a

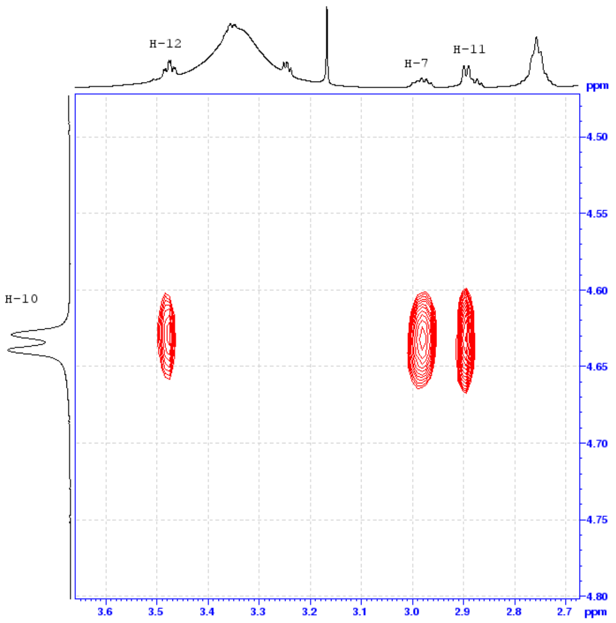

b

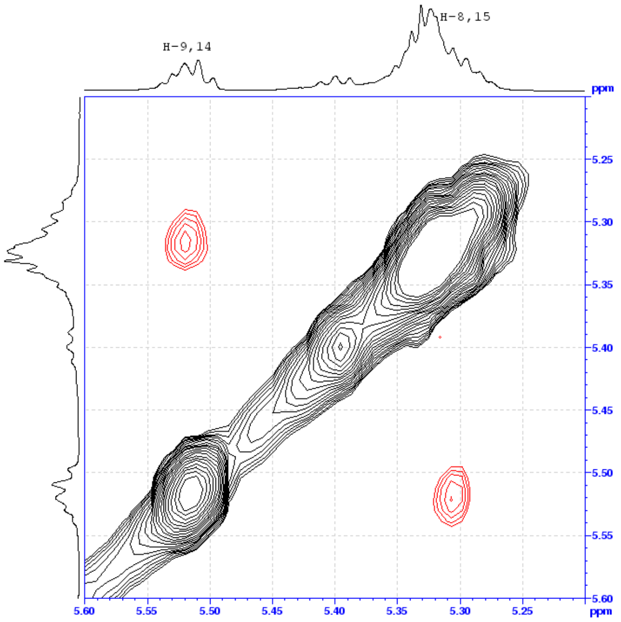

Supplementary Figure 40. ROESY NMR of trioxilin B$_4$. (a) ROE correlation of H-10, H-11, and H-12 of TrXB4 at ROESY. **(b)** ROE correlation of between H-14 and H-15 and between H-8 and H-9 of TrXB4 at ROESY.

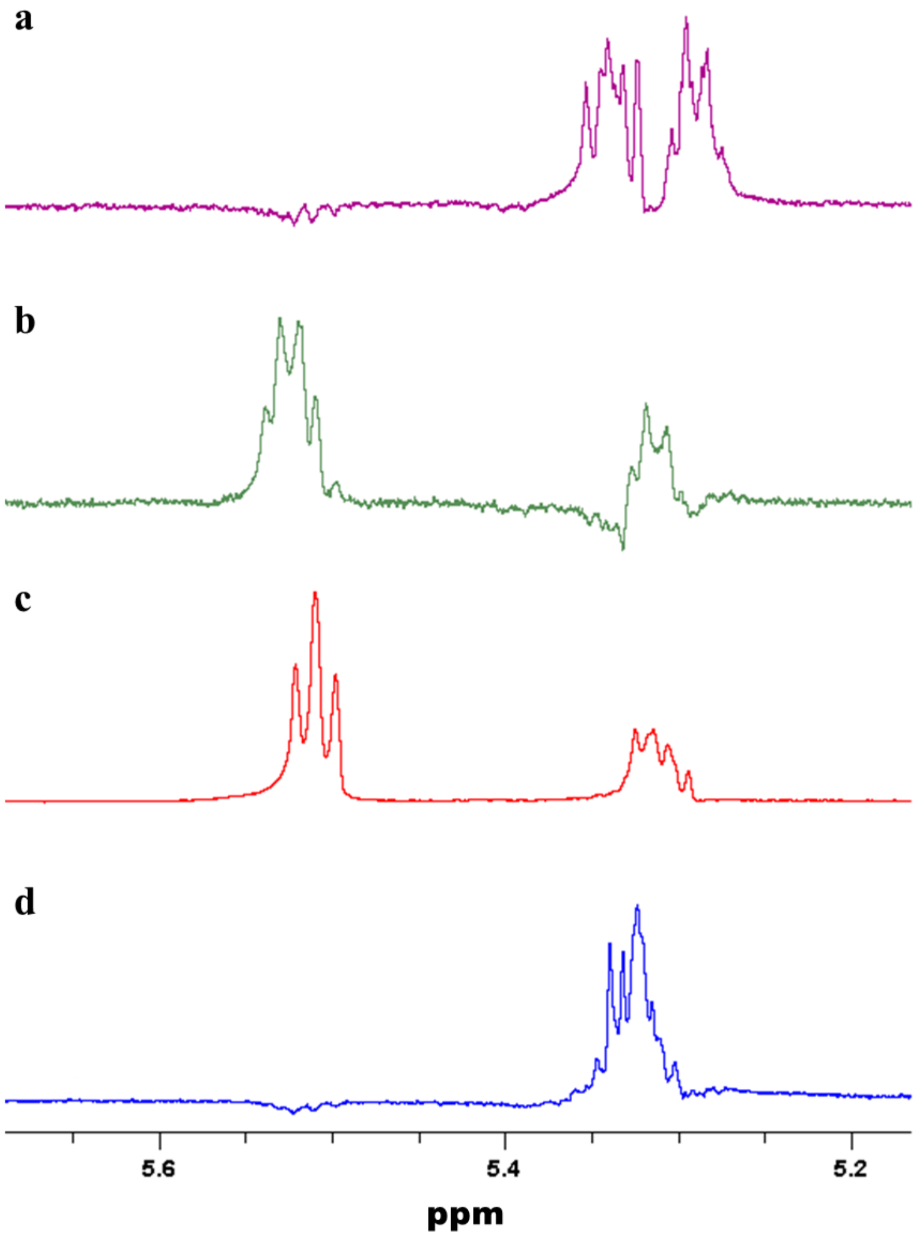

Supplementary Figure 41. H-5, H-6, H-8, H-9, H-14, H-15, H-17, and H-18 of trioxilin B_4. The peaks were confirmed with the selective TOCSY (mixing time = 80 ms). **(a)** H-5,6 irradiation on H-3. **(b)** H-9,8 irradiation on H-10. **(c)** H-14,15 irradiation on H-12. **(d)** H-18,17 irradiation on H-20.

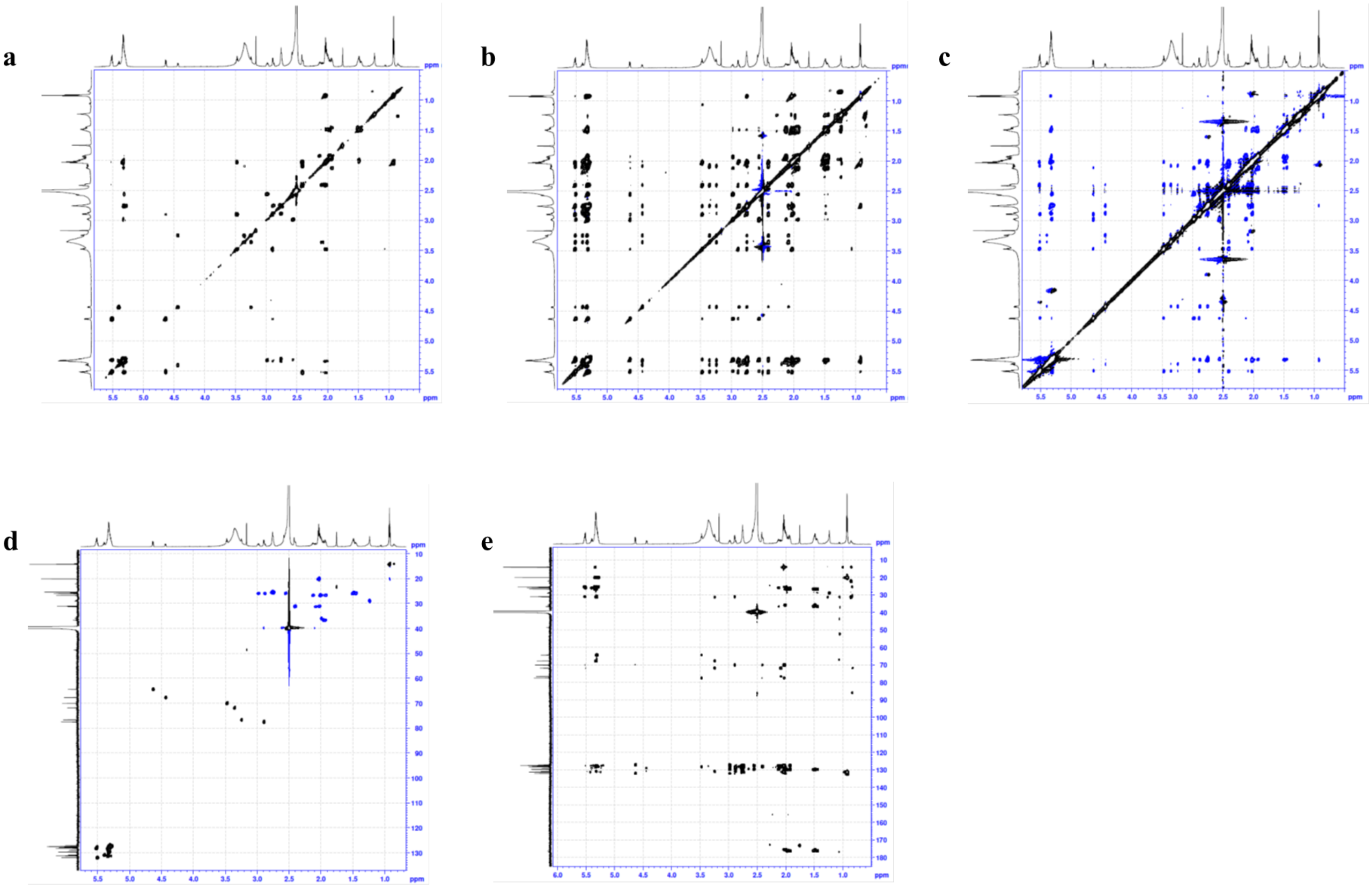

Supplementary Figure 42. 2D NMR of trioxilin B$_4$. (a) COSY. **(b)** TOCSY. **(c)** ROESY. **(d)** HSQC. **(e)** HMBC.

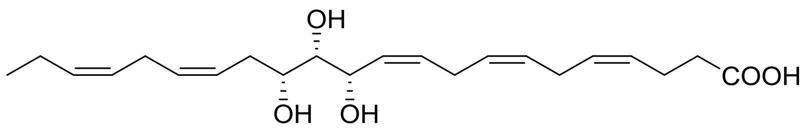

(4*Z*,7*Z*,10*Z*,12*S*,13*S*,14*R*,16*Z*,19*Z*)-12,13,14-trihydroxydocosa-4,7,10,16,19-pentaenoic acid

Supplementary Figure 43. Structure of trioxilin B_5.

a

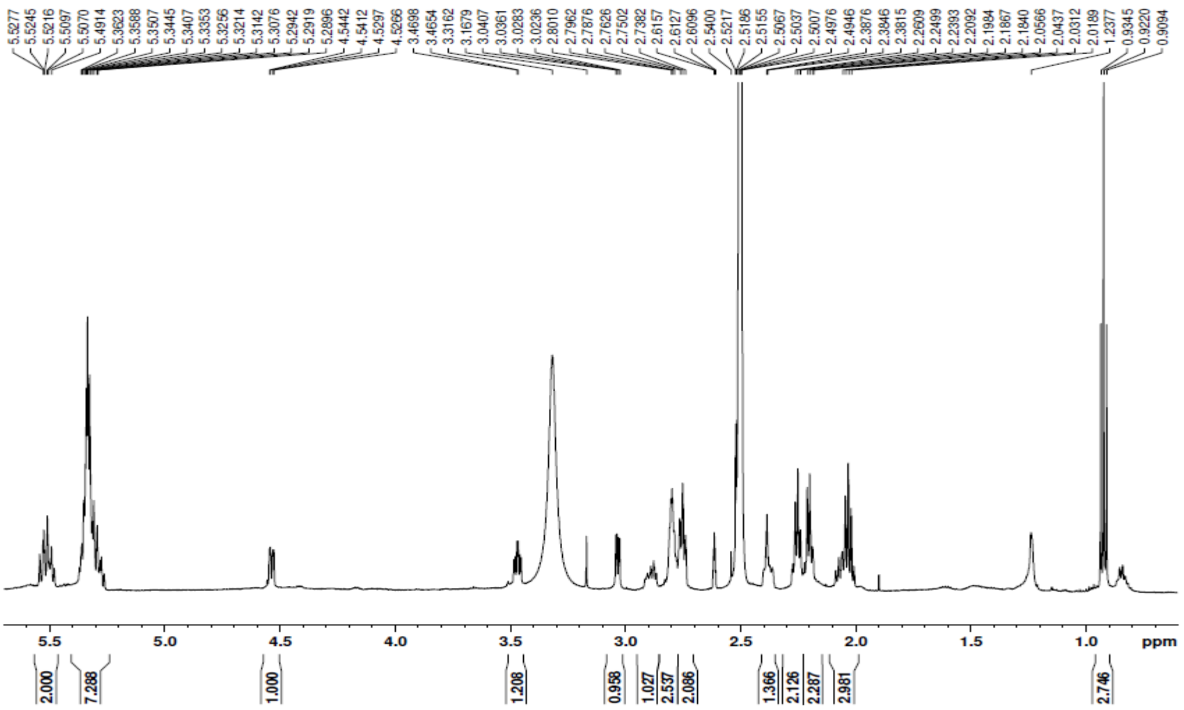

b

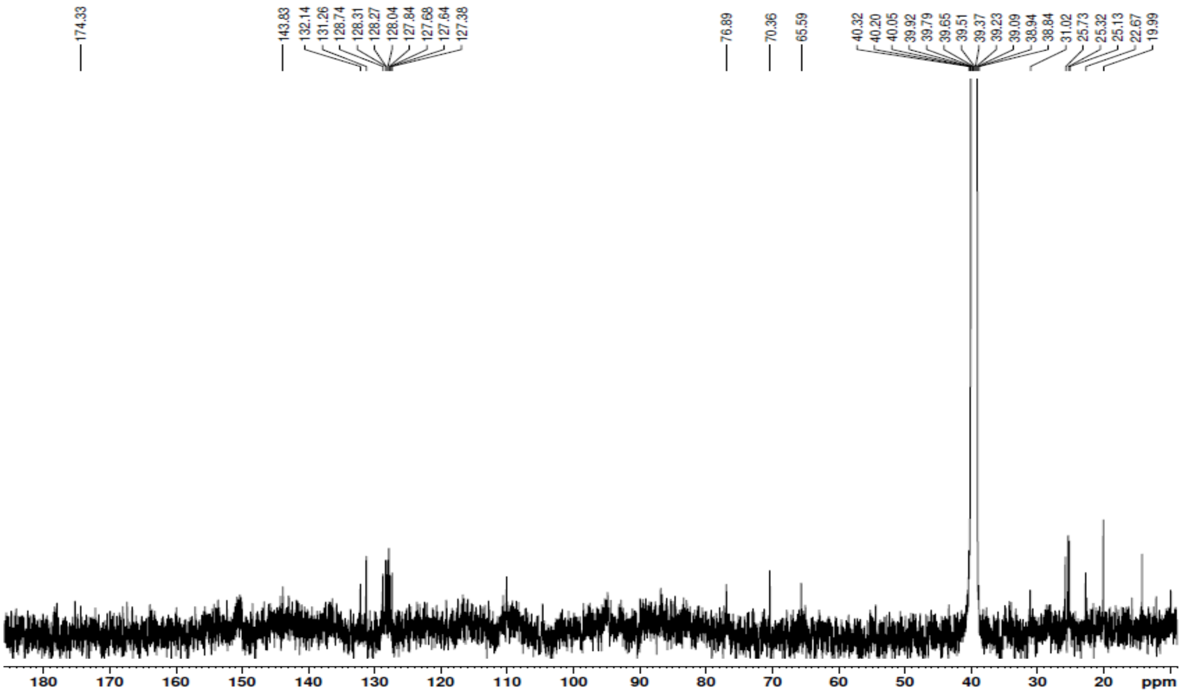

Supplementary Figure 44. 1D NMR data of trioxilin B$_5$. (a) 1H NMR peak of TrXB$_5$. **(b)** 13C NMR peak of TrXB$_5$.

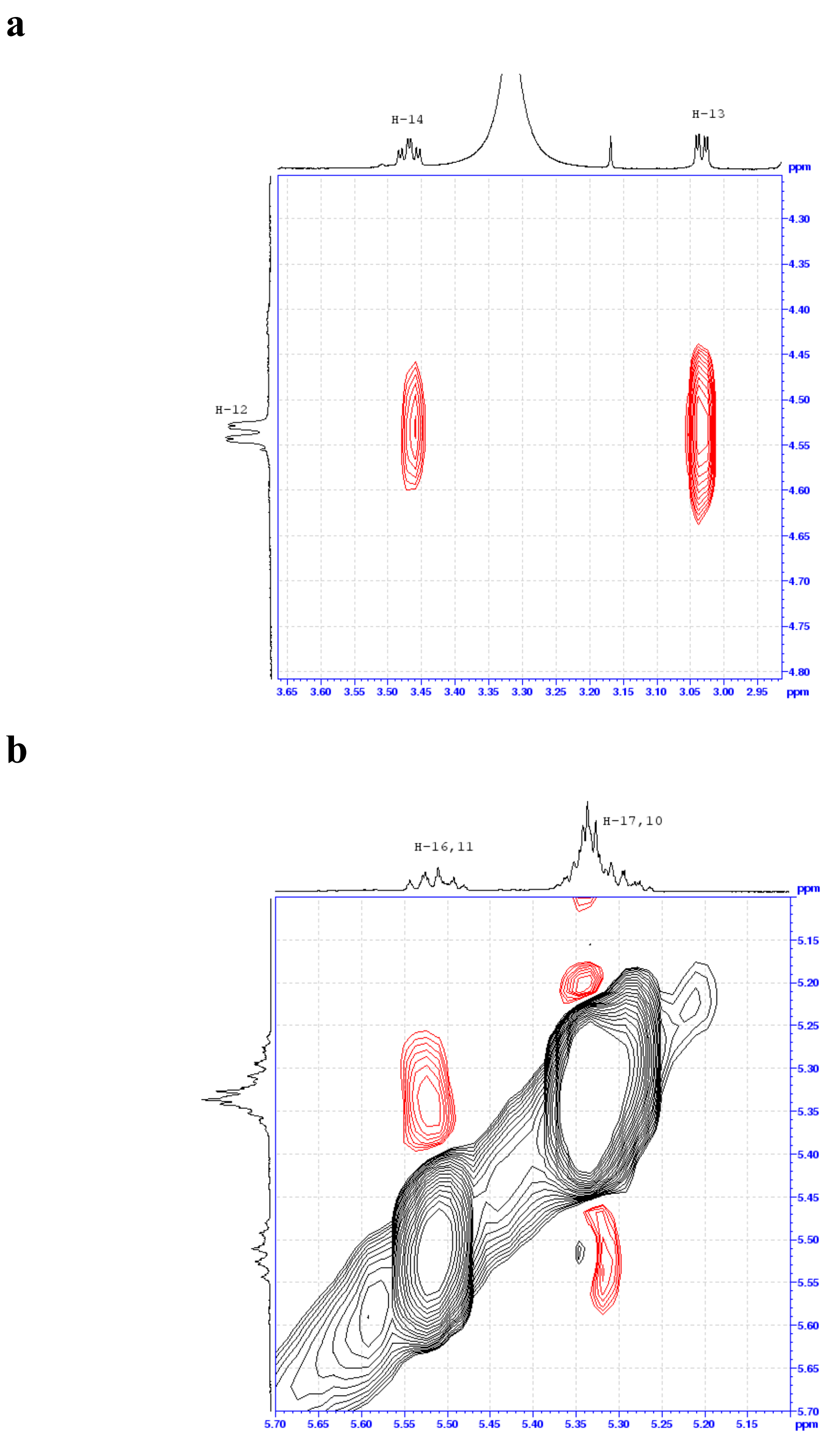

Supplementary Figure 45. ROESY NMR of trioxilin B$_5$. (a) ROE correlation of H-12, H-13, and H-14 of TrXB5 at ROESY. **(b)** ROE correlation of between H-16 and H-17 and between H-10 and H-11 of TrXB5 at ROESY.

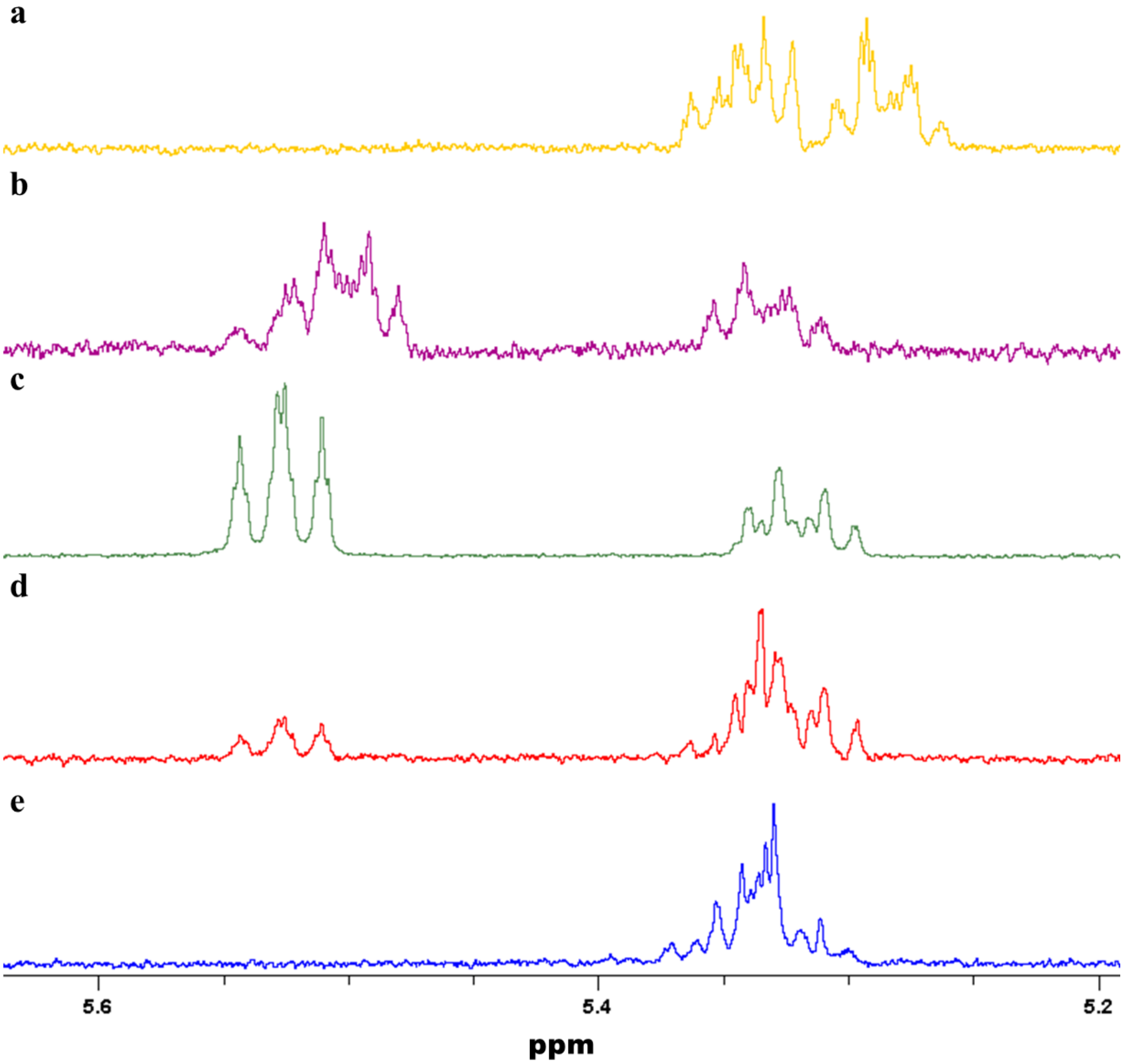

Supplementary Figure 46. H-4, H-5, H-7, H-8, H-10, H-11, H-17, H-19, and H-20 of trioxilin B_5. The peaks were confirmed with the selective TOCSY (mixing time = 80 ms). **(a)** H-20,19 irradiation on H-22. **(b)** H-16,17 irradiation on H-14. **(c)** H-11,10 irradiation on H-12. **(d)** H-8,7 irradiation on H-9. **(e)** H-4,5 irradiation on H-2.

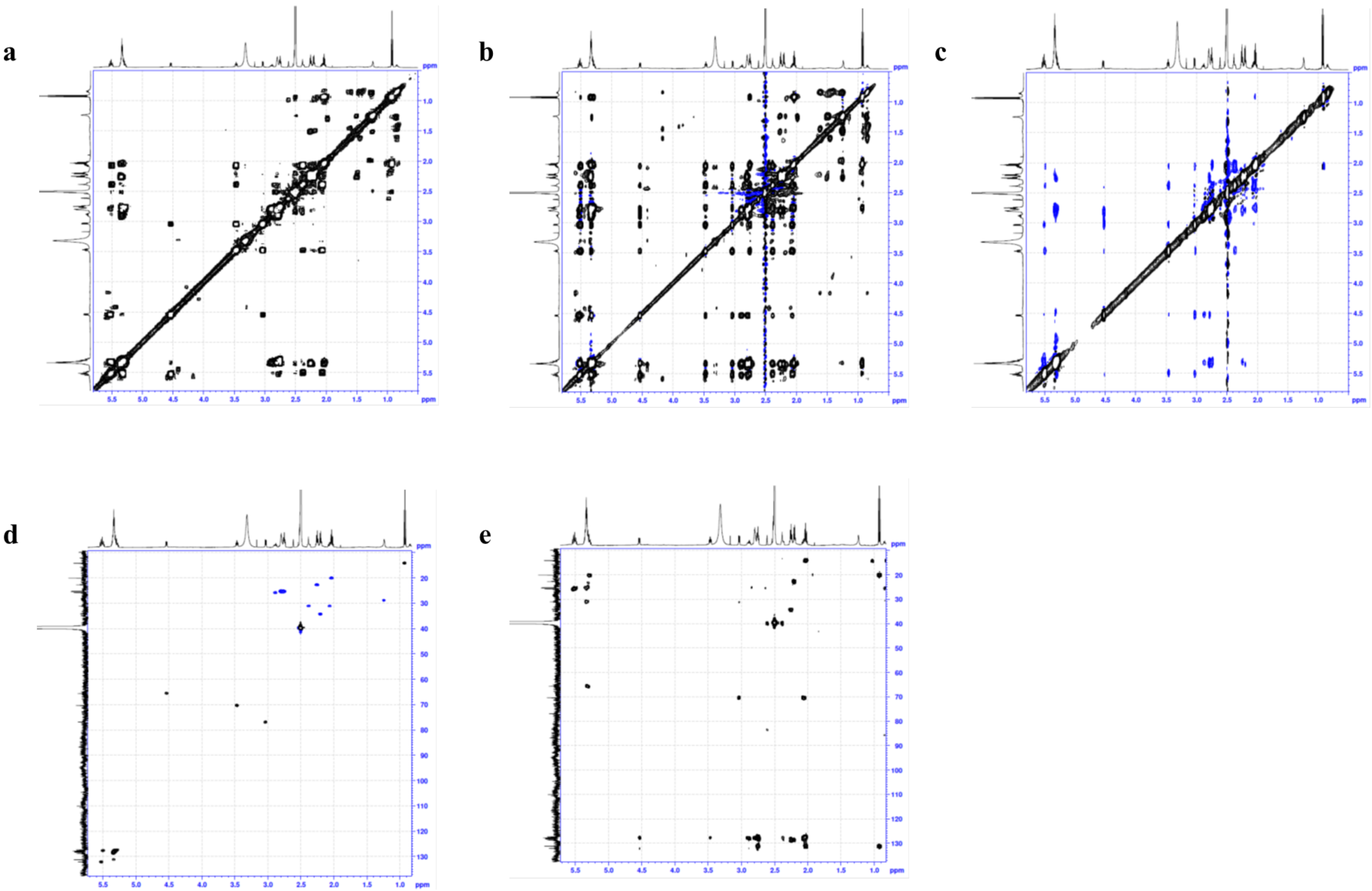

Supplementary Figure 47. 2D NMR of trioxilin B$_5$. (a) COSY. **(b)** TOCSY. **(c)** ROESY. **(d)** HSQC. **(e)** HMBC.

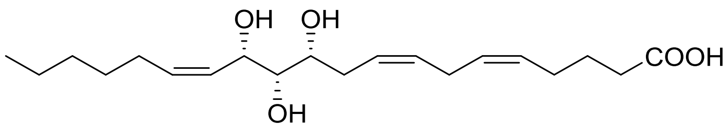

(5*Z*,8*Z*,11*R*,12*S*,13*S*,14*Z*)-11,12,13-trihydroxyicosa-5,8,14-trienoic acid

Supplementary Figure 48. Structure of trioxilin D_3.

a

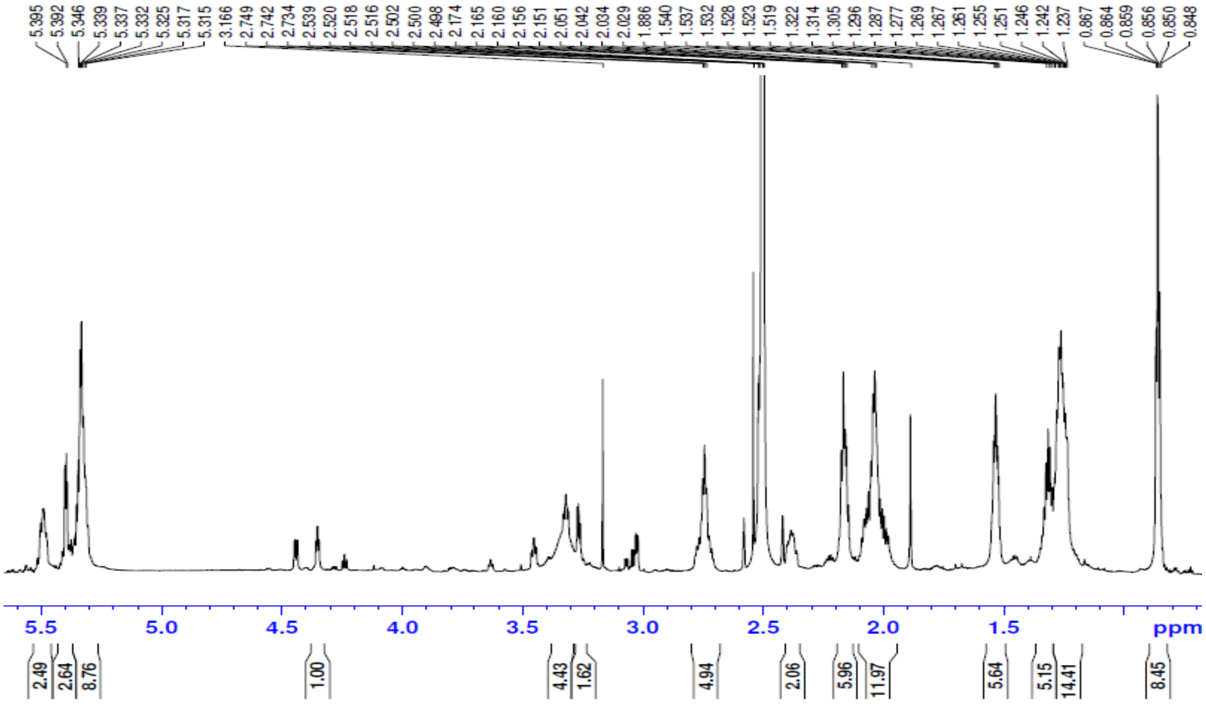

b

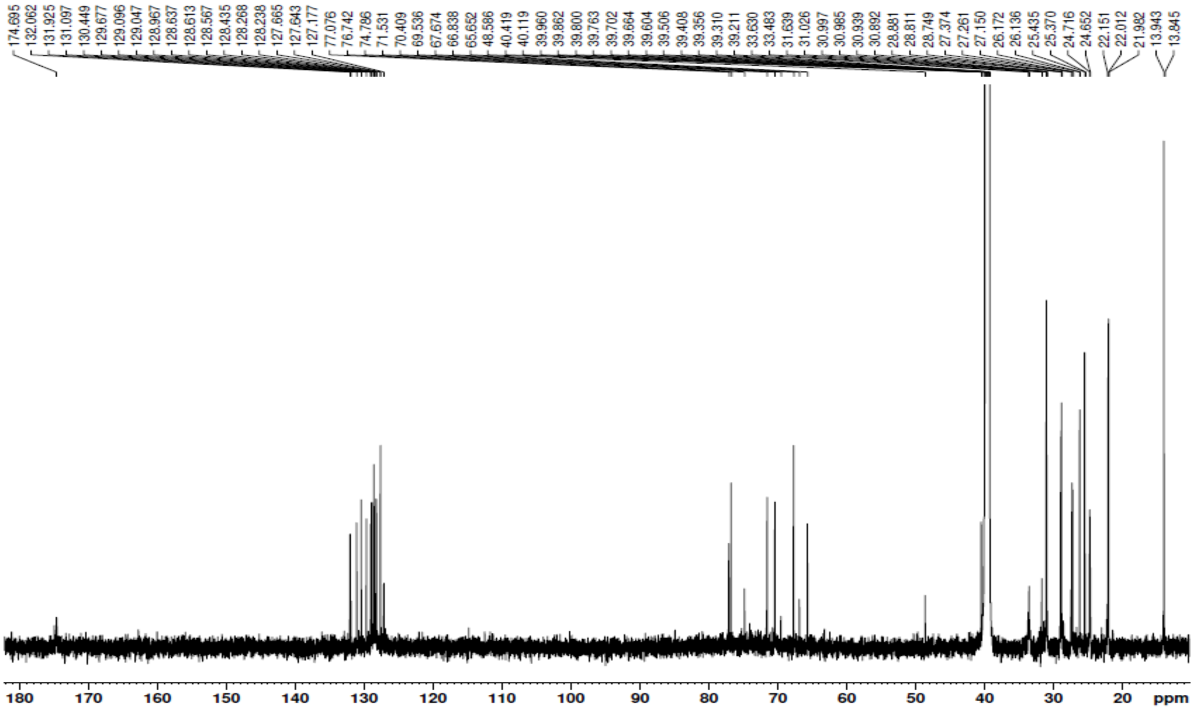

Supplementary Figure 49. 1D NMR data of trioxilin D_3. (a) 1H NMR peak of $TrXD_3$. **(b)** 13C NMR peak of $TrXD_3$.

a

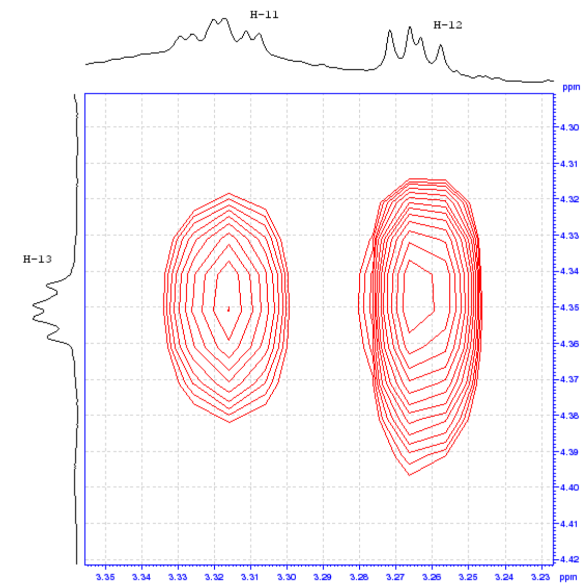

b

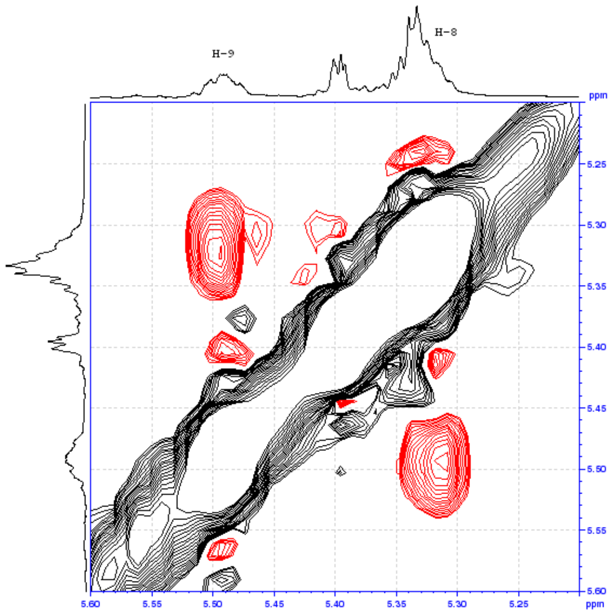

Supplementary Figure 50. ROESY NMR of trioxilin D_3. (a) ROE correlation of H-11, H-12, and H-13 of TrXD3 at ROESY. **(b)** ROE correlation of between H-8 and H-9 of TrXD3 at ROESY.

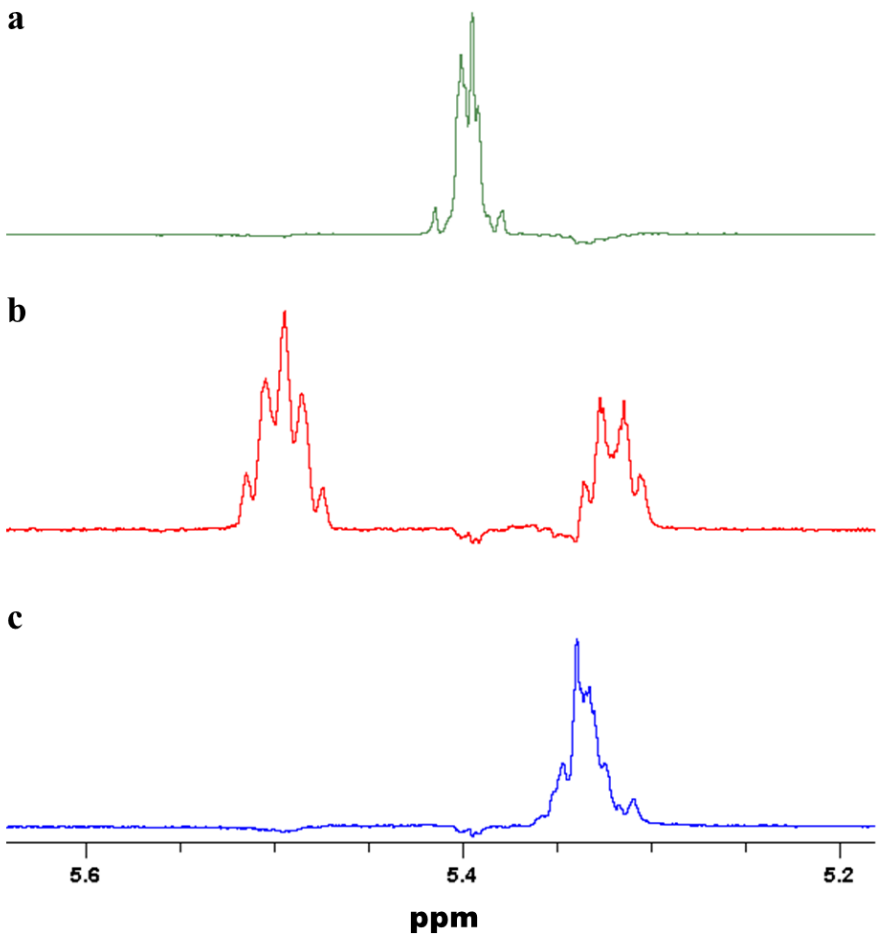

Supplementary Figure 51. H-5, H-6, H-8, H-9, H-14, and H-15 of trioxilin D_3. The peaks were confirmed with the selective TOCSY (mixing time = 40 ms). **(a)** H-14,15 irradiation on H-13. **(b)** H-9,8 irradiation on H-11. **(c)** H-5,6 irradiation on H-3.

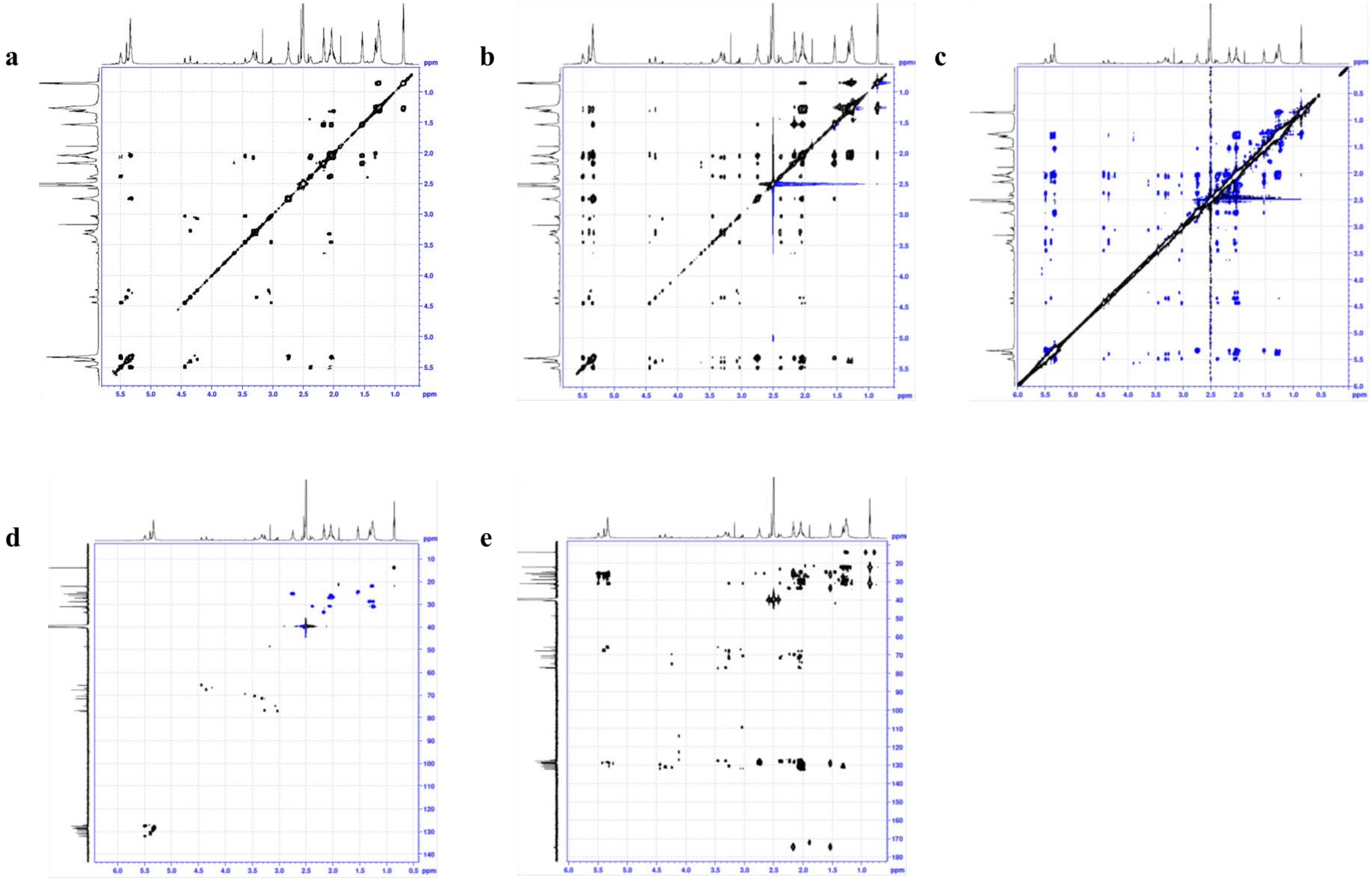

Supplementary Figure 52. 2D NMR of trioxilin D$_3$. (a) COSY. **(b)** TOCSY. **(c)** ROESY. **(d)** HSQC. **(e)** HMBC.

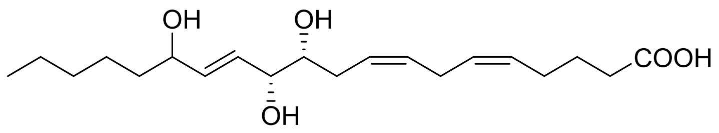

(5*Z*,8*Z*,11*R*,12*R*,13*E*)-11,12,15-trihydroxyicosa-5,8,13-trienoic acid

Supplementary Figure 53. Structure of trioxilin E_3.

a

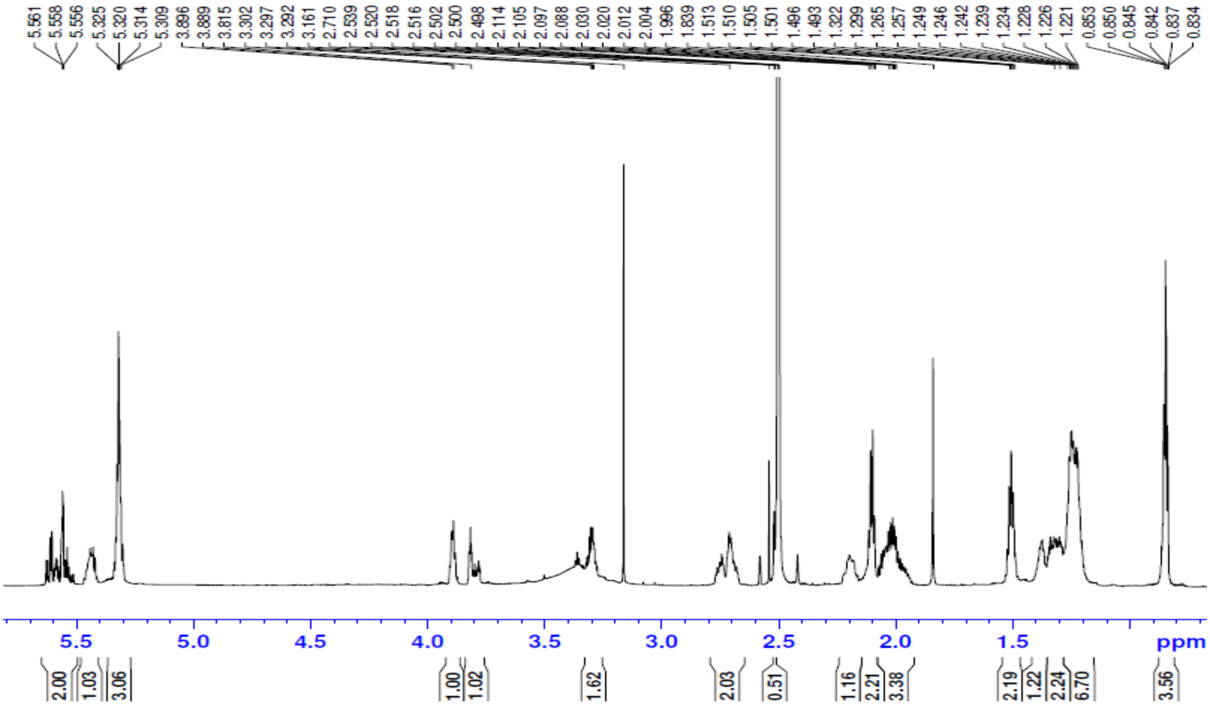

b

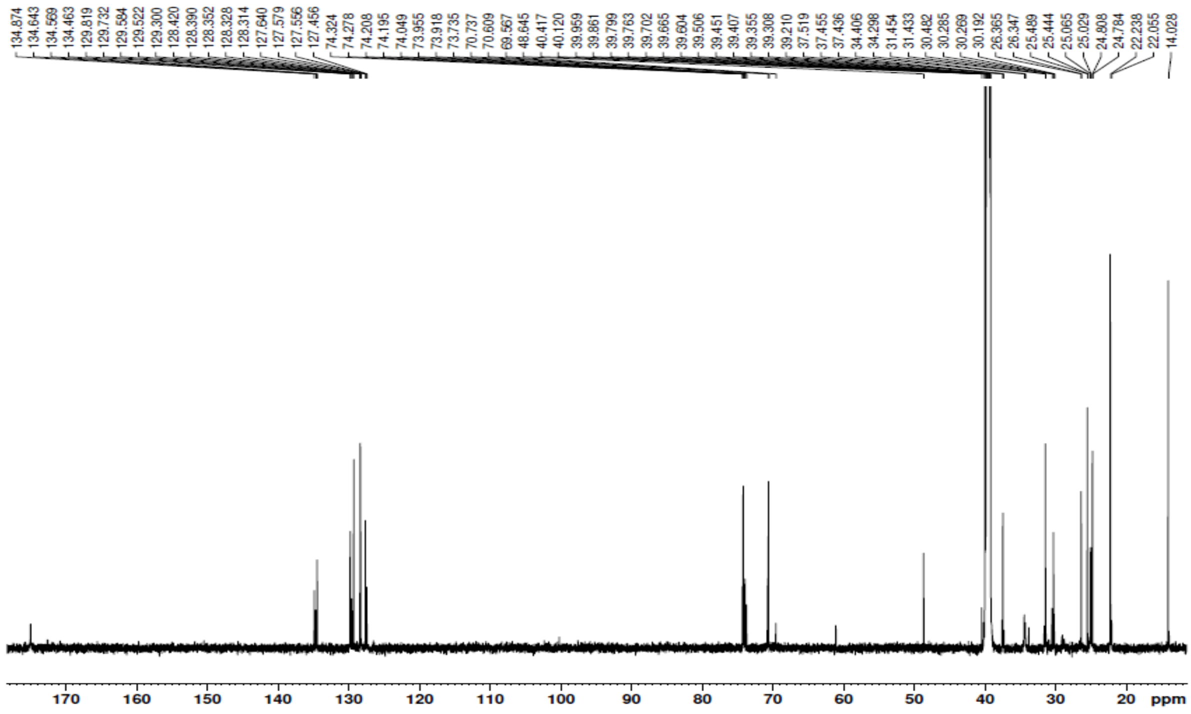

Supplementary Figure 54. 1D NMR data of trioxilin E_3. (a) 1H NMR peak of $TrXE_3$. **(b)** 13C NMR peak of $TrXE_3$.

a

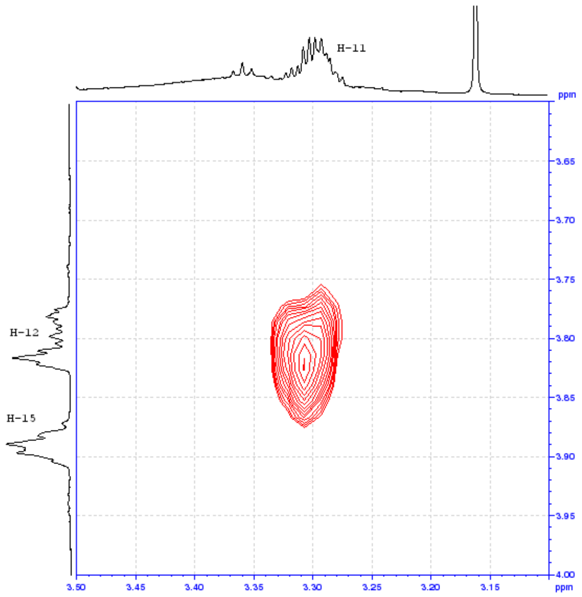

b

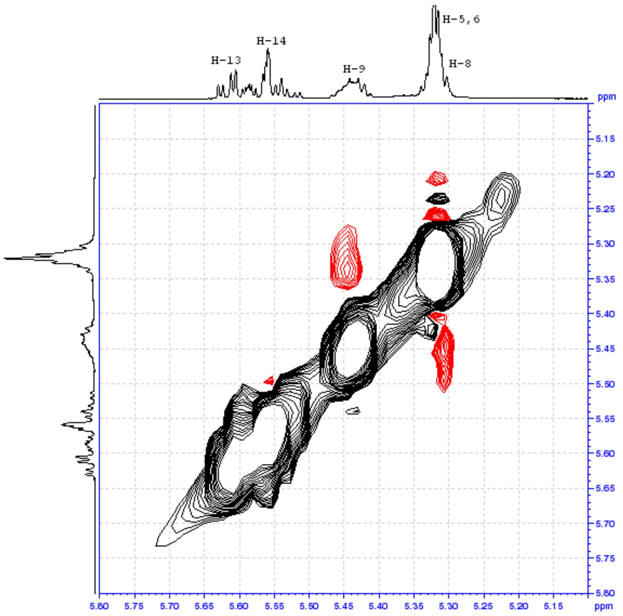

Supplementary Figure 55. ROESY NMR of trioxilin E_3. (a) ROE correlation of H-11, H-12, and H-13 of TrXE_3 at ROESY. **(b)** ROE correlation of between H-8 and H-9 of TrXE_3 at ROESY.

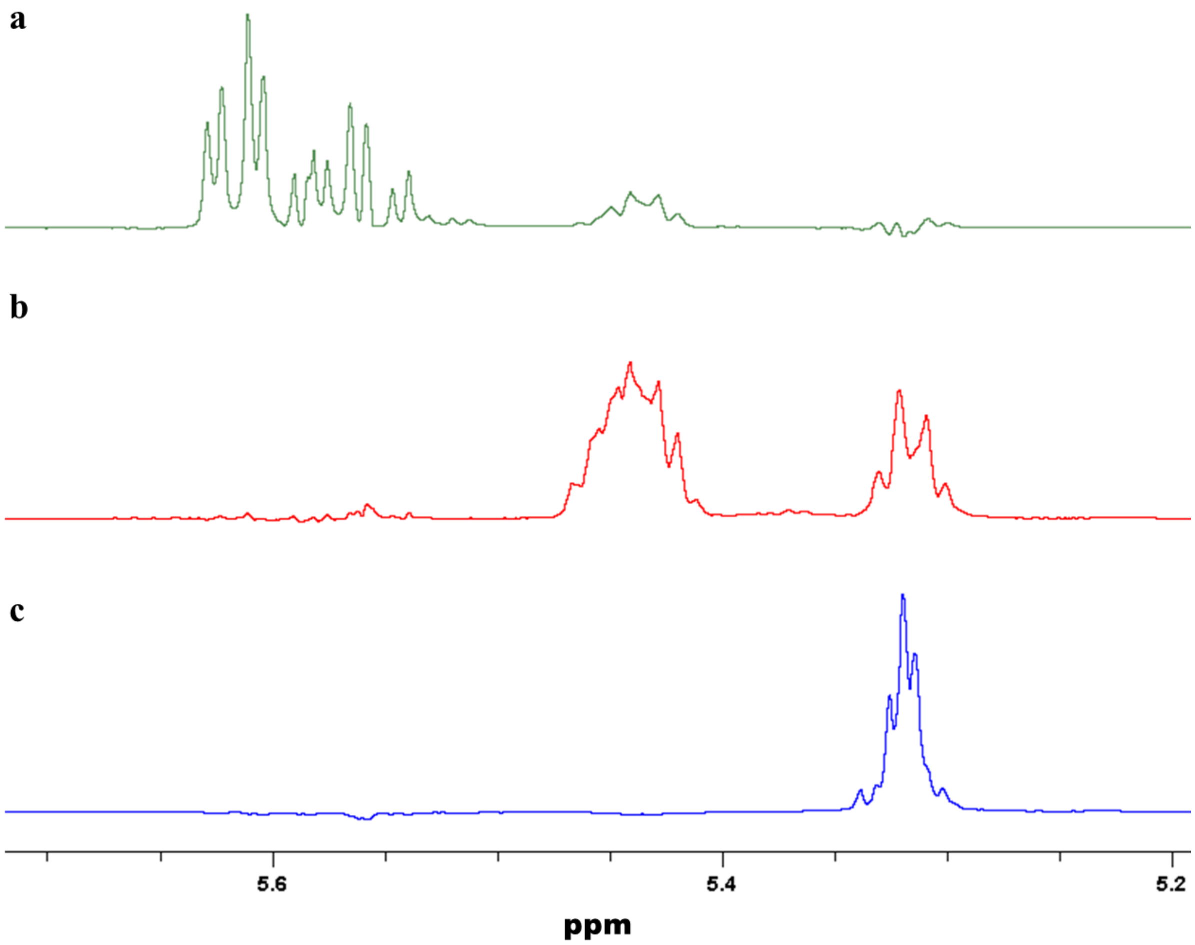

Supplementary Figure 56. H-5, H-6, H-8, H-9, H-14, and H-15 of trioxilin E_3. The peaks were confirmed with the selective TOCSY (mixing time = 80 ms). **(a)** H-13,14 irradiation on H-12. **(b)** H-9,8 irradiation on H-10. **(c)** H-5,6 irradiation on H-3.

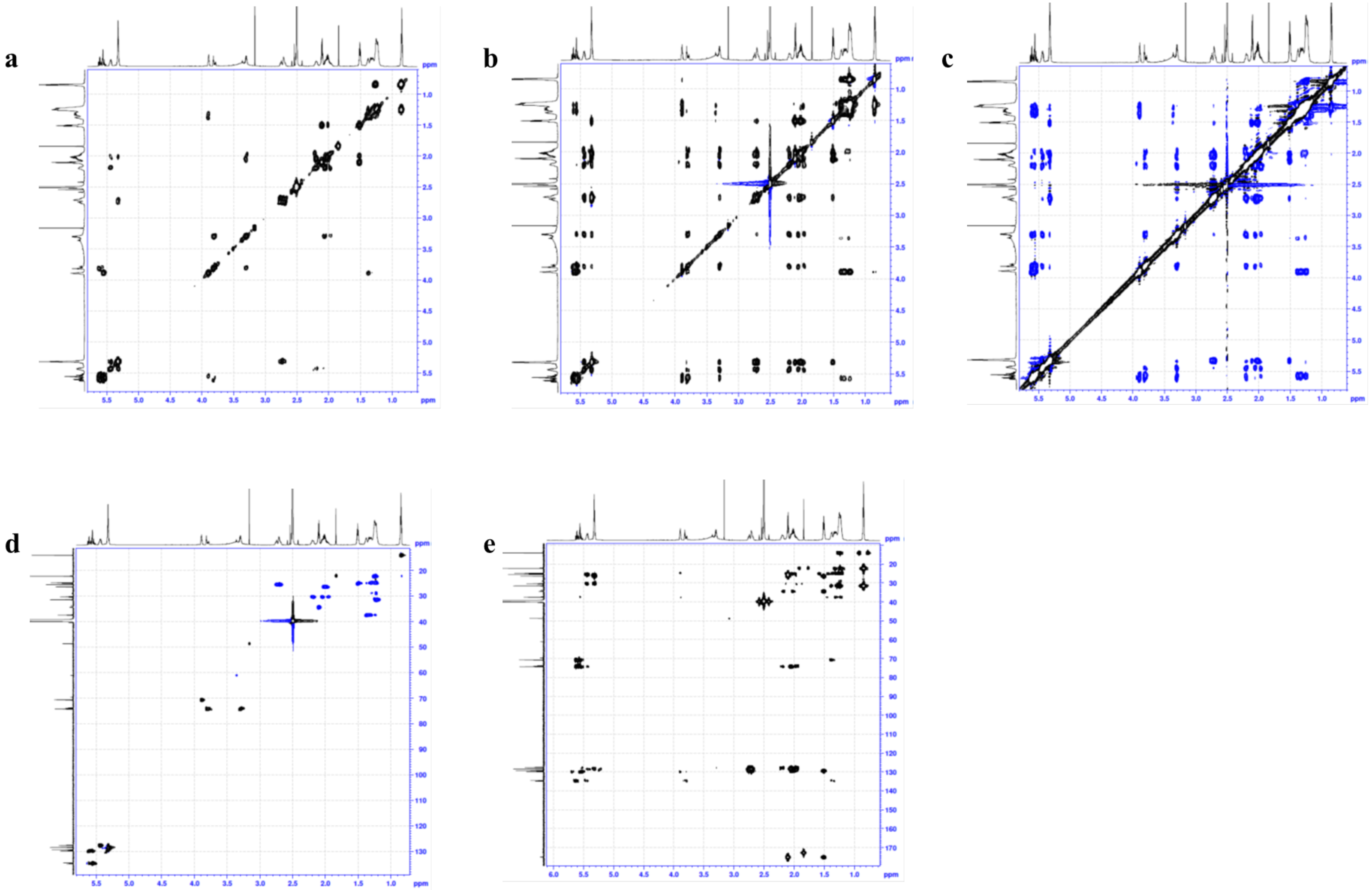

Supplementary Figure 57. 2**D NMR of trioxilin E$_3$. (a)** COSY. **(b)** TOCSY. **(c)** ROESY. **(d)** HSQC. **(e)** HMBC.

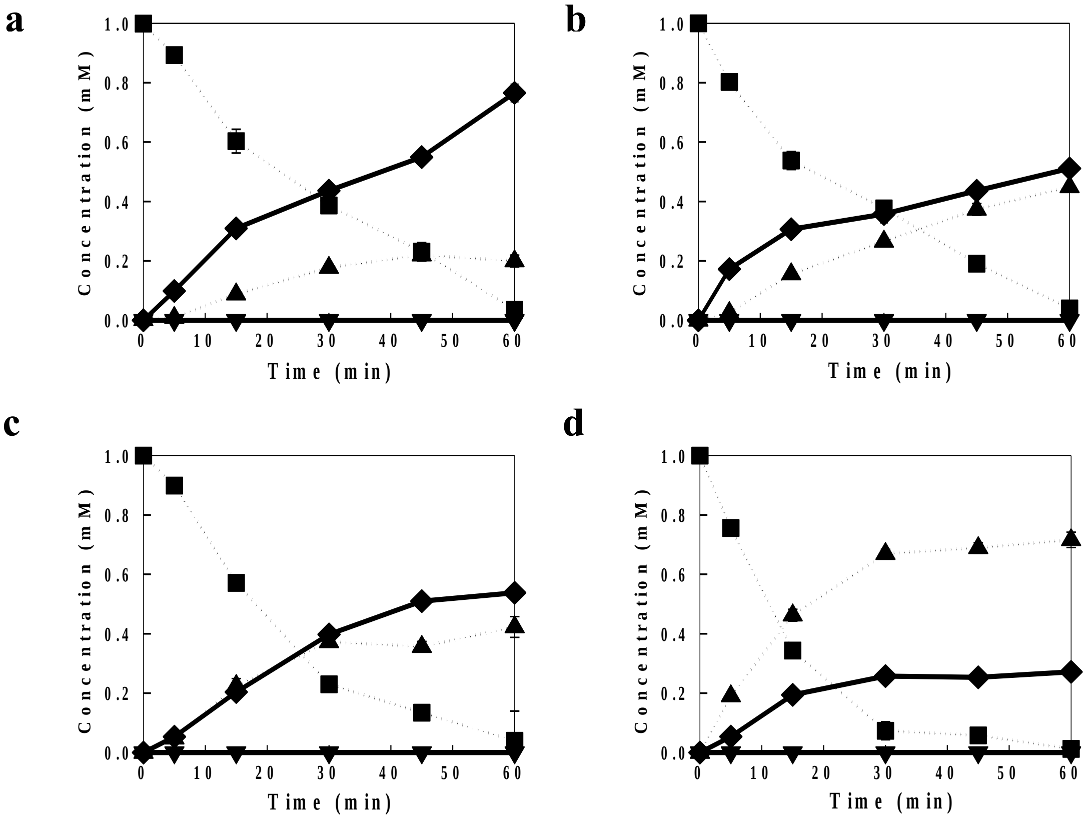

Supplementary Figure 58. Biotransformation of hydroperoxy fatty acids to hepoxilins by recombinant *Escherichia coli.* The reactions were performed in 50 mM 4-(2-hydroxyethyl)piperazinyl-1-propanesulphonic acid (EPPS) (pH 8.5) buffer containing 1 mM substrate and 3.6 g L^{-1} cells at 30°C for 60 min. **(a)** Biotransformation of 12-hydroperoxyeicosatetraenoic acid (12-HpETE) to HXB_3 by recombinant *E. coli* expressing 12-LOX. **(b)** Biotransformation of 12-hydroperoxyeicosapentaenoic acid (12-HpEPE) to HXB_4 by recombinant *E. coli* expressing 12-LOX. **(c)** Biotransformation of 14-hydroperoxydocosahexaenoic acid (14-HpDoHE) to HXB_5 by recombinant *E. coli* expressing 12-LOX. **(d)** Biotransformation of 11-HpETE to HXD_3 by recombinant *E. coli* expressing 11-LOX. Data represent the means of 3 separate experiments, and error bars represent the standard deviations. The symbols indicate HPFA (■), HFA (▲), HX (♦) and TrX (▼).

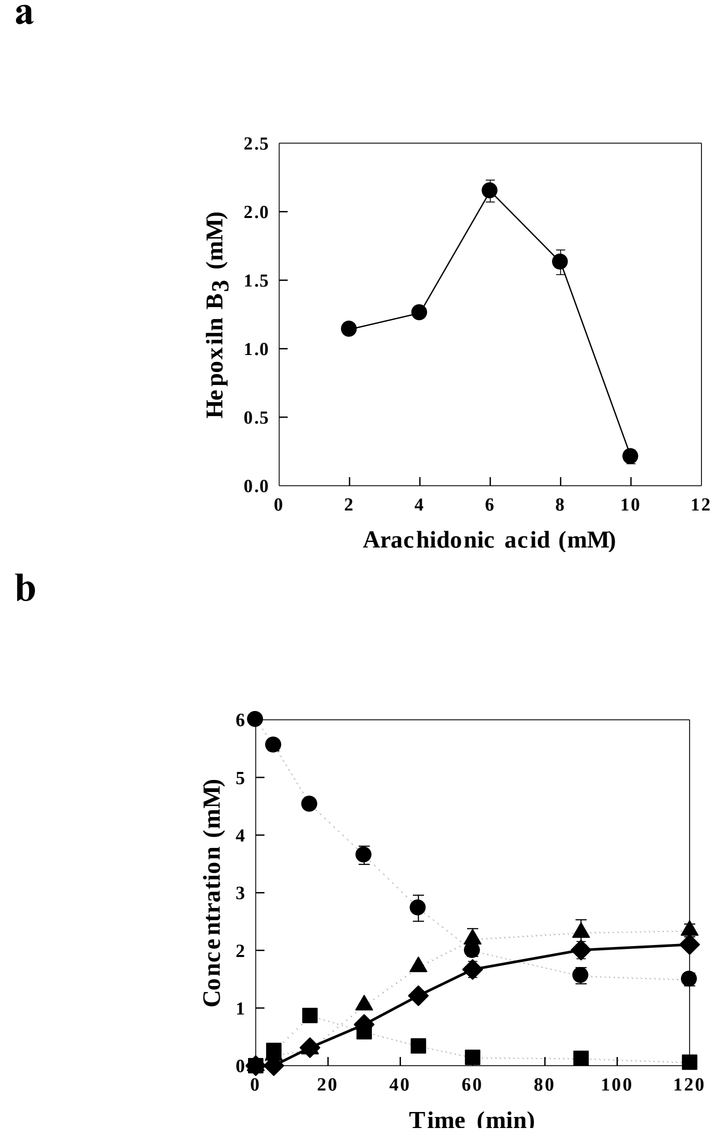

Supplementary Figure 59. Biotransformation of arachidonic acid to hepoxilin B_3 by recombinant *Escherichia coli.* **(a)** Effect of ARA concentration on HXB_3 production. The reactions by recombinant *E. coli* expressing 12-LOX were performed in 50 mM EPPS (pH 8.5) buffer containing 7.2 g L^{-1} cells by varying the concentration of ARA from 2 mM to 10 mM at 30°C for 30 min. **(b)** Time-course reactions for the conversion of ARA to HXB_3. The reactions by recombinant *E. coli* expressing 12-LOX were performed in 50 mM EPPS (pH 8.5) buffer containing 6 mM ARA and 14.4 g L^{-1} cells at 30°C for 120 min. Data represent the means of 3 separate experiments, and error bars represent the standard deviations. The symbols indicate ARA (●), 12-HpETE (■), 12-HETE (▲), HXB_3 (♦).

a

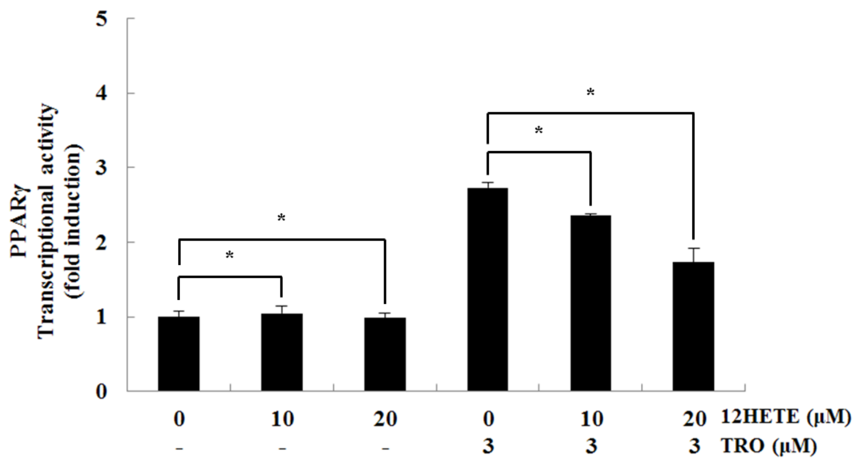

b

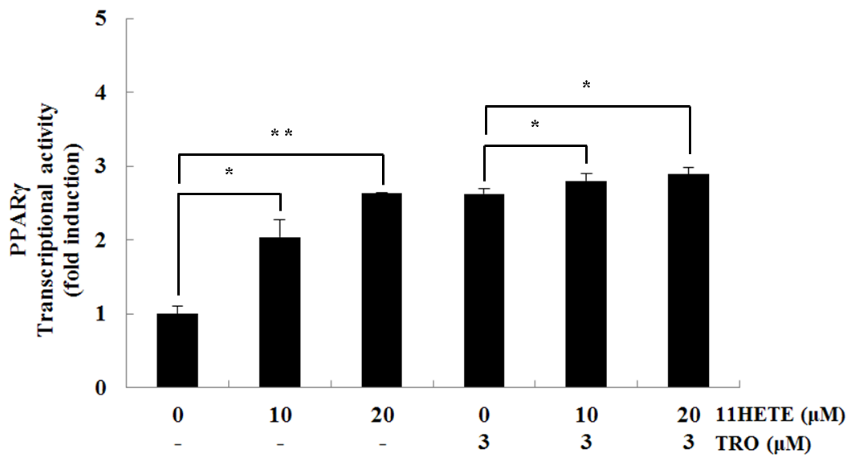

Supplementary Figure 60. Transcriptional activity of peroxisome proliferator-activated receptor gamma for hydroxy fatty acids. HEK-293 cells were cultured in a 24-well plate (1.0×10^5 cells per well). After 24 h of incubation, cells were transfected with plasmids expressing peroxisome proliferator-activated receptor gamma (PPARγ), PPAR response element (PPRE) × 3-thymidine kinase-luciferase reporter constructs, and the *Renilla* luciferase control vector pRL. After another 24 h, the cells were treated with HFAs and/or 3 μM troglitazone for 24 h. The cells were harvested, and the transcriptional activity of PPARγ was determined by a luciferase assay. **(a)** 12-HETE. **(b)** 11-HETE. Data represent the means of 3 separate experiments, and error bars represent the standard deviations. *p*-values are based on *t*-test. $^{*}p < 0.05$, $^{**}p < 0.01$.

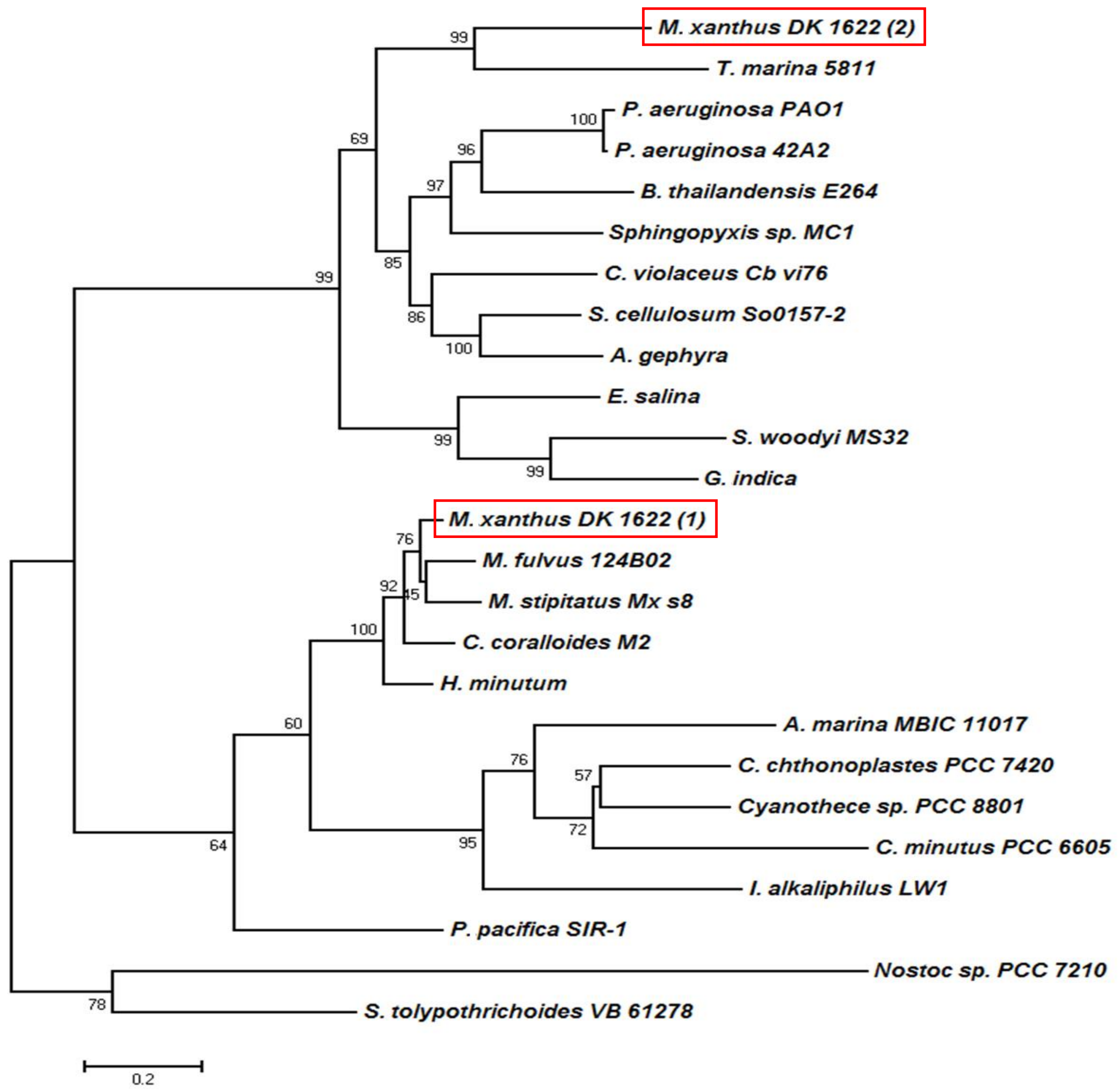

Supplementary Figure 61. Phylogenetic analysis of the nucleotide sequence of bacterial lipoxygenases. The phylogenetic analysis was performed using the neighbour-joining method. The optimal sum of the branch length is 9.822258 in the tree. The percentage of replicate trees in which the associated taxa clustered together in the bootstrap test (1,000 replicates) is shown next to the branches. The evolutionary distances are calculated using the maximum composite likelihood method and are expressed as the number of base substitutions per site. The analysis involved 25 nucleotide sequences. All positions containing gaps and missing data were eliminated. There were 1,039 positions in the final dataset. Evolutionary analyses were conducted in MEGA6. Accession numbers of LOXs and full names of strains are included in **Supplementary Table 15**.

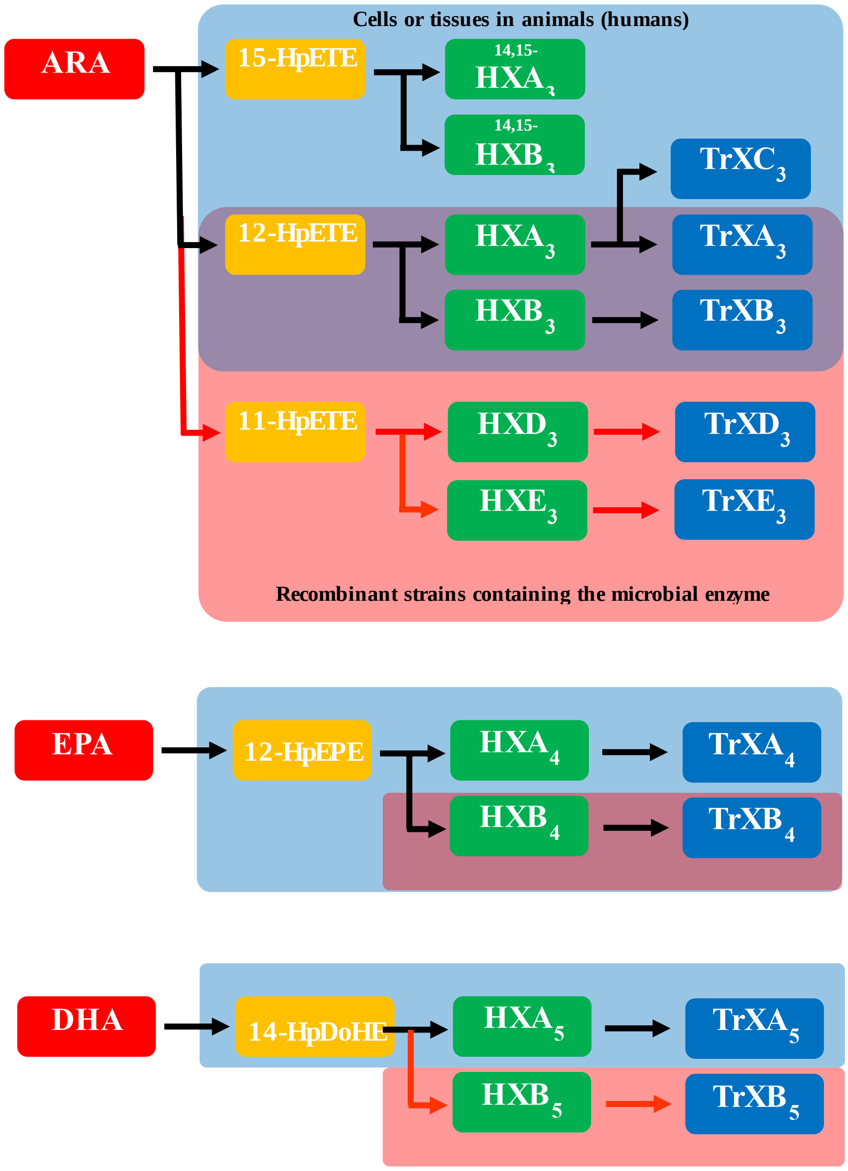

Supplementary Figure 62. Biosynthetic pathways for trioxilins in this study and published reports. The symbols indicate the reported pathways (blue box and black line) and new pathways identified in this study (red box and red line).

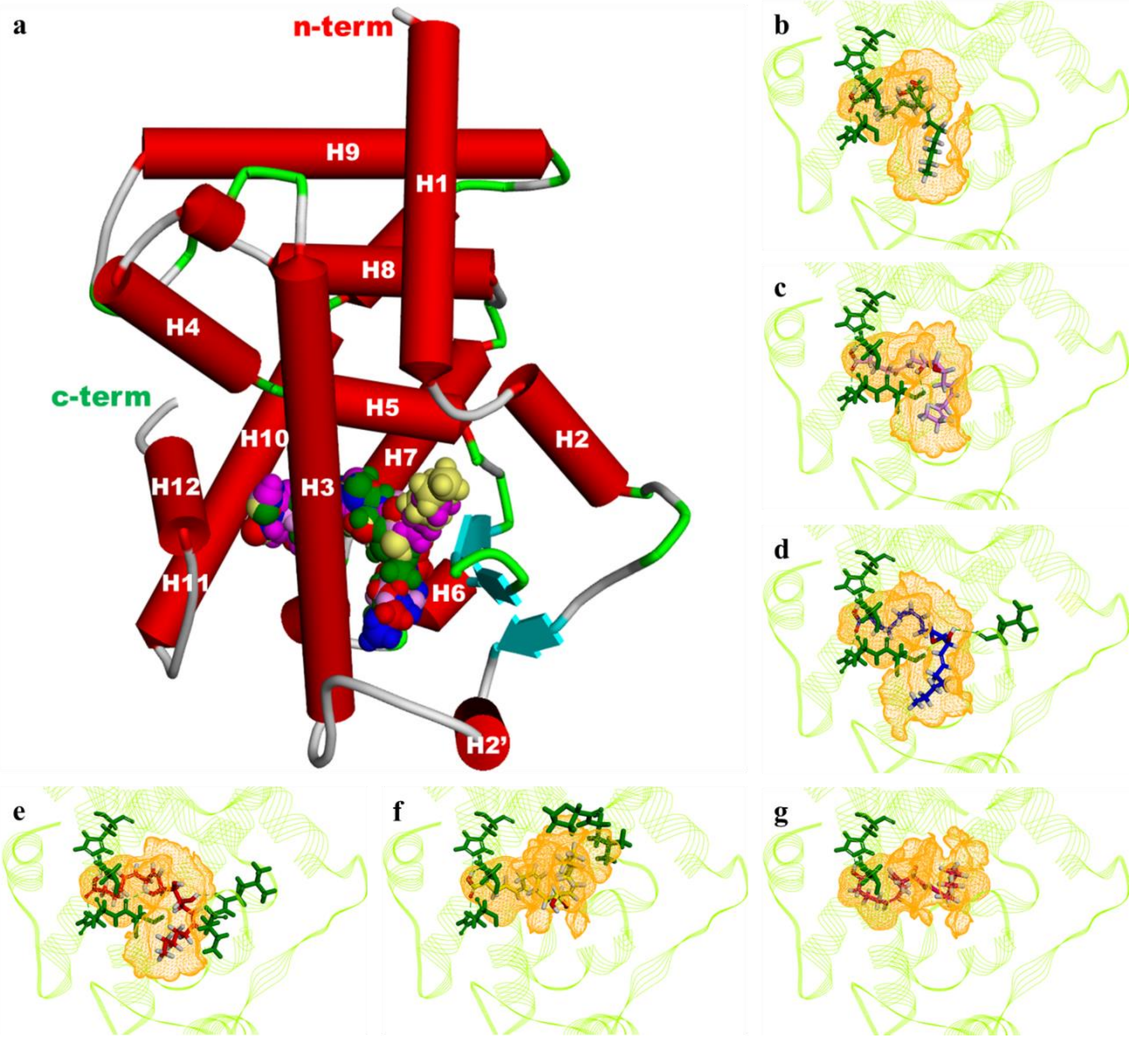

Supplementary Figure 63. Molecular docking of 6 compounds with ligand-binding domain of human peroxisome proliferator-activated receptor gamma . (A) Docking pose of 6 compounds with LBD of human peroxisome proliferator-activated receptor gamma (PPARγ), **(b)** HXB_3, **(c)** HXB_4, **(d)** HXD_3, **(e)** $TrXB_3$, **(f)** $TrXD_3$, and **(g)** 11-HETE. Docking studies to the structure of human PPAR**γ** (hPPAR**γ**) [PDB 2PRG] were performed in a CDOCKER module of Discovery Studio 4.1. The structure in orange net is show a hydrophobic pocket, and the amino acids with green of hPPAR**γ** show the residues of hydrogen bonding interaction with molecules. HXB_3, HXB_4, HXD_3, $TrXB_3$, $TrXD_3$, and 11-HETE are displayed in color with green, pink, blue, red, yellow, and purple, respectively.

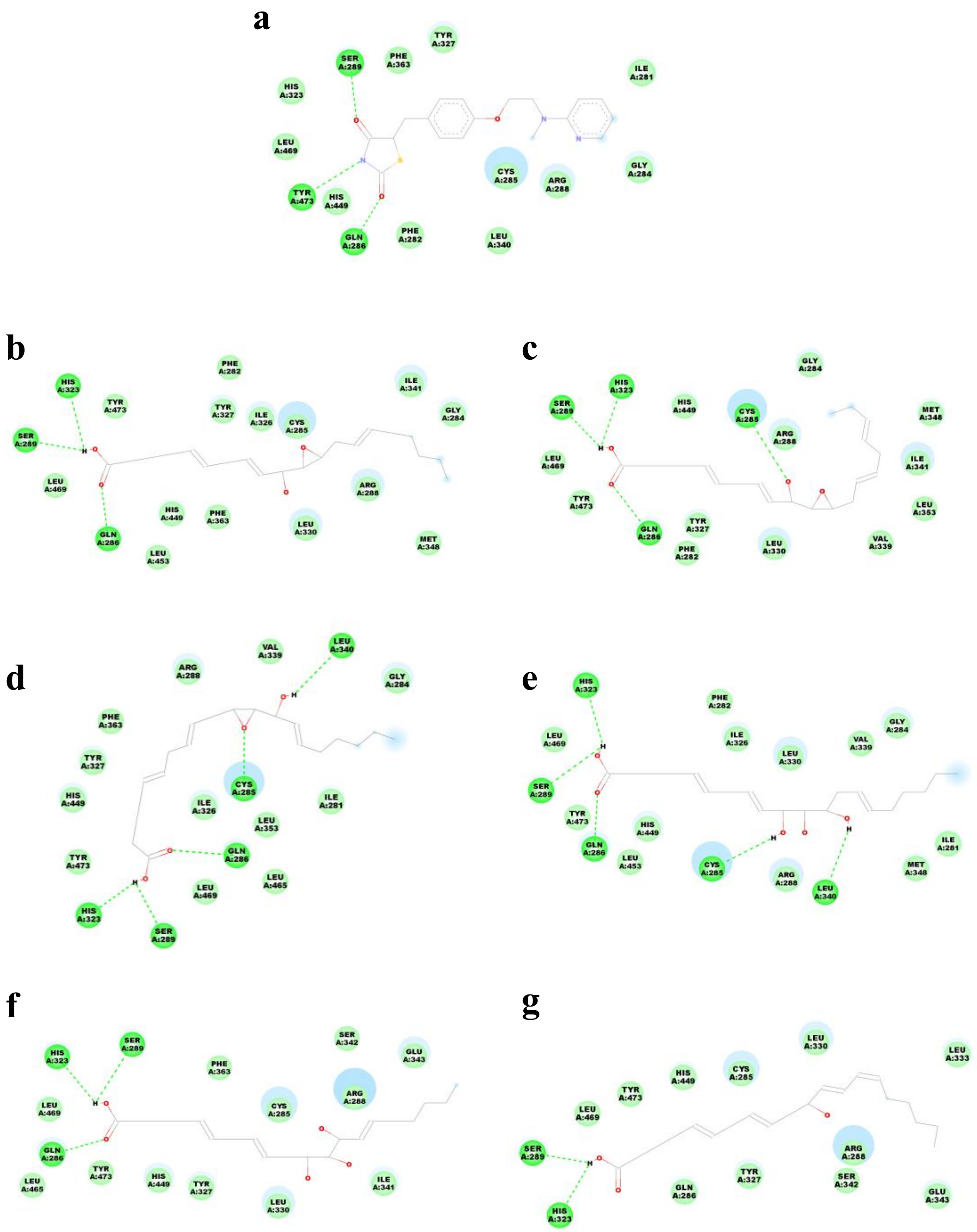

Supplementary Figure 64. 2D pictures of ligand interaction with human peroxisome proliferator-activated receptor gamma. The crystal structure of human PPARγ was used 2PGR (PDB). **(a)** Rosiglitazone. **(b)** HXB_3. **(c)** HXB_4. **(d)** HXD_3. **(e)** $TrXB_3$. **(f)** $TrXD_3$. **(g)** 11-HETE. Residues around ligand molecules represent green ball. Hydrogen bonds represented green dashed line. Blue circles show solvent accessible surface.

a

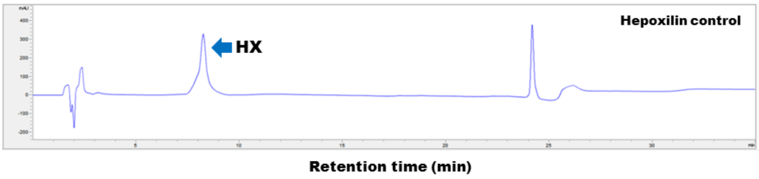

b

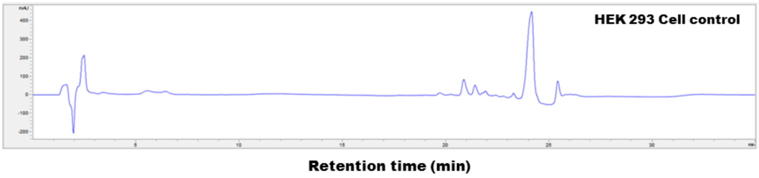

c

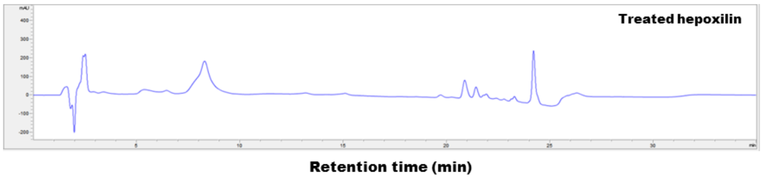

d

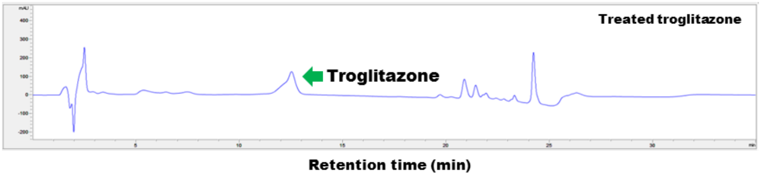

e

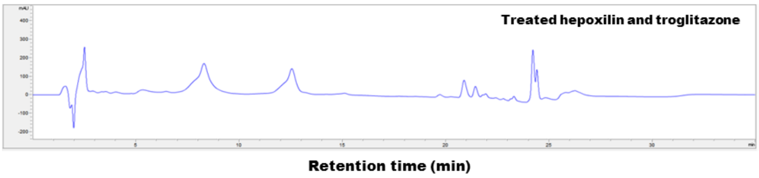

Supplementary Figure 65. HPLC analysis for the broth of HEK 293 cells with lipid mediator and/or troglitazone. HEK 293 cells was cultured for 24 h and the broth was analysed. **(a)** Treated HX without HEK 293 cells. **(b)** Only HEK 293 cells. **(c)** Treated HX with HEK 293 cells. **(d)** Treated troglitazone (TRO) with HEK 293 cells. **(e)** Treated HX and TRO with HEK 293 cells. The lipid mediators tested were not metabolized by HEK 293 cells.

a
b
c

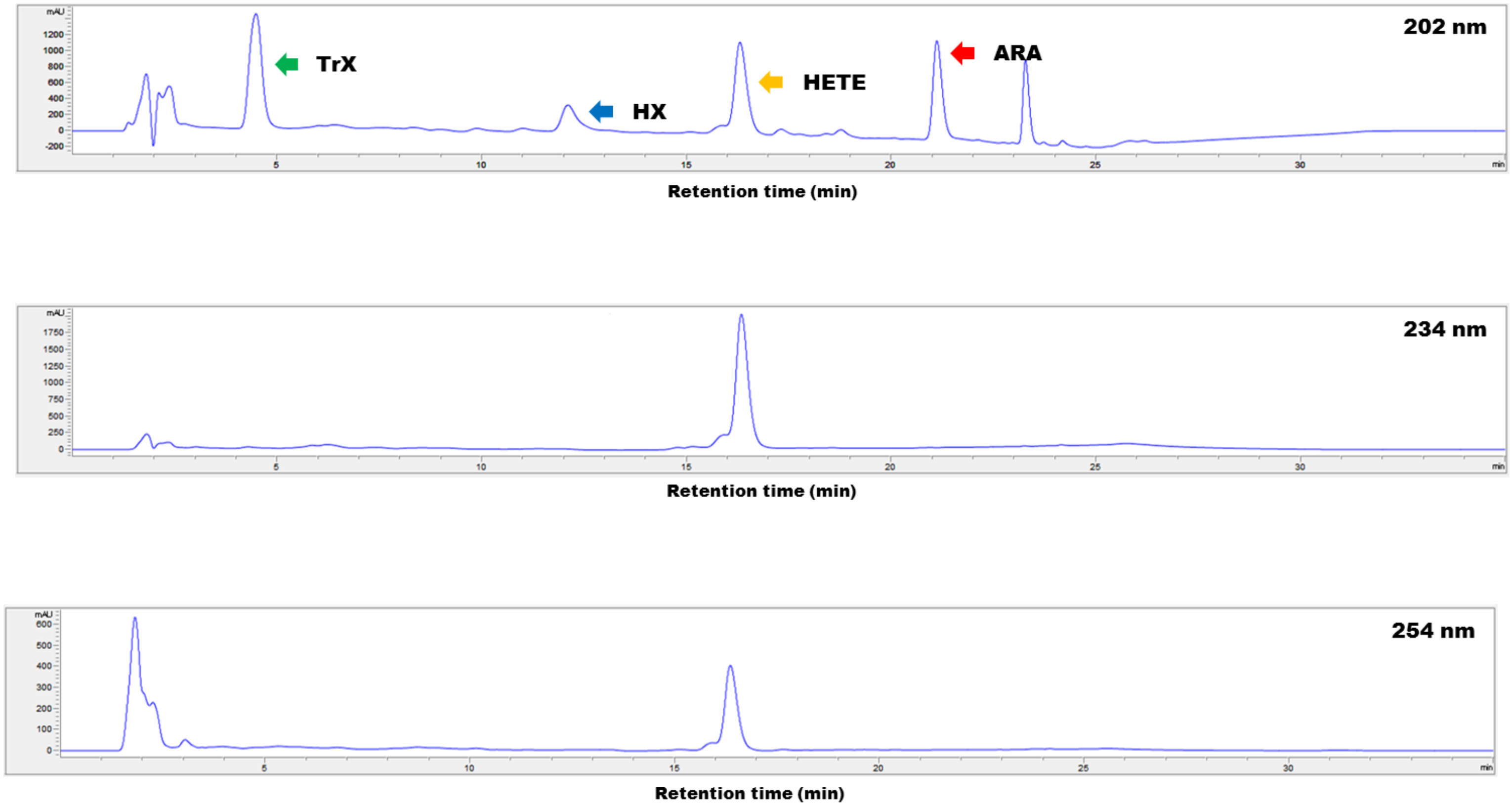

Supplementary Figure 66. Different wavelengths analysis of metabolites using HPLC. The metabolites were produced from ARA by recombinant *E. coli* expressing 12-LOX and EH from *M. xanthus*. **(a)** 202 nm, **(b)** 234 nm, and **(c)** 254 nm.

a

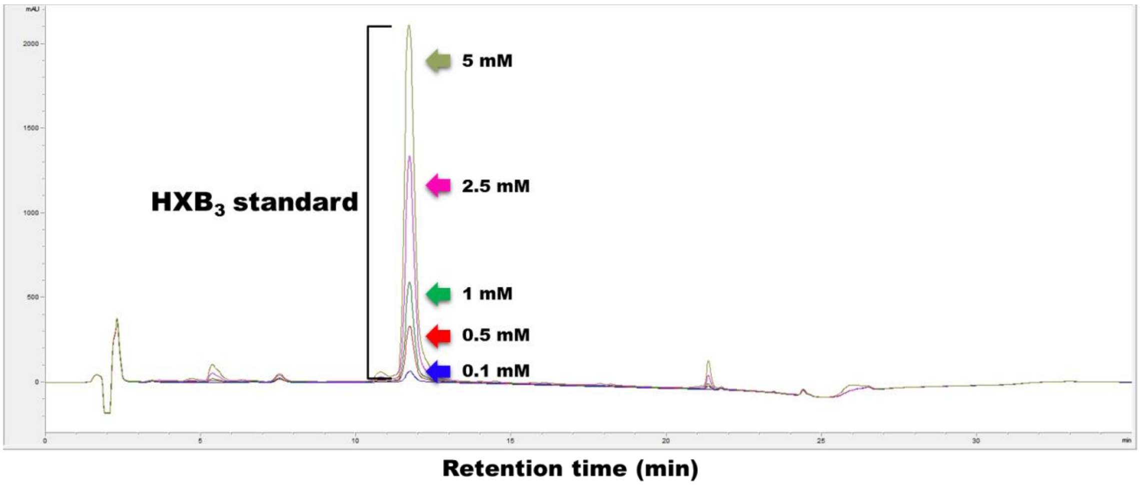

b

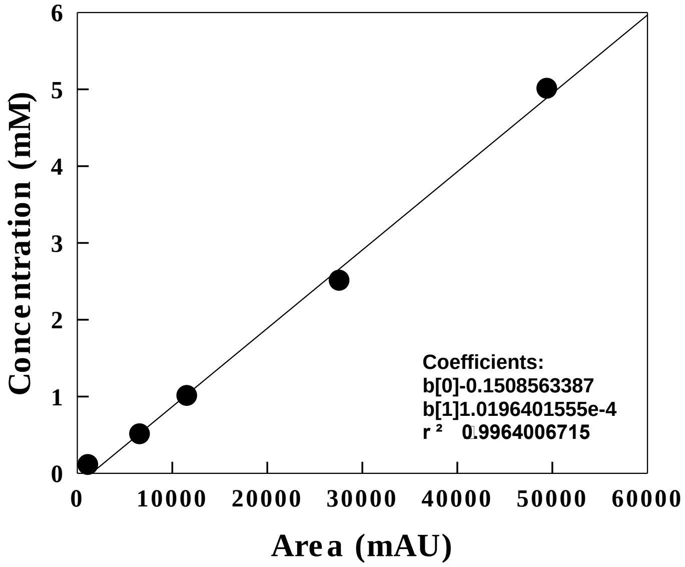

Supplementary Figure 67. Determination for the concentration of hepoxilin B_3 by HPLC. (a) HPLC analysis of HXB_3 standard at each concentration ranging from 0.1 to 5 mM. **(b)** Calibration curve of HXB_3 standard between area and concentration.

Supplementary Table 1. Abbreviations used in the study.

ARA	Arachidonic acid
EPA	Eicosapentaenoic acid
DHA	Docosahexaenoic acid
HX	Hepoxilin
TrX	Trioxilin
PG	Prostaglandin
LT	Leukotriene
LX	Lipoxin
HFA	Hydroxy fatty acid
HPFA	Hydro-peroxy fatty acid
EHFA	Epoxy-hydroxy fatty acid
HETE	Hydroxyeicosatetraenoic acid
HpETE	Hydroperoxyeicosatetraenoic acid
HEPE	Hydroxypentaenoic acid
HpEPE	Hydroperoxypentaenoic acid
HDoHE	Hydroxydocosahexaenoic acid
HpDoHE	Hydroperoxydocosahexaenoic acid
LOX	Lipoxygenase
COX	Cyclooxygenase
EH	Epoxide hydrolase
LB	Luria-Bertani
EPPS	4-(2-Hydroxyethyl)piperazinyl-1-propanesulfonic acid
HEK	human embryonic kidney
PPAR	Peroxisome proliferator activated receptor
PPRE	PPAR response element
TMZ	Thiazolidinedione
TRO	Troglitazone
LBD	Ligand binding domain
NMR	Nuclear magnetic resonance
HPLC	High performance liquid chromatography
LC-MS	Liquid chromatography-mass spectrometry
MAPS	Metabolites and pathways strategy

Supplementary Table 2. Classification and chemical names of hepoxilins and trioxilins.

Type I	Type II	Product	Chemical name	Reference
A	HX	HXA_3	8-Hydroxy-11,12-epoxyeicosa-5,9,14-trienoic acid	[1]
		HXA_4	8-Hydroxy-11,12-epoxyeicosa-5,10,14,17-tetraenoic acid	[2]
		HXA_5	10-Hydroxy-13,14-epoxydocosa-4,7,11,16,19-pentaenoic acid	[3]
		14,15-HXA_3	11-Hydroxy-14,15-epoxyeicosa-5,8,12-trienoic acid	[4]
	TrX	$TrXA_3$	8,11,12-Trihydroxyeicosa-5,9,14-trienoic acid	[1]
		$TrXA_4$	8,11,12-Trihydroxyeicosa-5,10,14,17-tetraenoic acid	[2]
		$TrXA_5$	10,13,14-Trihydroxydocosa-4,7,11,16,19-pentaenoic acid	[3]
B	HX	HXB_3	10-Hydroxy-11,12-epoxyeicosa-5,8,14-trienoic acid	This study
		HXB_4	10-Hydroxy-11,12-epoxyeicosa-5,8,14,17-tetraenoic acid	This study
		HXB_5	12-Hydroxy-13,14-epoxydocosa-4,7,10,16,19-pentaenoic acid	This study
		14,15-HXB_3	13-Hydroxy-14,15-epoxyeicosa-5,8,11-trienoic acid	[4]
	TrX	$TrXB_3$	10,11,12-Trihydroxyeicosa-5,8,14-trienoic acid	This study
		$TrXB_4$	10,11,12-Trihydroxyeicosa-5,8,14,17-tetraenoic acid	This study
		$TrXB_5$	12,13,14-Trihydroxydocosa-4,7,10,16,19-pentaenoic acid	This study
C	TrX	$TrXC_3$	8,9,12-Trihydroxyeicosa-5,10,14-trienoic acid	[5]
D	HX	HXD_3	13-Hydroxy-11,12-epoxyeicosa-5,8,14-trienoic acid	This study
	TrX	$TrXD_3$	11,12,13-Trihydroxyeicosa-5,8,14-trienoic acid	This study
E	HX	HXE_3	15-Hydroxy-11,12-epoxyeicosa-5,8,13-trienoic acid	This study
	TrX	$TrXE_3$	11,12,15-Trihydroxyeicosa-5,8,13-trienoic acid	This study

Supplementary Table 3. Molecular formulae of arachidonic acid and its metabolites by *Myxococcus xanthus*.

Metabolite No	*m/z*	Retention time (min)	MS/MS fragment masses[1]	Molecular formula	Suggested compounds[2] (Formal name)
1	303.2	10.55	80.2, 177.1, 205.3, 259.2, 285.1, 303.2	$C_{20}H_{31}O_2$	ARA
2	301.2	8.47	149.2, 203.2, 229.1, 257.2, 301.2	$C_{20}H_{29}O_2$	EPA
3	319.2	9.09	167.3, 195.3, 275.2, 301.1, 319.2	$C_{20}H_{32}O_3$	11-HETE
4	319.2	9.56	163.1, 179.2, 275.3, 301.1, 319.2	$C_{20}H_{32}O_3$	12-HETE
5	319.3	8.36	175.2, 219.1, 275.2, 301.2, 319.3	$C_{20}H_{32}O_3$	15-HETE
6	335.4	7.57	153.2, 183.2, 195.2, 263.3, 317.2, 335.4	$C_{20}H_{32}O_4$	HXB_3
7	367.5	7.44	187.1, 235.2, 289.3, 315.4, 331.4, 333.3, 367.5	$C_{20}H_{32}O_6$	PGG_2
8	351.2	6.91	189.2, 233.1, 271.2, 299.3, 315.3, 333.3, 351.2	$C_{20}H_{32}O_5$	PGH_2
9	335.2	8.12	97.1, 127.3, 167.2, 209.2, 317.2, 335.2	$C_{20}H_{32}O_4$	No match[3] (HX analogue)
10	353.2	6.17	139.3, 153.1, 201.2, 242.1, 335.1, 353.1	$C_{20}H_{33}O_5$	No match (TrX analogue)
11	351.2	6.31	113.2, 175.1, 189.1, 235.2, 271.2, 315.2, 351.2	$C_{20}H_{32}O_5$	No match (PGE_2, PGD_2, TXA_2)
12	333.2	5.89	113.2, 175.8, 189.2, 271.3, 315.1, 333.2	$C_{20}H_{29}O_4$	No match (PGA_2, PGB_2, PGJ_2, 12-HpEPE)

[1]Metabolites were analysed by HPLC and LC-MS/MS at 202 nm.

[2]Products were compared with the references in LIPID MAPS database (http://lipidmaps.org).

[3]No match means that the compound is not correctly identified.

Supplementary Table 4. Candidate biosynthetic genes of lipid mediators in *Myxococcus xanthus* genome.

Target enzyme[1]	Template gene	Entry	Protein name	Gene name	Identity (%)[2]	Length (bp)	Expression	Activity
LOX	P18054 (ALOX12)	Q1DBH9	Lipoxygenase family protein	*MXAN_1744*	30.0	2028	○	○
		Q1DBH8	Uncharacterized protein	*MXAN_1745*	15.2	2148	○	○
EH	P34913 (EPHX2)	Q1DBS7	Putative epoxide hydrolase	*MXAN_1644*	20.9	957	○	○
	P09960 (LTA4H)	Q1D232	Peptidase M1	*MXAN_5137*	36.4	1755	○	×
COX	P35354 (PTGS2)	Q1D1V4	Peroxidase family protein	*MXAN_5217*	18.5	1995	○	○
TXA synthase	P24557 (TBXAS1)	Q1DEH2	Cytochrome P450 family protein	*MXAN_0683*	21.2	1404	○	×
		Q1D9Z9	Cytochrome P450 family protein	*MXAN_2304*	20.1	1377	○	×
PGD synthase	O60760 (HPGDS)	Q1D6B3	Putative glutathione S-transferase	*MXAN_3623*	24.3	687	○	×

[1]Genes were selected based on sequence alignments with human corresponding enzymes.

[2]Identity means the sequence similarity with human corresponding enzymes.

Supplementary Table 5. Specific activities of enzymes in *Myxococcus xanthus*.

Enzyme	Substrate	Product	Specific activity (μmol min^{-1} mg^{-1})[1]
12-LOX (*MXAN 1745*)	ARA	12-HpETE	605 ± 1.7
		HXB_3	59.6 ± 0.2
	EPA	12-HpEPE	134 ± 3.9
		HXB_4	35.6 ± 0.1
	DHA	14-HpDoHE	112 ± 2.7
		HXB_5	34.3 ± 0.1
	12-HpETE	HXB_3	74.1 ± 0.1
	12-HpEPE	HXB_4	42.6 ± 0.2
	14-HpDoHE	HXB_5	66.8 ± 0.3
11-LOX (*MXAN 1744*)	ARA	11-HpETE	489 ± 1.8
		HXD_3	29.3 ± 0.1
	EPA	11-HpEPE	136 ± 3.9
	DHA	14-HpDoHE	91.6 ± 3.4
	11-HpETE	HXD_3	46.8 ± 0.6
EH (*MXAN 1644*)	HXB_3	$TrXB_3$	1,403 ± 5.4
	HXB_4	$TrXB_4$	985 ± 12.9
	HXB_5	$TrXB_5$	793 ± 8.4
	HXD_3	$TrXD_3$	1,158 ± 3.8

[1]Data represent the means of three separate experiments, and ± represent the standard deviations.

Supplementary Table 6. 1D NMR data of hepoxilin B_3.

#	1H (δ)	multiplet	J (Hz)	Protons	13C (δ)
1					174.3
2	2.20	t	6.44	2H	33.0
3	1.54	tt	7.11	2H	24.4
			6.44		
4	2.03	td	7.11	2H	26.1
			6.52		
5	5.34	m		1H	129.4
6	5.36	m		1H	128.1
7	2.77	m		2H	25.8
8	5.37	m		1H	129.5
9	5.36	m		1H	129.5
10	4.07	dd	7.65, 6.03	1H	67.1
11	2.71	dd	6.03, 2.17	1H	60.7
12	2.82	td	5.43, 2.17	1H	54.7
13	2.29	m		1H	29.0
	2.17			1H	
14	5.33	m		1H	123.8
15	5.47	dt	10.89, 5.43	1H	132.3
16	1.96	dt	7.10, 7.05	2H	26.7
17	1.30	m		2H	28.9
18	1.25	m		2H	30.8
19	1.25	m		2H	21.9
20	0.86	t	6.88	3H	13.9

Supplementary Table 7. 1D NMR data of hepoxilin B_4.

#	1H (δ)	multiplet	J (Hz)	Protons	13C (δ)
1					174.3
2	2.20	t	7.41	2H	33.1
3	1.54	tt	7.41, 7.02	2H	24.4
4	2.04	dt	6.55, 7.02	2H	26.1
5	5.36	m		1H	129.4
6	5.33	m		1H	128.1
7	2.77	m		2H	25.8
8	5.36	m		1H	129.5
9	5.36	m		1H	129.5
10	4.08	dd	7.63, 6.00	1H	67.1
11	2.72	dd	6.00, 2.20	1H	60.7
12	2.84	td	8.15, 2.20	1H	54.5
13	2.32	m		1H	29.0
	2.21	m		1H	
14	5.33	m		1H	124.0
15	5.44	m		1H	130.4
16	2.74	dd	6.68, 6.62	1H	25.2
17	5.27	m		1H	126.8
18	5.36	m		1H	131.6
19	2.03	dt	6.95, 7.53	2H	20.0
20	0.91	t	7.53	3H	14.1

Supplementary Table 8. 1D NMR data of hepoxilin B_5.

#	1H (δ)	multiplet	J (Hz)	Protons	13C (δ)
1					174.2
2	2.20	t	6.93	2H	34.2
3	2.24	dt	6.93	2H	22.7
4	5.38	m		1H	128.9
5	5.39	m		1H	128.3
6	2.78	m		2H	25.2
7	5.32	m		1H	128.1
8	5.30	m		1H	129.7
9	2.78	m		2H	25.2
10	5.39	m		1H	129.6
11	5.36	m		1H	129.4
12	4.09	dd	5.95	1H	67.1
			8.34		
13	2.72	dd	2.02	1H	60.7
			5.95		
14	2.84	m		1H	54.8
15	2.32	m		1H	29.0
	2.21	m		1H	
16	5.33	m		1H	124.0
17	5.43	m		1H	130.4
18	2.74	t	7.16	2H	25.2
19	5.27	m		1H	126.9
20	5.36	m		1H	131.6
21	2.02	dt	7.03	2H	20.0
			7.52		
22	0.92	t	7.52	3H	14.1

Supplementary Table 9. 1D NMR data of hepoxilin D_3.

#	1H (δ)	multiplet	J (Hz)	Protons	13C (δ)
1					174.3
2	2.20	t	6.38	2H	33.0
3	1.54	tt	6.38	2H	24.4
			7.08		
4	2.04	dt	7.08	2H	26.0
			5.74		
5	5.33	m		1H	129.2
6	5.33	m		1H	128.1
7	2.74	dd	5.72, 4.17	2H	25.2
8	5.42	m		1H	130.3
9	5.33	m		1H	124.0
10	2.33	m		1H	29.2
	2.20	m		1H	
11	2.81	dt	2.15, 5.42	1H	54.6
12	2.70	dd	2.15, 6.11	1H	60.8
13	4.00	ddd	8.63, 6.11	1H	67.2
14	5.43	m		1H	131.5
15	5.33	m		1H	129.6
16	1.99	dt	4.36, 6.84	2H	26.02
17	1.32-1.2	m		2H	31.09
18	1.32-1.2	m		2H	30.83
19	1.32-1.2	m		2H	21.93
20	0.85	t	7.04	3H	13.82

Supplementary Table 10. 1D NMR data of trioxilin B_3.

#	1H (δ)	multiplet	J (Hz)	Protons	13C (δ)
1					174.5
2	2.19	t	7.35	2H	33.7
3	1.54	tt	7.20, 7.35	2H	24.5
4	2.04	dt	7.20,	2H	26.52
5	5.35	m		1H	128.4
6	5.34	m		1H	128.4
7	2.83	m		1H	25.7
	2.73			1H	
8	5.30	m		1H	127.8
9	5.51	m		1H	132.2
10	4.50	dd	8.75, 2.92	1H	65.7
11	3.03	dd	7.27, 2.92	1H	76.9
12	3.44	dd	7.27, 8.35	1H	70.5
13	2.35	m		1H	31.0
	2.04			1H	
14	5.48	m		1H	127.4
15	5.38	m		1H	130.2
16	1.98	m		2H	26.6
17	1.30	m		2H	29.0
18	1.25	m		2H	30.9
19	1.27	m		2H	22.0
20	0.86	t	6.82	3H	13.9

Supplementary Table 11. 1D NMR data of trioxilin B_4.

#	1H (δ)	multiplet	J (Hz)	Protons	13C (δ)
1					176.3
2	1.96	m		2H	35.8
3	1.49	m		2H	25.8
4	2.00	m		2H	26.62
5	5.32	m		1H	129.7
6	5.32	m		1H	127.5
7	2.98	dt	15.4, 7.32	1H	25.9
	2.87	dt	15.4, 7.32	1H	
8	5.31	m		1H	127.07
9	5.51	dd	9.49, 9.25	1H	131.9
10	4.63		8.74, 1.57	1H	64.4
11	2.89		8.16, 1.57	1H	77.5
12	3.47	ddd	8.38, 8.16, 2.77	1H	70.0
13	2.40	ddd	14.72,	1H	31.0
	2.02	m	6.94, 2.77	1H	
14	5.52	m		1H	128.2
15	5.31	m		1H	127.9
16	2.75	dd	5.6, 7.2	2H	25.4
17	5.29	m		1H	127.5
18	5.34	m		1H	131.2
19	2.03	dt	6.89, 7.52	2H	20.0
20	0.92	t	7.52	3H	14.2

Supplementary Table 12. 1D NMR data of trioxilin B_5.

#	1H (δ)	multiplet	J (Hz)	Protons	13C (δ)
1					174.3
2	2.21	t	6.45	2H	34.33
3	2.25	dt	6.45	2H	22.7
4	5.35	m		1H	128.7
5	5.33	m		1H	128.3
6	2.8	dd		2H	25.1
7	5.33	m		1H	128.0
8	5.33	m		1H	127.8
9	2.88	m		1H	25.7
	2.78			1H	
10	5.32	m		1H	
11	5.53	m		1H	132.1
12	4.53	dd	8.89, 2.25	1H	65.6
13	3.04	dd	7.49, 2.25	1H	76.9
14	3.47	ddd	2.74, 7.49, 8.19	1H	70.4
15	2.38	m		1H	31.0
	2.06			1H	
16	5.50	m		1H	127.6
17	5.33	m		1H	128.3
18	2.75	dd		2H	25.3
19	2.29	m		1H	127.4
20	5.34	m		1H	131.3
21	2.03	dt	6.82, 7.53	2H	20.0
22	0.92	t	7.53	3H	14.1

Supplementary Table 13. 1D NMR data of trioxilin D_3.

#	1H (δ)	multiplet	J (Hz)	Protons	13C (δ)
1					174.7
2	2.17	t	7.22	2H	33.5
3	1.53	tt	6.36, 7.22	2H	24.7
4	2.03	m		2H	26.1
5	5.33	m		1H	129.0
6	5.33	m		1H	128.6
7	2.74	m		2H	25.4
8	5.33	m		1H	128.3
9	5.49	m		1H	127.6
10	2.38	m		1H	30.9
	2.06	m		1H	
11	3.32	td	7.27, 2.97	1H	71.5
12	3.26	dd	4.62, 7.27	1H	76.7
13	4.35	dd	7.52, 4.62	1H	67.7
14	5.40	dd		1H	130.5
15	5.40	m		1H	131.1
16	2.07	m		1H	27.3
	2.01	m		1H	
17	1.31	m		2H	28.8
18	1.25	m		2H	31.0
19	1.27	m		2H	22.0
20	0.86	t	6.86	3H	13.9

Supplementary Table 14. 1D NMR data of trioxilin E_3.

#	1H (δ)	multiplet	J (Hz)	Protons	13C (δ)
1					175
2	2.10	t	7.03	2H	34.4
3	1.50	tt	6.04, 7.03	2H	25.1
4	2.02	m		2H	26.4
5	5.31	m		1H	129.3
6	5.31	m		1H	128.4
7	2.74	m		1H	25.5
	2.71	m		1H	
8	5.31	m		1H	128.4
9	5.44	m		1H	127.6
10	2.19	m		1H	30.3
	2.05	m		1H	
11	3.29	m	4.53	1H	74.2
12	3.81	dd	4.53, 5.36	1H	74.2
13	5.61	dd	15.78, 5.36	1H	129.8
14	5.55	dd	15.78, 6.23	1H	134.5
15	3.89	m		1H	70.6
16	1.37	m		1H	37.4
	1.33	m		1H	
17	1.30	m		1H	24.8
	1.23	m		1H	
18	1.22	m		2H	31.4
19	1.25	m		2H	22.2
20	0.85	t	7.09	3H	14.0

Supplementary Table 15. Bacteria containing lipoxygenase and their GenBank accession numbers.

No	Organism[1]	Taxonomic	GenBank[2]	Gene	protein	length	Reference
	**Phylum proteobacteria**						
1	*Sphingopyxis sp. MC1*	Alphaproteobacteria	ENY83021.1.	*EBMC1_02935*	Arachidonate 15-lipoxygenase	664	
2	*Burkholderia thailandensis E264*	Betaproteobacteria	ABC36974.1.	*BTH_I2353*	Arachidonate 15-lipoxygenase	695	6
3	*Shewanella woody MS32*	Gammaproteobacteria	ACA87192.1.	*Swoo_2919*	Arachidonate 15-lipoxygenase	725	
4	*Pseudomonas aeruginosa PAO1*	Gammaproteobacteria	AAG04558.1.	*loxA*	Arachidonate 15-lipoxygenase	685	7
5	*Pseudomonas aeruginosa 42A2*	Gammaproteobacteria	AAL85880.2.	*lox*	Linoleate 9/13-lipoxygenase	685	8
6	*Grimontia indica*	Gammaproteobacteria	EOD80348.1.	*D515_00636*	Arachidonate 15-lipoxygenase	723	
7	*Thiocapsa marina 5811*	Gammaproteobacteria	EGV20546.1.	*ThimaDRAFT_0324*	Arachidonate 15-lipoxygenase	962	
8	*Myxococcus xanthus DK 1622* (1)	Deltaproteobacteria	ABF88826.1.	*MXAN 1744*	Lipoxygenase family protein	675	This study
9	*Myxococcus xanthus DK 1622* (2)	Deltaproteobacteria	ABF86480.1.	*MXAN 1745*	Arachidonate 12 lipoxygenase	715	This study
10	*Myxococcus stipitatus Mx s8*	Deltaproteobacteria	AGC43896.1.	*MYSTI_02580*	Uncharacterized protein	690	
11	*Corallococcus coralloides M2*	Deltaproteobacteria	AFE04785.1.	*COCOR_02732*	Uncharacterized protein	687	
12	*Plesiocystis pacifica SIR-1*	Deltaproteobacteria	EDM80093.1.	*PPSIR1_20739*	Uncharacterized protein	651	
13	*Myxococcus fulvus 124B02*	Deltaproteobacteria	AKF85636.1.	*MFUL124B02_14095*	Uncharacterized protein	689	
14	*Hyalangium minutum*	Deltaproteobacteria	KFE67541.1.	*DB31_8024*	Uncharacterized protein	688	
15	*Enhygromyxa salina*	Deltaproteobacteria	KIG12340.1.	*DB30_01572*	Arachidonate 15-lipoxygenase	713	
16	*Sorangium cellulosum So0157-2*	Deltaproteobacteria	AGP37954.1.	*SCE1572_27890*	Uncharacterized protein	673	
17	*Archangium gephyra*	Deltaproteobacteria	AKI99209.1.	*AA314_00836*	Arachidonate 15-lipoxygenase	680	
18	*Cystobacter violaceus Cb vi76*	Deltaproteobacteria	KFA92210.1.	*Q664_16870*	Uncharacterized protein	656	
	**Phylum cyanobacteria**						
19	*Chamaesiphon minutus PCC 6605*	Cyanobacteria	AFY92607.1.	*Cha6605_1432*	Lipoxygenase	668	
20	*Cyanothece sp. PCC 8801*	Cyanobacteria	ACK66448.1.	*PCC8801_2437*	Linolenate 13-lipoxygenase	668	9
21	*Nostoc sp. PCC 7120*	Cyanobacteria	BAB77350.1.	*all8020*	Linolenate 9R-lipoxygenase	773	10
22	*Coleofasciculus chthonoplastes PCC 7420*	Cyanobacteria	EDX74350.1.	*MC7420_3874*	Putative uncharacterized protein	656	
23	*Acaryochloris marina MBIC 11017*	Cyanobacteria	ABW27601.1.	*AM1_2594*	Linolenate 13-lipoxygenase	571	11
24	*Scytonema tolypothrichoides VB-61278*	Cyanobacteria	KIJ84262.1.	*SD80_04430*	Uncharacterized protein	464	
	**Phylum Bacteroidetes**						
25	*Indibacter alkaliphilus LW1*	Bacteroidetes	EOZ99250.1.	*A33Q_0628*	Uncharacterized protein	729	

[1]The bacteria grouped as *Proteobacteria*, *Cyanobacteria*, and *Bacteroidetes*.

[2]The GenBank accession numbers of nucleotide sequences of LOXs.

Supplementary Table 16. Primers used for PCR analysis.

Name	Restriction enzyme	Sequence
MX 1744-F	Nde I	GCC GCA TAT GAC TGT CGA GTA CAA AC
MX 1744-R	Hind III	TAC AAG CTT TCA GAC GGT GAT GCC G
MX 1745-F	EcoR I	GGA TTC ATG AGC GCG AGT GTG A
*MX 1*745-R	Not I	GCG GCC GCT TAG ATA TTG ATG C
MX 1644-F	Nde I	GCG CAT ATG GCT GAC ATC ACG CAT CGA AC
MX 1644-R	Hind III	GCT AAG CTT TCA GGC CGG CAG CTT CTT CA
MX 5137-F	Nde I	AAC CAT ATG GCT CGC CTC GAC CCG CA
MX 5137-R	Hind III	ACA AAG CTT TCA GGC GCG CGA AAG GAT GA
MX 5217-F	Nde I	GCT GAC ATA TGG ATG AGA TGG AGG GGA CCG TC
MX 5217-R	Xho I	GAC TGC TCG AGT CAG TGA AGG AGG CTT CCC G
MX 2304-F	Nde I	GGC CAT ATG TCC ATC CAT CCA GCG GGT
MX 2304-R	Hind I	ACG AAG CTT TCA GGG CCG CGA GGC G
MX 0683-F	Nde 1	GCT CAT ATG GTT CGC TCC ACC TGC GCC
MX 0683-R	Hind 3	GCA AAG CTT TCA TGC TCC CGC CGC ATG
MX 3623-F	Nde 1	GCG CAT ATG AAC GCT CAA TCA TCA TTG CCG
MX 3623-R	Hind 3	GCG AAG CTT TCA GCC GTT GAG GAG CTG GA
MX 1745 Duet-F	EcoR I	GAT CGA ATT CAT GAG CGC GAG TGT GAC CCG GA
MX 1745 Duet-R	Hind III	GCC GCA AGC TTT TAG ATA TTG ATG CGG GAA CTG A
MX 1744 Duet-F	EcoR I	GAA TTC ATG ACT GTC GAG TAC AAA C
MX 1744 Duet-R	Hind III	TAC AAG CTT TCA GAC GGT GAT GCC G
MX 1644 Duet-F	Nde I	GCG CAT ATG GCT GAC ATC ACG CAT CGA ACC GT
MX 1644 Duet-R	Xho I	GCG CTC GAG TCA GGC CGG CAG CTT CTT CA

Supplementary Notes

Identification of hydroxy fatty acids, hepoxilins, and trioxilins. The chemical structures of fragment peaks were analysed by ChemDraw 7.0. The reaction products, hydroxy fatty acids (HFAs), obtained from polyunsaturated fatty acids (PUFAs) using whole recombinant cells expressing 12-LOX or 11-LOX from *M. xanthus* were analysed by LC-MS/MS (**Supplementary Fig. 8**). The total molecular mass of the product (MW=320.2) obtained from arachidonic acid (ARA) by whole recombinant cells expressing 11-LOX or 12-LOX was represented by a peak at *m/z* 319.2 [M−H^{-}] (**Supplementary Fig. 8a, b**). The peaks at *m/z* 167.2 and 197.1 of the 11-LOX derived product were resulted from the cleavage of the hydroxyl group at the C11 position because the chemical formulas of the fragments were $C_9H_{14}COOH$ and $C_{10}H_{15}OHCOOH$, respectively, from the hydroxyl fatty acid (HFA). Thus, these fragment peaks indicated that the compound was an 11-hydroxyeicosatetraenoic acid (11-HETE). The peaks at *m/z* 179.2 and 208.2 of the 12-LOX derived product from ARA were resulted from the cleavage of the hydroxyl group at the C12 position because the fragments indicated $C_{10}H_{14}COOH$ and $C_{11}H_{16}OHCOO^{\cdot}$, respectively, from the HFA. These results indicated that the HFA was a 12-HETE. The total molecular mass of the product (MW=318.2) obtained from EPA by whole recombinant cells expressing 11-LOX or 12-LOX was represented by a peak at *m/z* 317.2 (**Supplementary Fig. 8c, d**). The LC-MS/MS fragments of the 11-LOX derived product fragments showed the peaks at *m/z* 121.1, 151.2, and 167.8. The chemical formulas of the two peaks at *m/z* 151.2 and 167.8, which were resulted from the cleavage between C10 and C11 of the HFA, were $C_{10}H_{14}OH$ and$C_9H_{14}COOH$, respectively. A peak at *m/z* 121.1 was resulted from the cleavage between C11 and C12 because of the loss of C_9H_{13} from the HFA. These fragment peaks indicated that the HFA was an 11-hydroxyeicosapentaenoic acid (11-HEPE). The LC-MS/MS fragments of the product obtained from EPA by whole recombinant cells expressing 12-LOX showed the peaks at *m/z* 139.2 and 179.5. The chemical formulas of the two peaks, which were resulted from the cleavage between C11 and C12 of the HFA, were $C_9H_{14}OH$ and $C_{10}H_{14}COOH$, respectively. These fragment peaks identified that the HFA was a 12-HEPE. The product obtained from DHA by whole recombinant cells expressing 11- and 12-LOX showed a peak at *m/z* 343.2 as a total molecular mass (MW=344.2) of the HFA (**Supplementary Fig. 8e**). The

LC-MS/MS fragments of the 11- and 12-LOX-derived product showed the peaks at *m/z* 139.2 and 205.6, which were resulted from the cleavage between C13 and C14 of the HFA. The chemical formulas of the two peaks were $C_9H_{14}OH$ and $C_{12}H_{16}COOH$, respectively. Therefore, the compound was a 14-hydroxydocosahexaenoic acid (14-HDoHE).

Whole recombinant cells expressing 12-LOX or 11-LOX from *M. xanthus* converted PUFAs to the reaction products hepoxilins (HXs), and they were analysed by LC-MS/MS. HXs were produced from PUFAs via hydroperoxyfatty acids (HPFAs) by the twice reactions of 11- or 12-LOX. Whole recombinant cells expressing 11-LOX or 12-LOX converted ARA to the products with total molecular masses of 336.2, which were represented by the peaks at *m/z* 335.2 [M−H$^-$] (**Supplementary Fig. 9a, d, e**); and whole recombinant cells expressing 12-LOX converted EPA and DHA to the products with total molecular masses of 334.2 and 360.2, which were represented by a peak at *m/z* 333.3 and 359.4 [M−H$^-$], respectively (**Supplementary Fig. 9b, c**). The LC-MS/MS fragments of the 12-LOX derived product fragments from ARA showed the peaks at *m/z* 153.2, 183.2, and 195.2 (**Supplementary Fig. 9a**). The chemical formulas of the two peaks at *m/z* 153.2 and 183.2, which were resulted from the cleavage between C10 and C11 of the HX, were $C_{10}H_{17}O$ and $C_9H_{13}OHCOOH$, respectively. A peak at *m/z* 195.2 was resulted from the cleavage between C11 and C12 of the epoxide ring in the HX because the chemical formula was $C_{10}H_{14}OHCOO^{\cdot}$. These fragment peaks indicated that the compound was an HXB_3. The LC-MS/MS fragments of the product obtained from EPA by whole recombinant cells expressing 12-LOX showed the peaks at *m/z* 151.2, 183.2, and 223.8 (**Supplementary Fig. 9b**). Two peaks at *m/z* 151.2 and 183.2 were resulted from the cleavage between C10 and C11 of the HX and their chemical formulae were $C_{10}H_{15}O$ and $C_9H_{13}OHCOOH$, respectively. A peak at *m/z* 223.8 was resulted from the cleavage between C12 and C13 from the HX because the chemical formula was $C_{11}H_{15}OOHCOO^{\cdot}$. Thus, the fragments indicated that the HX was a HXB_4. The LC-MS/MS fragments of the 12-LOX derived product from DHA showed the peaks at *m/z* 179.1, 221.1, and 251.2 (**Supplementary Fig. 9c**). These peaks were resulted from the cleavage between C11 and C12; C13 and C14 in the epoxide; and C14 and C15 of the HX, respectively, and their chemical formulae were $C_{10}H_{14}COOH$, $C_{12}H_{16}OHCOOH$, and $C_{13}H_{17}OOHCOOH$, respectively. Thus, the HX was suggested as

an HXB_5. The LC-MS/MS fragments of the 11-LOX derived product from ARA showed the peaks at *m/z* 97.2, 127.3, 167.2, and 209.2 (**Supplementary Fig. 9d**). The peaks at *m/z* 97.1 and 167.2 were resulted from the cleavage between C10 and C11; and C13 and C14 of the HX, respectively, because the chemical formulae were C_7H_{13} and $C_9H_{14}COOH$, respectively. The peaks at *m/z* 127.3 and 209.2 were resulted from the cleavage between C12 and C13 of the HX, and the chemical formulae were $C_8H_{14}OH$ and $C_{11}H_{16}OCOOH$, respectively. Based on these fragments, this HX was suggested as a HXD_3. The LC-MS/MS fragments of the other 11-LOX derived product from ARA showed the peaks at *m/z* 139.9, 167.2, 235.1, and 265.1 (**Supplementary Fig. 9e**), which were resulted from the cleavage between C11 and C12 in the epoxide; C10 and C11; C14 and C15; and C15 and C16 of the HX, respectively. The chemical formulae of these fragments were $C_9H_{15}OH$, $C_9H_{14}COOH$, $C_{13}H_{18}OCOOH$, and $C_{14}H_{19}OOHCOOH$, respectively. These results suggested that the compound was an HXE_3.

The reaction products trioxilins (TrXs) obtained from PUFAs using whole recombinant cells expressing 11- or 12-LOX and epoxide hydrolase (EH) from *M. xanthus* were analysed by LC-MS/MS. The total molecular masses of the products (MW=354.2) obtained from ARA by whole recombinant cells expressing 11- or 12-LOX and EH were represented by the peaks at *m/z* 353.2 $[M-H^-]$ (**Supplementary Fig. 10a, d, e**), and the total molecular masses of the products (MW=352.2 and MW=378.2) obtained from EPA and DHA, respectively, by whole recombinant cells were represented by the peaks at *m/z* 351.2 and 377.2 $[M-H^-]$, respectively (**Supplementary Fig. 8b, c**). The LC-MS/MS fragments of the 12-LOX and EH derived product fragments from ARA showed the peaks at *m/z* 153.2, 201.2, and 242.1 (**Supplementary Fig. 10a**). The two peaks at *m/z* 153.2 and 201.2 were resulted from the cleavage between C9 and C10 of the TrX, and the chemical formulae were $C_8H_{12}COOH$ and $C_{11}H_{18}(OH)_3$, respectively. The chemical formulae of the peak at *m/z* 242.2 was $C_{11}H_{15}(OH)_3COO^-$, which were resulted from the cleavage between C12 and C13 of the TrX. These chemical formulae indicated that the compound is a $TrXB_3$. The LC-MS/MS fragments of the product obtained from EPA by whole recombinant cells expressing 12-LOX and EH showed the peaks at *m/z* 198.2 and 242.1 (**Supplementary Fig. 10b**). The chemical formulas of the two peaks, which were resulted from the cleavage between C9 and C10; and C12 and C13 of the TrX, were $C_{11}H_{16}(OH)_2O^-$ and $C_{11}H_{15}(OH)_3COO^-$,

respectively. These fragment peaks identified that the TrX was a $TrXB_4$. The LC-MS/MS fragments of the 12-LOX and EH derived product from DHA showed the peaks at *m*/*z* 138.2, 178.1, 198.2, and 238.1 (**Supplementary Fig. 10c**). The peaks at *m*/*z* 138.2 and 238.1 were resulted from the cleavage between C13 and C14 of the TrX because the chemical formulae were $C_9H_{14}O^{\cdot}$ and $C_{12}H_{16}(OH)_2COO^{\cdot}$, respectively. The peaks at *m*/*z* 178.1 and 198.2 were resulted from the cleavage between C11 and C12 of the TrX, and the chemical formulae were $C_{10}H_{14}COO^{\cdot}$ and $C_{11}H_{16}(OH)_2O^{\cdot}$, respectively. These results suggested that this compound was a $TrXB_5$. The LC-MS/MS fragments of the 11-LOX and EH derived product fragments from ARA showed the peaks at *m*/*z* 167.4, 187.2, 256.2, and 282.1 (**Supplementary Fig. 10d**). The peaks at *m*/*z* 167.4 and 187.2 were resulted from the cleavage between C10 and C11 of the TrX because the chemical formulae were $C_{10}H_{14}COOH$ and $C_{10}H_{16}(OH)_3$, respectively. The peaks at *m*/*z* 256.2 and 282.1 were resulted from the cleavage between C13 and C14; and C15 and C16 of the TrX, respectively, and the chemical formulae were $C_{12}H_{17}(OH)_3COO^{\cdot}$, and $C_{14}H_{19}(OH)_3COO^{\cdot}$, respectively. These fragment peaks suggested that the TrX was a $TrXD_3$. The LC-MS/MS fragments of the other 11-LOX and EH derived product from ARA showed the peaks at *m*/*z* 157.1, 167.1, 253.2, and 282.1 (**Supplementary Fig. 10e**). The peaks at *m*/*z* 157.1 and 167.1 were resulted from the cleavage of the hydroxyl group at the C11 position of the TrX because the chemical formulae were $C_9H_{15}(OH)_2$ and $C_9H_{14}COOH$, respectively. The peaks at *m*/*z* 253.2 and 282.1 were resulted from the cleavage of the hydroxyl group at the C14 position because the chemical formulae were $C_{13}H_{18}(OH)_2COOH$ and $C_{14}H_{19}(OH)_3COO^{\cdot}$, respectively. Based on these fragments, this TrX was suggested as a $TrXE_3$.

The NMR analysis of metabolites was used to confirm the structures by recording 1D and 2D NMR spectra. The overlapped peaks of compounds including double bonds were assigned by selective TOCSY and 2D (HSQC and HMBC) NMR results. A double bond is sp2, which is structurally fixed. In the case of *E*-geometry of double bond, the protons are 180 degrees apart from each other, so neither NOE peak nor ROE peak does not appear. On the other hand, in the case of *Z*-geometry of double bond, the protons are so close that the ROE peak appears[12, 13]. Based on the identified stereo information of intermediate materials (11*S*-HpETE and 12*S*-HpETE), the stereochemistry were determined by ROESY NMR results.

HXB_3 was identified as (*S*,5*Z*,8*Z*)-10-hydroxy-10-((2*R*,3*S*)-3-((*Z*)-oct-2-en-1-yl)oxiran-2-yl)deca-5,8-dienoic acid (**Supplementary Fig. 13**). The results of 1D NMR of HXB_3 were shown in **Supplementary Fig. 14** and **Supplementary Table 6**. The H-10, H-11, and H-12 had the ROE correlation with each other, indicating the syn geometry (**Supplementary Fig. 15a**). H-12 was also identified as *S*-form because 12*S*-HpETE was identified as *S*-form. H-5, H-6, H-8, H-9, H-14, and H-15 were confirmed with selective TOCSY irradiation on the peak H-3, H-10, and H-17 (**Supplementary Fig. 16**). The coupling constants of J9, J5, and J15 were ranging below 11 Hz, and H-14 and H-15 showed the ROE correlation, indicating that the double bonds have *Z* geometry (**Supplementary Fig. 15b**). C-7 and C-10 are composed of sp3 bonds, which allow both C-7 and C-10 rotate freely. As H-7 and H-10 are very close to each other in the 3D minimized energy calculated molecular structure of HXB_3, they result in ROE peaks in ROESY NMR. The 2D NMRs of HXB_3 to support additional structural analysis were shown in the **Supplementary Fig. 17**. HXB_4 was shown similar pattern with HXB_3, thus it was identified as (*S*,5*Z*,8*Z*)-10-hydroxy-10-((2*R*,3*S*)-3-((2*Z*,5*Z*)-octa-2,5-dien-1-yl)oxiran-2-yl)deca-5,8-dienoic acid (**Supplementary Fig. 18**). The 1D NMR was detected by 1H, 13C NMR (**Supplementary Fig. 19** and **Supplementary Table 7**). The H-10, H-11, and H-12 had the ROE correlation with each other, indicating the syn geometry (**Supplementary Fig. 20**). H-12 was also identified as *S*-form because 12-HpEPE was identified as *S*-form by CP-HPLC. H-5, H-6, H-8, H-9, H-14, H-15, H-17, and H-18 were confirmed with selective TOCSY irradiation on the peak H-3, H-10, H-12, and H-20 (**Supplementary Fig. 21**). The coupling constants of J5, J9, J14, and J18 were ranging below 11 Hz, indicating that the double bonds have *Z* geometry. The 2D NMRs of HXB_4 to support additional structural analysis were shown in the Supplementary Fig. 22. HXB_5 was derived from DHA and was converted via 14*S*-HpDoHE by ARA 12(*S*)-LOX (*MXAN_1745*). As HXB_5 was identified as (*S*,4*Z*,7*Z*,10*Z*)-12-hydroxy-12-((2*R*,3*S*)-3-((2*Z*,5*Z*)-octa-2,5-dien-1-yl)oxiran-2-yl)dodeca-4,7,10-trienoic acid (**Supplementary Fig. 23–24** and **Supplementary Table 8**). The H-12, H-13, and H-14 had the ROE correlation with each other, which indicates the syn geometry (**Supplementary Fig. 25**). H-14 was also identified as *S*-form because 14*S*-HpDoHE was identified as *S*-form. H-4, H-5, H-7, H-8, H-10, H-11, H-16, H-17, H-19, and H-20 were confirmed with selective TOCSY irradiation on the peak H-2, H-

6, H-12, H-14, and H-22 (**Supplementary Fig. 26**). The coupling constants of J4, J7, J11, J16, and J20 were ranging below 11 Hz, indicating that the double bonds have *Z* geometry. The support 2D NMR data of HXB_5 were shown in Supplementary figure. 27. ARA 11-LOX (*MXAN_1744*) originated from *M. xanthus* had the ability of *S*-form as stereoselectivity, meaning that all of the metabolites produced by ARA 11-LOX (*MXAN_1744*) have *S*-form stereospecificity. The main product HXD_3, which was converted from ARA by ARA 11(*S*)-LOX, was identified as (5*Z*,8*Z*)-10-((2*S*,3*R*)-3-((*S*,*Z*)-1-hydroxyoct-2-en-1-yl)oxiran-2-yl)deca-5,8-dienoic acid (**Supplementary Fig. 28**). The results of 1D NMR of HXD_3 were shown in **Supplementary Fig. 29** and **Supplementary Table 9**. The H-11, H-12, and H-13 had the ROE correlation with each other, which indicates the syn geometry (**Supplementary Fig. 30a**). H-11 was also identified as *S*-form because ARA 11(*S*)-LOX was identified as *S*-form. H-5, H-6, H-8, H-9, H-14, and H-15 were confirmed with selective TOCSY irradiation on the peak H-3, H-11, and H-13 (**Supplementary Fig. 31**). The coupling constants of J5, J9 and J14 were ranging below 11 Hz, indicating that the double bonds have *Z* geometry. The ROE correlations were showed between H-14 and 15 and between H-8 and 9 (**Supplementary Fig. 30b**). The 2D NMR data of HXD_3 to support additional structural analysis were shown in the **Supplementary Fig. 32**.

Next, we identified the 5 types of TrXs. $TrxB_3$ was confirmed as (5*Z*,8*Z*,10*S*,11*S*,12*R*,14*Z*)-10,11,12-trihydroxyicosa-5,8,14-trienoic acid (**Supplementary Fig. 33–34** and **Supplementary Table 10**). The H-10, H-11, and H-12 on $TrXB_3$ had the ROE correlation, indicating the syn geometry (**Supplementary Fig. 35a**). $TrXB_3$ was converted from HXB_3 by EH (*MXAN_1644*). H-10 of $TrXB_3$ was also identified as *S*-form because H-10 of HXB_3 was identified as *S*-form. H-5, H-6, H-8, H-9, H-14, and H-15 were confirmed with selective TOCSY irradiation on the peak H-2, H-10, and H-13 (**Supplementary Fig. 36**). The coupling constants of J5, J9 and J14 were ranging below 11 Hz, indicating that the double bonds have *Z* geometry. The ROE correlations were showed between H-14 and 15 and between H-8 and 9 (**Supplementary Fig. 35b**). The support 2D NMR data of $TrXB_3$ were shown in **Supplementary Fig. 37**. $TrXB_4$ was verified as (5*Z*,8*Z*,10*S*,11*S*,12*R*,14*Z*,17*Z*)-10,11,12-trihydroxyicosa-5,8,14,17-tetraenoic acid using NMR analysis (**Supplementary Fig. 38–39** and **Supplementary Table 11**). The H-10, H-11, and H-12 had the ROE

correlation with each other, indicating the syn geometry (**Supplementary Fig. 40a**). H-10 of $TrXB_4$ was also identified as *S*-form because H-10 of HXB_4 was identified as *S*-form. H-5, H-6, H-8, H-9, H-14, H-15, H-17, and H-18 were confirmed with selective TOCSY irradiation on the peak H-3, H-10, H-12, and H-20 (**Supplementary Fig. 41**). The coupling constants of J5, J9, J14 and J18 were ranging below 11 Hz, indication that the double bonds have *Z* geometry. The ROE correlations were showed between H-14 and 15 and between H-8 and 9 (**Supplementary Fig. 40b**). The 2D NMR data of $TrXB_4$ to support additional structural analysis were shown in the **Supplementary Fig. 42**. $TrXB_5$ was identified as (4*Z*,7*Z*,10*Z*,12*S*,13*S*,14*R*,16*Z*,19*Z*)-12,13,14-trihydroxydocosa-4,7,10,16,19-pentaenoic acid (**Supplementary Fig. 43–44** and **Supplementary Table 12**). H-12 of HXB_5 was identified as *S*-form, thus H-12 of $TrXB_5$ was also identified as *S*-form. The H-12, H-13, and H-14 had the ROE correlation with each other, which indicates the syn geometry (**Supplementary Fig. 45a**). H-4, H-5, H-7, H-8, H-10, H-11, H-16, H-17, H-19, and H-20 were confirmed with selective TOCSY irradiation on the peak H-2, H-9, H-12, H-14, and H-22 (**Supplementary Fig. 46**). The coupling constants of J4, J8, J11, J16, and J20 were ranging below 11 Hz, indicating that the double bonds have *Z* geometry. The ROE correlations were showed between H-10 and 11 and between H-16 and 17 (**Supplementary Fig. 45b**). The support 2D NMR data of $TrXB_5$ were shown in **Supplementary Fig. 47**. $TrXD_3$ was confirmed as (5*Z*,8*Z*,11*R*,12*S*,13*S*,14*Z*)-11,12,13-trihydroxyicosa-5,8,14-trienoic acid by NMR analysis (**Supplementary Fig. 48–49** and **Supplementary Table 13**). The H-11, H-12, and H-13 had the ROE correlation, which indicate the syn geometry (**Supplementary Fig. 50a**). H-13 of $TrXD_3$ was also identified as *S*-form because H-13 of HXD_3 was identified as *S*-form. H-5, H-6, H-8, H-9, H-14, and H-15 were confirmed with selective TOCSY irradiation on the peak H-3, H-11, and H-13 (**Supplementary Fig. 51**). The coupling constants of J5, J9, and J14 were ranging below 11 Hz and H-8 and H-9 showed the ROE correlation, indicating that the double bonds have *Z* geometry (**Supplementary Fig. 50b**). The 2D NMR data of $TrXD_3$ to support additional structural analysis were shown in the **Supplementary Fig. 52**. $TrXE_3$ was identified as (5*Z*,8*Z*,11*R*,12*R*,13*E*)-11,12,15-trihydroxyicosa-5,8,13-trienoic acid (**Supplementary Fig. 53–54** and **Supplementary Table 14**). The H-11 and H-12 had the ROE correlation, which indicate the syn geometry (**Supplementary Fig. 55a**). H-11 of $TrXE_3$

was also estimated as *R*-form As H-11 of HXE$_3$ was estimated as *R*-form (ex, HxD$_3$ and TrXD$_3$). The configuration of H-15 could not be defined because H-15 was too far from H-12 or H-11. H-5, H-6, H-8, H-9, H-13, and H-14 were confirmed with selective TOCSY irradiation on the peak H-3, H-10, and H-12 (**Supplementary Fig. 56**). The coupling constants of J5, J9 were ranging below 11 Hz, indicating that the double bonds have *Z* geometry whereas the coupling constants of J13, J14 were ranging upto 15 Hz, indicating that the double bonds have *E* geometry. H-8 and H-9 had the ROE correlations but H-13 and H-14 did not have any ROE correlations (**Supplementary Fig. 55b**). The 2D NMR data of TrXE$_3$ to support additional structural analysis were shown in the **Supplementary Fig. 57**.

Supplementary Methods

ESI-MS and NMR analysis. LC-MS/MS analysis of lipid mediators was performed using a Thermo-Finnigan LCQ Deca XP plus ion trap mass spectrometer (Thermo Scientific, Pittsburgh, PA, USA) at the NCIRF facility (Seoul National University, Seoul, South Korea). The instrument consisted of an LC pump, an auto sampler, and a photodiode array detector. Ionization of the samples was carried out using electrospray ionization. The operation was conducted at 275°C capillary temperature, 5 kV ion source voltage, 30 psi nebuliser gas, 46 V capillary voltage in positive mode, 15 V fragmentor voltage in negative ionization mode, 0.01 min average scan time, 0.02 min average time to change polarity, and 35% abundant precursor ions at collision energy.

The NMR studies were used to confirm the structures by recording 1D (1H, 13C, selective-TOCSY, 1H homo decoupling) and 2D (COSY, ROESY, TOCSY, HSQC, HMBC) NMR spectra on a Bruker Avance HD (850 MHz) and Avance III (600 MHz), equipped with TCI cryoprobe (NCIRF, Seoul National University). DMSO-d$_6$ and TMS were used as a solvent and an internal standard, respectively. All chemical shifts were quoted in δ (ppm).

HXB$_3$) ^{1}H NMR (600MHz, DMSO): δ 5.47 (dt, J = 10.89, 5.43 Hz, 1H), 5.41~5.27 (m, 5H), 5.04 (s, 1H), 4.07 (dd, J = 7.65, 6.03 Hz, 1H), 2.82 (td, J= 5.43, 2.17 Hz, 1H), 2.81~2.71 (m, 2H), 2.71 (dd, J = 6.03, 2.17 Hz, 1H), 2.31~2.13 (m, 2H), 2.20 (t, J = 6.44 Hz, 2H), 2.03 (td, J = 7.11, 6.52 Hz, 2H), 1.96 (dt, J = 7.10, 7.05 Hz, 2H), 1.54 (tt, J = 7.11, 6.44 Hz, 2H), 1.34~1.20 (m, 6H), 0.86 (t, J = 6.88 Hz, 3H); ^{13}C NMR (150

MHz, DMSO): δ 174.3, 132.3, 129.5, 129.4, 129.3, 128.1, 123.8, 67.1, 60.7, 54.7, 33.0, 30.8, 29.0, 28.9, 26.7, 26.1, 25.8, 24.4, 21.9, 13.9; ESI-MS (m/z): [M−H$^-$] calcd. for $C_{20}H_{31}O_4$, 335.2; found, 335.2; analysis (calcd., found for $C_{20}H_{31}O_4$): $C_{10}H_{17}O$ (153.1, 153.2), $C_9H_{13}OHCOOH$ (183.1, 183.2), $C_{10}H_{14}OHCOO\cdot$ (195.1, 195.2).

HXB$_4$) ^{1}H NMR (600MHz, DMSO): δ 5.46~5.24 (m, 8H), 4.08 (dd, J = 7.63, 6.00 Hz, 1H), 2.84 (td, J = 8.15, 2.20 Hz, 1H), 2.83~2.72 (m, 2H), 2.74 (dd, J = 6.68, 6.62 Hz, 1H), 2.72 (dd, J = 6.00, 2.20 Hz, 1H), 2.32 (m, 1H), 2.21 (m, 1H), 2.20 (t, J = 7.41 Hz, 2H), 2.04 (dt, J = 6.55, 7.02 Hz, 2H), 2.03 (dt, J = 6.95, 7.53 Hz, 2H), 1.54 (tt, J = 7.41, 7.02 Hz, 2H), 0.91 (t, J = 7.53 Hz, 3H) ; ^{13}C NMR (150 MHz, DMSO): δ 174.3, 131.6, 130.4, 129.5, 129.4, 128.1, 126.8, 124.0, 67.1, 60.7, 54.5, 33.1, 29.0, 26.1, 25.8, 25.2, 24.4, 20.0, 14.1. ESI-MS (m/z): [M−H$^-$] calcd. for $C_{20}H_{29}O_4$, 333.2; found, 333.3; analysis (calcd., found for $C_{20}H_{29}O_4$): $C_{10}H_{15}O$ (151.1, 151.2), $C_9H_{13}OHCOOH$ (183.1, 183.2), $C_{11}H_{15}OOHCOO\cdot$ (223.2, 223.8).

HXB$_5$) ^{1}H NMR (850MHz, DMSO): δ 5.51~5.23 (m, 10H), 4.09 (dd, J = 5.95, 8.34 Hz, 1H), 2.84 (m, 1H), 2.78 (m, 4H), 2.74 (t, J = 7.16 Hz, 2H), 2.72 (dd, J 2.02, 5.95 Hz, 1H), 2.32 (m, 1H), 2.24 (dt, J = 6.93 Hz, 2H), 2.21 (m, 1H), 2.20 (t, J = 6.93 Hz, 2H), 2.02 (dt, J = 7.03, 7.52 Hz, 2H), 0.92 (t, J = 7.52 Hz, 3H) ; ^{13}C NMR (212 MHz, DMSO): δ 174.2, 131.6, 130.4, 129.7, 129.6, 129.4, 128.9, 128.3, 128.1, 126.9, 124.0, 67.1, 60.7, 54.8, 34.2, 39.0, 25.2, 22.7, 20.0, 14.1. ESI-MS (m/z): [M−H$^-$] calcd. for $C_{22}H_{31}O_4$, 359.1; found, 359.4; analysis (calcd., found for $C_{22}H_{31}O_4$): $C_{10}H_{14}COOH$ (179.2, 179.1), $C_{12}H_{16}OHCOOH$ (221.2, 221.1), $C_{13}H_{17}OOHCOOH$ (251.1, 251.2).

HXD$_3$) ^{1}H NMR (600MHz, DMSO): δ 5.45~5.39 (m 2H), 5.36~5.29 (m, 4H), 4.00 (dd, J = 8.63, 6.11, 1H), 2.81 (dt, J = 2.15, 5.42 Hz, 1H), 2.74 (dd, J = 5.72, 4.17 Hz, 2H), 2.70 (dd, J = 2.15, 6.11 Hz, 1H), 2.33 (m, 1H), 2.21 (m, 1H), 2.20 (t, J = 6.38 Hz, 2H), 2.04 (dt, J = 7.08, 5.74 Hz, 2H), 1.99 (dt, J = 4.36, 6.84 Hz, 2H), 1.54 (tt, J = 6.38, 7.08 Hz, 2H), 1.31~1.19 (m, 6H), 0.85 (t, J = 7.04 Hz, 3H) ; ^{13}C NMR (150 MHz, DMSO): δ 174.4, 131.5, 130.3, 129.6, 139.2, 128.1, 124.0, 33.0, 31.1, 29.2, 26.6, 26.0, 24.4, 21.9, 13.8. ESI-MS (m/z): [M−H$^-$] calcd. for $C_{20}H_{31}O_4$, 335.2; found, 335.2; analysis (calcd., found for $C_{20}H_{31}O_4$): C_7H_{13} (97.1, 97.2), $C_8H_{14}OH$ 127.1, 127.3), $C_9H_{14}OHCOOH$ (167.1, 167.3), $C_{11}H_{16}OCOOH$ (209.1, 209.2).

HXE$_3$) ESI-MS (m/z): [M−H$^-$] calcd. for $C_{20}H_{31}O_4$, 335.2; found, 335.2; analysis (calcd., found for $C_{20}H_{31}O_4$): $C_9H_{15}OH$ (139.1, 139.9), $C_9H_{14}COOH$ (167.1, 167.2),

$C_{13}H_{18}OCOOH$ (235.3, 235.1), $C_{14}H_{19}OOHCOOH$ (265.1, 265.1).

TrXB$_3$) ^{1}H NMR (600MHz, DMSO): δ 5.53~5.43 (m, 2H), 5.41~5.27 (m, 4H), 4.50 (dd, J = 8.75, 2.92 Hz, 1H), 3.44 (dd, J = 7.27, 8.35 Hz, 1H), 3.03 (dd, J = 7.27, 2.92 Hz, 1H), 2.83 (m, 1H), 2.73 (m, 1H), 2.35 (m, 1H), 2.19 (t, J = 7.35 Hz, 2H), 2.07~1.94 (m, 5H), 1.54 (tt, J = 7.20, 7.35 Hz, 2H), 1.34~1.18 (m, 6H), 0.86 (t, J = 6.82 Hz, 3H) ; ^{13}C NMR (150 MHz, DMSO): δ 174.5, 132.2, 130.3, 128.4, 127.8, 33.7, 31.1, 30.9, 29.0, 26.6, 26.5, 24.5, 22.0, 13.9. ESI-MS (m/z): [M−H$^-$] calcd. for $C_{20}H_{33}O_5$, 353.2; found, 353.2; analysis (calcd., found for $C_{20}H_{33}O_5$): $C_8H_{12}COOH$ (153.2, 153.2), $C_{11}H_{18}(OH)_3$ (201.1, 201.2), $C_{11}H_{15}(OH)_3COO\cdot$ (242.2, 242.1).

TrXB$_4$) ^{1}H NMR (850MHz, DMSO): δ 5.52 (m, 1H), 5.51 (dd, J = 9.49, 9.25 Hz, 1H), 5.40~5.24 (m, 6H), 4.63 (dd, J = 8.74, 1.57 Hz, 1H), 3.47 (ddd, J = 8.38, 8.16, 2.77 Hz, 1H), 2.98 (m, 1H), 2.89 (dd, J = 8.16, 1.57 Hz, 1H), 2.87 (m, 1H), 2.75 (m, 2H), 2.40 (m 1H), 2.17~1.88 (m, 7H), 1.49 (m, 1H), 0.92 (t, J = 7.52 Hz, 3H); ^{13}C NMR (212 MHz, DMSO): δ 176.3, 131.9, 131.2, 129.7, 128.2, 127.9, 127.5, 127.1, 77.5, 70.0, 64.4, 35.8, 31.0, 26.6, 25.9, 25.8, 25.4, 20.0, 14.2. ESI-MS (m/z): [M−H$^-$] calcd. for $C_{20}H_{31}O_5$, 351.2; found, 351.2; analysis (calcd., found for $C_{20}H_{31}O_5$): $C_{11}H_{16}(OH)_2O\cdot$ (198.2, 198.2), $C_{11}H_{15}(OH)_3COO\cdot$ (242.2, 242.1).

TrXB$_5$) ^{1}H NMR (600MHz, DMSO): δ 5.55~5.47 (m, 2H), 5.39~5.25 (m, 8H), 4.53 (dd, J = 8.89, 2.25 Hz, 1H), 3.47 (ddd, J = 2.74, 7.49, 8.19 Hz, 1H), 3.04 (dd, J = 7.49, 2.25 Hz, 1H), 2.88 (m, 1H), 2.83~2.72 (m, 5H), 2.38 (m, 1H), 2.25 (dt, J = 6.45 Hz, 2H), 2.21 (t, J = 6.45 Hz, 2H), 2.06 (m, 1H), 2.03 (dt, J = 6.82, 7.53 Hz, 2H), 0.92 (t, J = 7.53 Hz, 3H) ; ^{13}C NMR (150 MHz, DMSO): δ 174.3, 132.1, 131.3, 128.7, 128.3, 128.0, 127.8, 127.7, 127.6, 127.4, 76.9, 70.3, 65.6, 34.3, 31.0, 25.7, 25.3, 25.1, 22.7, 20.0, 14.1. ESI-MS (m/z): [M−H$^-$] calcd. for $C_{22}H_{33}O_5$, 377.1; found, 377.2; analysis (calcd., found for $C_{22}H_{33}O_5$): $C_9H_{14}O\cdot$ (138.1, 138.2), $C_{10}H_{14}COO\cdot$ (178.1, 178.1), $C_{11}H_{16}(OH)_2O\cdot$ (198.1, 198.2), $C_{12}H_{16}(OH)_2COO\cdot$ (238.2, 238.1).

TrXD$_3$) ^{1}H NMR (850MHz, DMSO): δ 5.49 (m, 1H), 5.37~5.28 (m, 5H), 4.35 (dd, J = 7.52, 4.62 Hz, 1H), 3.32 (td, J = 7.27, 2.97 Hz, 1H), 3.26 (dd, J = 4.62, 7.27 Hz, 1H), 7.47 (m, 2H), 2.38 (m, 1H), 2.17 (t, J = 7.22 Hz, 2H), 2.10~1.96 (m, 5H), 1.53 (tt, J = 6.36, 7.22 Hz, 2H), 1.36~1.18 (m, 6H), 0.86 (t, J = 6.86 Hz, 3H); ^{13}C NMR (212 MHz, DMSO): δ 174.7, 131.1, 130.5, 129.1, 128.6, 128.3, 127.6, 76.7, 71.5, 67.7, 33.5, 31.0, 30.9, 28.8, 27.3, 26.1, 25.4, 24.7, 22.0, 13.9. ESI-MS (m/z): [M−H$^-$] calcd. for

$C_{20}H_{33}O_5$, 353.2; found, 353.2; analysis (calcd., found for $C_{20}H_{33}O_5$): $C_{10}H_{14}COOH$ (167.2, 167.4), $C_{10}H_{16}(OH)_3$ (187.1, 187.2), $C_{12}H_{17}(OH)_3COO\cdot$ (256.2, 256.2), $C_{14}H_{19}(OH)_3COO\cdot$ (282.2, 282.1).

TrXE3) ^1H NMR (850MHz, DMSO): δ 5.61 (dd, J = 15.78, 5.36 Hz, 1H), 5.55 (dd, 15.78, 6.23 Hz, 1H), 5.44 (m, 1H), 5.35~5.29 (m, 3H), 3.89 (m, 1H), 3.81 (dd, J = 4.53, 5.36 Hz, 1H), 3.29 (m, 1H), 2.74 (m, 1H), 2.71 (m, 1H), 2.19 (m, 1H), 2.10 (t, J = 7.03 Hz, 2H), 2.02 (m, 2H), 1.50 (tt, J = 6.04, 7.03 Hz, 2H), 1.41~1.18 (m, 8H), 0.85 (t, J = 7.09 Hz, 3H); ^{13}C NMR (212 MHz, DMSO): δ 175.0, 134.5, 129.8, 129.3, 128.4, 127.6, 74.2, 70.6, 37.4, 34.4, 31.4, 30.3, 26.4, 25.5, 25.1, 24.8, 22.2, 14.0. ESI-MS (m/z): [M−H$^-$] calcd. for $C_{20}H_{33}O_5$, 353.2; found, 353.2; analysis (calcd., found for $C_{20}H_{33}O_5$): $C_9H_{15}(OH)_2$ (157.2, 157.1), $C_9H_{14}COOH$ (167.2, 167.1), $C_{13}H_{18}(OH)_2COOH$ (253.2, 253.2), $C_{14}H_{19}(OH)_3COO\cdot$ (282.2, 282.1).

Supplementary References

1. Derewlany LO, Pace-Asciak CR, Radde IC. Hepoxilin A_3, hydroxyepoxide metabolite of arachidonic acid, stimulates transport of 45Ca across the guinea pig visceral yolk sac. *Can J Physiol Pharmacol* **62**, 1466-1469 (1984).
2. Pace-Asciak CR. Formation of hepoxilin A_4, B_4 and the corresponding trioxilins from 12(*S*)-hydroperoxy-5,8,10,14,17-icosapentaenoic acid. *Prostaglandins Leukot Med* **22**, 1-9 (1986).
3. Reynaud D, Pace-Asciak CR. Docosahexaenoic acid causes accumulation of free arachidonic acid in rat pineal gland and hippocampus to form hepoxilins from both substrates. *Biochim Biophys Acta* **1346**, 305-316 (1997).
4. Reynaud D, Ali M, Demin P, Pace-Asciak CR. Formation of 14,15-hepoxilins of the A_3 and B_3 series through a 15-lipoxygenase and hydroperoxide isomerase present in garlic roots. *J Biol Chem* **274**, 28213-28218 (1999).
5. Pfister SL, Spitzbarth N, Nithipatikom K, Falck JR, Campbell WB. Metabolism of 12-hydroperoxyeicosatetraenoic acid to vasodilatory trioxilin C_3 by rabbit aorta. *BBA Gen Subjects* **1622**, 6-13 (2003).
6. An JU, Kim BJ, Hong SH, Oh DK. Characterization of an ω-6 linoleate

lipoxygenase from *Burkholderia thailandensis* and its application in the production of 13-hydroxyoctadecadienoic acid. *Appl Microbiol Biotechnol* **99**, 5487-5497 (2015).

7. Vance RE, Hong S, Gronert K, Serhan CN, Mekalanos JJ. The opportunistic pathogen *Pseudomonas aeruginosa* carries a secretable arachidonate 15-lipoxygenase. *Proc Natl Acad Sci USA* **101**, 2135-2139 (2004).
8. Vidal-Mas J, Busquets M, Manresa A. Cloning and expression of a lipoxygenase from *Pseudomonas aeruginosa* 42A2. *Anton Leeuw Int J G* **87**, 245-251 (2005).
9. Andreou A, Gobel C, Hamberg M, Feussner I. A bisallylic mini-lipoxygenase from cyanobacterium *Cyanothece* sp. that has an iron as cofactor. *J Biol Chem* **285**, 14178-14186 (2010).
10. Lang I, Gobel C, Porzel A, Heilmann I, Feussner I. A lipoxygenase with linoleate diol synthase activity from *Nostoc* sp. PCC 7120. *Biochem J* **410**, 347-357 (2008).
11. Gao BL, Boeglin WE, Brash AR. ω-3 fatty acids are oxygenated at the n-7 carbon by the lipoxygenase domain of a fusion protein in the cyanobacterium *Acaryochloris marina*. *BBA Mol Cell Biol Lipids* **1801**, 58-63 (2010).
12. Jie MS, Lau MM. Novel lipidic enaminones from a C18 keto-allenic ester. *Lipids* **35**, 1135-1145 (2000).
13. John EB, Jon ON, John FO, R. H. Diastereoselective synthesis of phycocyanobilin-cysteine adducts. *J Am Chem Soc* **113**, 8024-8035 (1991).
